# A new and expeditious synthesis of all enantiomerically pure stereoisomers of rosaprostol, an antiulcer drug

**DOI:** 10.3762/bjoc.12.215

**Published:** 2016-10-21

**Authors:** Wiesława Perlikowska, Remigiusz Żurawiński, Marian Mikołajczyk

**Affiliations:** 1 Department of Heteroorganic Chemistry, Centre of Molecular and Macromolecular Studies, Polish Academy of Sciences, Sienkiewicza 112, 90-363 Łódź, Poland

**Keywords:** antiulcer drug, chiral resolution, rosaprostol, stereoselective synthesis, synthetic design

## Abstract

Four enantiomerically pure stereoisomers of rosaprostol (**1**), an antiulcer drug, were efficiently synthesized from the enantiomers of 2-(dimethoxyphosphoryl)-3-hexylcyclopentanone (**3**) as chiral substrates. The latter were obtained by resolution of racemic **3** with (+)-(*R*)-1-(1-naphthyl)ethylamine. The conversion of (+)-**3** into rosaprostol stereoisomer (−)-**1a** was accomplished in four steps in 56% overall yield. According to the same protocol, the second stereoisomer (+)-**1c** was obtained from (−)-**3** in 55% overall yield. A slightly improved procedure of the last two steps of the transformation of (+)-**3** into (−)-**1a** allowed an increase in the overall yield to 64%. The remaining two stereoisomers, (−)-**1b** and (+)-**1d**, were obtained from (−)-**1a** and (+)-**1c** in 71 and 68% yield, respectively, by a two-reaction sequence, in which a Mitsunobu inversion of configuration at C-5 was the key step.

## Introduction

**Figure 1:**
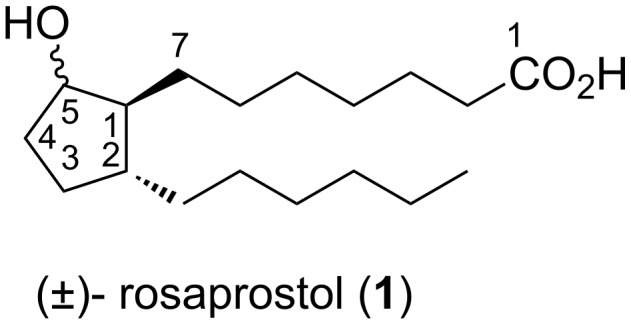
Structure of rosaprostol (**1**) and numbering system.

Rosaprostol (**1**) is a trade name for 7-(2-hexyl-5-hydroxycyclopentane)heptanoic acid (Figure 1) which belongs to a series of 19,20-dinorprostanoic acid derivatives. It exhibits gastric antisecretory activity and cytoprotective action without the undesired side effects common to other prostanoids like diarrhea, hypotension and uterine stimulation [1–8]. The sodium salt of **1** as a 1:1 mixture of the racemic 1,2-*trans*-1,5-*cis* and 1,2-*trans*-1,5-*trans* diastereoisomers has been marketed in Italy as Rosal for the treatment of gastric and duodenal ulcers [9]. Because of the therapeutical importance, the synthesis of rosaprostol (**1**) has been attempted by many research groups in academia and industry. These include a patented synthesis of **1** starting from ricinoleic acid [10], and more efficient approaches to this mixture of racemic diastereoisomers of **1** [11–17], two of which were developed by our group [15–16].

**Figure 2:**
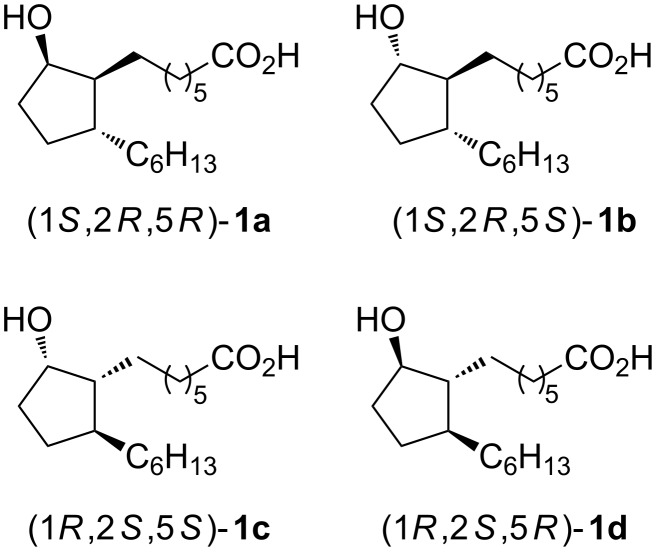
The structures of stereoisomers of rosaprostol (**1**).

Inspired by the trend in pharmaceutical sciences and industry to replace racemic by enantiomeric drugs (so-called “chiral shift”) and as part of our research program on the synthesis and study of the stereostructure–bioactivity relationship in biologically active compounds [18–19], including selected prostanoids [20–22], we decided to synthesize all four rosaprostol stereoisomers **1a–d** in enantiomerically pure form (Figure 2). The two rosaprostol stereoisomers **1c** and **1d** have an absolute configuration at C-2 of the cyclopentane ring opposite to that in natural prostaglandins of the A and J series. Therefore these compounds are expected to exhibit an increased biological stability as compared with **1a** and **1b**, and to be more efficacious in the treatment of gastric ulcers [23].

**Scheme 1:**
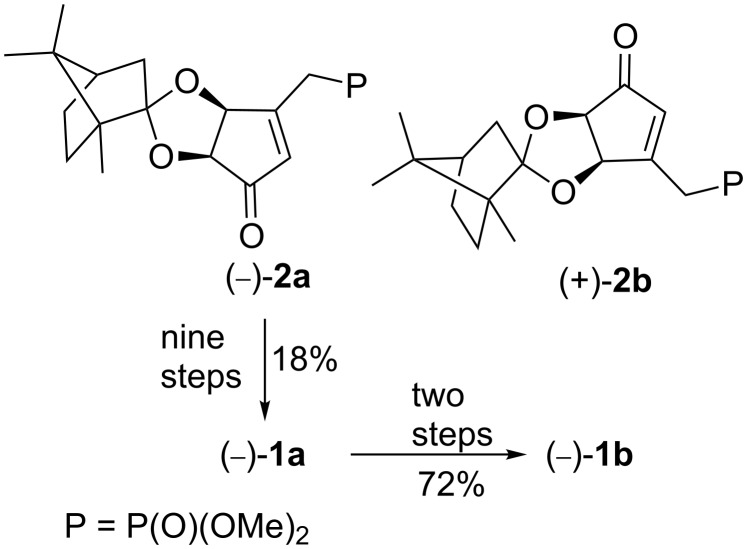
Synthesis of stereoisomeric rosaprostols **1a** and **1b** from (−)-**2a**.

Recently we devised and reported the first strategy for the synthesis of the enantiomerically pure rosaprostol stereoisomers **1a–d** using the diastereoisomerically pure camphor-protected 3-[(dimethoxyphosphoryl)methyl]-4,5-dihydroxycyclopent-2-enones (−)-**2a** and (+)-**2b** as chiral building blocks (Scheme 1) [24]. These compounds can be prepared from optically inactive *meso-*tartaric acid through a two-step reaction sequence. It involves a complete desymmetrization during an acid-catalyzed reaction with (+)-camphor and methyl orthoformate and the subsequent treatment of the formed methyl diester with α-phosphonate carbanion afforded diastereoisomeric ketals **2** in ca. 45% yield as a separable mixture.

According to this strategy, the starting substrate (−)-**2a** was converted into (−)-**1a** in nine steps in 18% overall yield. Then, the C-5 epimeric rosaprostol (−)-**1b** was prepared from (−)-**1a** in two steps in 72% yield (Scheme 1). The remaining two stereoisomers **1c** and **1d** could be obtained from (+)-**2b**, using the same sequence of reactions. Although our first synthesis allowed us to prepare the enantiomerically pure stereoisomers of rosaprostol **1** in a straightforward way, it was not fully satisfactory from the view point of efficiency and atom and step economy. Since a detailed evaluation of the biological activity and preclinical studies requires gram quantities of each stereoisomer of **1**, we sought a shorter and more efficient approach to our targets.

**Scheme 2:**
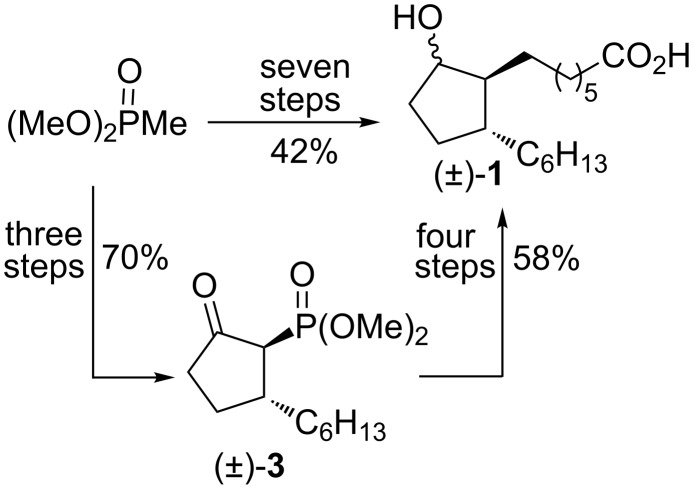
Synthesis of racemic rosaprostol (±-**1**).

Herein we disclose a new total synthesis of all enantiomerically pure rosaprostol stereoisomers **1a**–**d** in which we took advantage of our experience in the synthesis of racemic rosaprostol ((±)-**1**) [15]. This synthesis is the most efficient among all reported syntheses of (±)-**1** up to date [10–17]. As shown in Scheme 2, the conversion of a simple substrate, dimethyl methanephosphonate into (±)-**1** was achieved in only seven steps and in 42% overall yield. Racemic 2-dimethoxyphosphoryl-3-hexylcyclopentan-1-one (**3**) is the key intermediate in this synthesis. This report describes the resolution of (±)-**3** into its enantiomers and their application as starting chiral reagents for the preparation of rosaprostol stereoisomers **1a**–**d**.

## Results and Discussion

The total synthesis of stereoisomeric rosaprostols **1a–d** commenced with the resolution of racemic 2-dimethoxyphosphoryl-3-hexylcyclopentan-1-one (**3**). Thus, a solution of (±)-**3** in methylene chloride was reacted with (+)-(*R*)-1-(1-naphthyl)ethylamine in the presence of dehydrating agents. The reaction mixture was stirred for five days at room temperature and after a usual work-up the crude mixture of diastereoisomeric condensation products (^31^P NMR assay, δ_P_ = 28.08 and 28.16 ppm) was separated into pure components by column chromatography (hexane/acetone 15:1). All the spectral data indicated that the isolated diastereoisomers **4a** and **4b** have the enamine and not imine structure that should be formed in the first reaction stage. Presumably, due to electron-withdrawing properties of the phosphoryl and carbonyl groups, the α-methine hydrogen migrates to the imine nitrogen to form the cyclopent-1-ene structure.

**Scheme 3:**
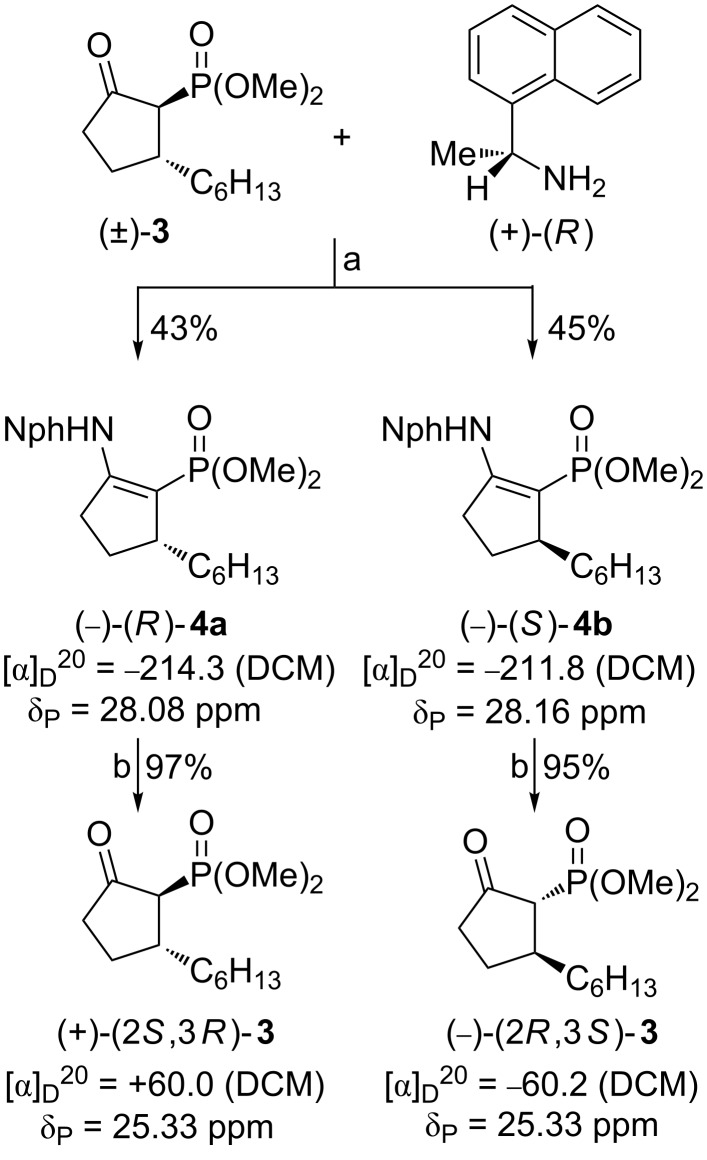
Resolution of racemic cyclopentanone **3**. Reagents and conditions: (a) Al_2_O_3_, SiO_2_, MS 5 Å, DCM, rt, 5 d; (b) cation exchanger Dowex 50WX4, MeOH/H_2_O 5:1, rt, 3 N HCl (drops), column chromatography.

The pure diastereoisomers of **4** were then efficiently converted by hydrolysis under weakly acidic conditions into the corresponding enantiomerically pure cyclopentanones (+)-**3** and (−)-**3** as the desired chiral starting materials in our new stereoselective synthesis of rosaprostols **1a**–**d**. The resolution of (±)-**3** and some experimental details are depicted in Scheme 3. It shows also the absolute configurations of (+)-**3** and (−)-**3** ascribed to the compounds based on their successful conversion into the known rosaprostols **1a** and **1c**, respectively.

**Scheme 4:**
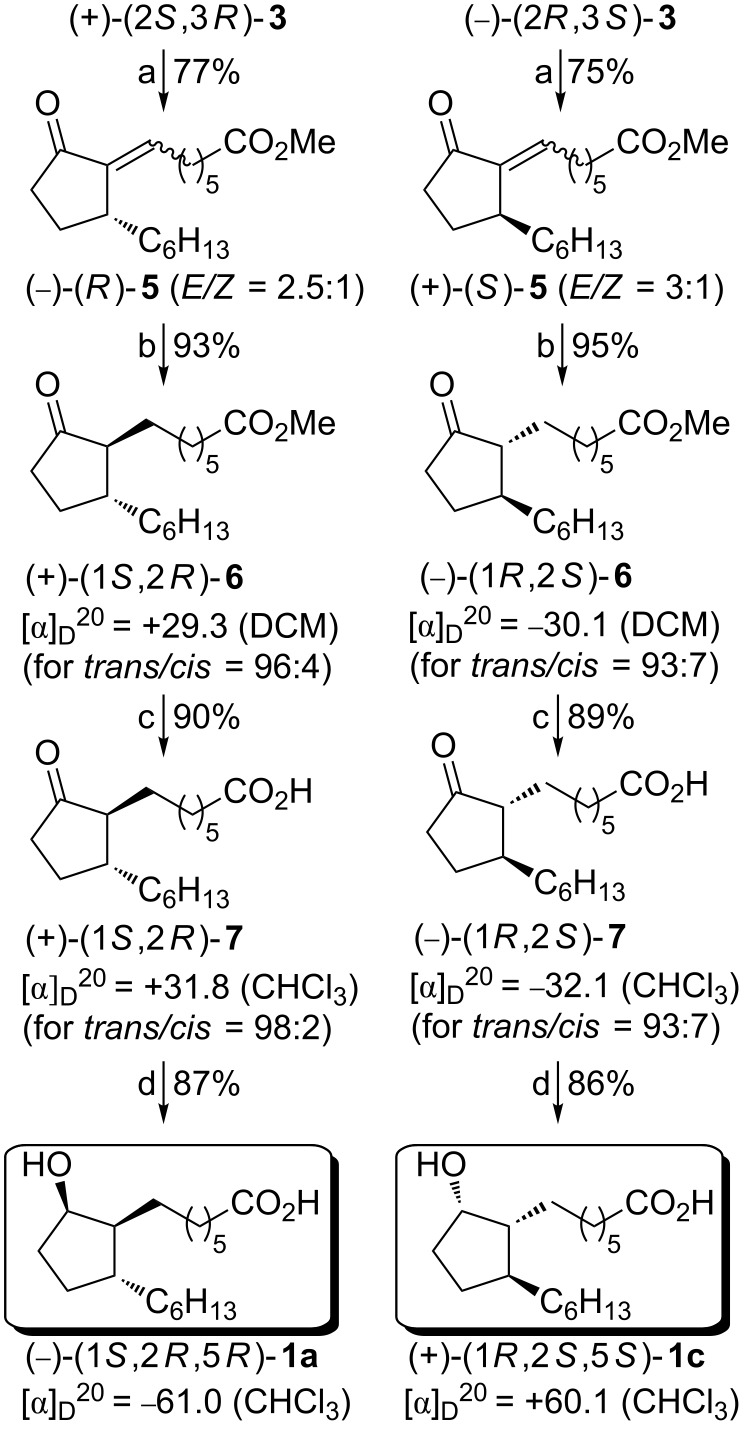
Synthesis of rosaprostol stereoisomers **1a** and **1c**. Reagents and conditions: (a) KOH/Al_2_O_3_, OHC(CH_2_)_5_CO_2_Me, C_6_H_6_, 3 h rt; (b) Te, NaBH_4_, EtOH, 6 h, rt; (c) 1 N NaOH, EtOH, 3 h, 45 °C; (d) L-Selectride, THF, −75 °C, 4 h.

Having successfully prepared the enantiomerically pure starting materials (+)-**3** and (−)-**3**, we could proceed with the synthesis of the four stereoisomers of rosaprostol (**1**). The synthetic pathway to stereoisomers **1a** and **1c** is outlined in Scheme 4.

At first, the Horner olefination reaction of (+)-**3** and (−)-**3** with methyl 5-formylpentanecarboxylate was carried out. The use of a mixture of Al_2_O_3_ and KOH as a base in this reaction afforded the corresponding olefination products (−)-(*R*)-**5** and (+)-(*S*)-**5** in high yields. Both of them were formed as mixtures of *E* and *Z*-isomers. Although these mixtures were directly used for further transformations, for characterization purposes they were separated by column chromatography. The *E/Z* ratio in (−)-(*R*)-**5** was determined by integration of the carbonyl carbon resonances in the ^13^C NMR spectra (δ_CO_ = 209.09 and 207.79 ppm for (*Z*)-(*R*)-**5** and (*E*)-(*R*)-**5**), while in the case of (+)-(*S*)-**5** by integration of the vinylic ring carbon signals (δ_(O)CC=C_ = 139.72 and 136.18 ppm for (*Z*)-(*S*)-**5** and (*E*)-(*S*)-**5**, respectively). In the next step, the exocyclic carbon–carbon double bond was selectively reduced with sodium hydrogen telluride to give the corresponding disubstituted cyclopentanone (+)-**6** containing considerable amounts of the 1,2-*cis* isomer as revealed by ^13^C NMR spectra displaying two carbonyl carbon signals (δ = 221.2 ppm for 1,2-*trans* isomer and 220.5 ppm for 1,2-*cis* isomer). The reduction product was treated with *p*-toluenesulfonic acid in methanol for a few hours to give the desired (+)-*trans*-**6** isomer contaminated with very small amounts (4%) of the *cis*-isomer. Under the same reduction/epimerization conditions, (+)-(*S*)-**5** was successfully transformed into (−)-*trans*-**6** containing 7% of the corresponding *cis* isomer. Alkaline hydrolysis of both enantiomeric methyl esters (+)-**6** and (−)-**6** afforded the ketoacids (+)-**7** and (−)-**7**, respectively. As revealed by ^13^C NMR spectoscopy, the first of them contained only 2% of the *cis*-ketoacid **7**, while in the levorotatory ketoacid **7** the content of the *cis*-isomer was slightly higher (7%). In the final step, the ketoacid (+)-**7** was reduced stereoselectively with L-Selectride under controlled temperature conditions (−75 °C, 4 h) to give the desired stereoisomeric rosaprostol as a colorless solid (mp 41–42 °C) exhibiting an optical rotation [α]_D_^20^ = −61.0 (CHCl_3_). All physicochemical properties and spectral data confirmed that the product formed in the above reaction is identical with the stereoisomer (−)-(1*S*,2*R*,5*R*)-**1a** prepared in our earlier work [23].

In summary, the rosaprostol stereoisomer (−)-**1a** was synthesized from the starting cyclopentanone phosphonate (+)-**3** in four steps and 56% overall yield. As expected, the reduction of the carbonyl group in the ketoacid (−)-**7** by L-Selectride produced the second stereoisomer of rosaprostol (+)-**1c**, [α]_D_^20^ = +60.1 (CHCl_3_). The overall yield of the four-step conversion of (−)-**3** into (+)-**1c** was 54.5%.

With regard to the assignment of the absolute configuration to (+)-**3**, it is important to notice that (−)-**1a** obtained herein has the absolute configuration *R* at the C-2 stereogenic center. As the chirality at this carbon atom remains unchanged in the four-step conversion of (+)-**3** into (−)-**1a**, the same chirality has to be assigned to the corresponding carbon atom (now C-3) in (+)-**3**. Taking into account a *trans*-arrangement of the phosphoryl and *n*-hexyl substituents, the absolute configuration (2*S*,3*R*) should be ascribed to (+)-**3** according to Cahn, Ingold, and Prelog rules [25–26]. As a consequence, the enantiomer (−)-**3** has the absolute configuration (2*R*,3*S*).

**Scheme 5:**
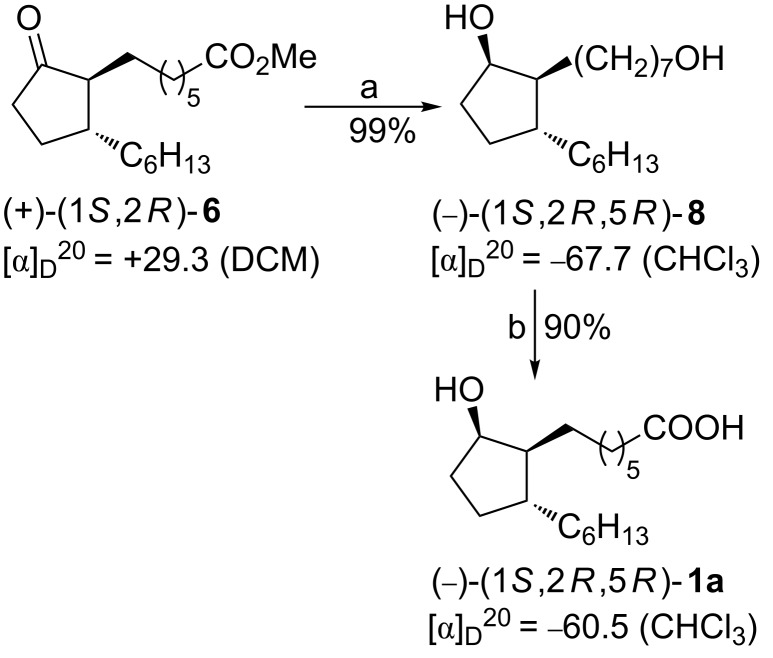
Conversion of methyl ester (+)-**6** into rosaprostol (−)-**1a**. Reagents and conditions: (a) L-Selectride, THF, −45 °C, 3 h; (b) O_2_ (air), Pd/C, NaBH_4_, KOH, H_2_O/MeOH 2:1.

In the course of our investigations on the synthesis of the stereoisomers **1a** and **1c** an alternative, more efficient method for the conversion of the enantiomeric methyl esters **6** into **1a** or **1c** was also elaborated as a two-reaction sequence. The first step comprises a reduction of **6** by L-Selectride at −45 °C affording the corresponding diol **8**. In the second step, a selective air oxidation of the primary hydroxy group in **8** was carried out according to the procedure described by Rhee and coworkers [27] to give stereoisomeric **1**. The transformation of (+)-**6** into (−)-**1a** according to the new protocol is shown in Scheme 5. It is noteworthy that the overall yield of the synthesis of (−)-**1a** from (+)-**3** involving this alternative procedure was increased to 64%.

**Scheme 6:**
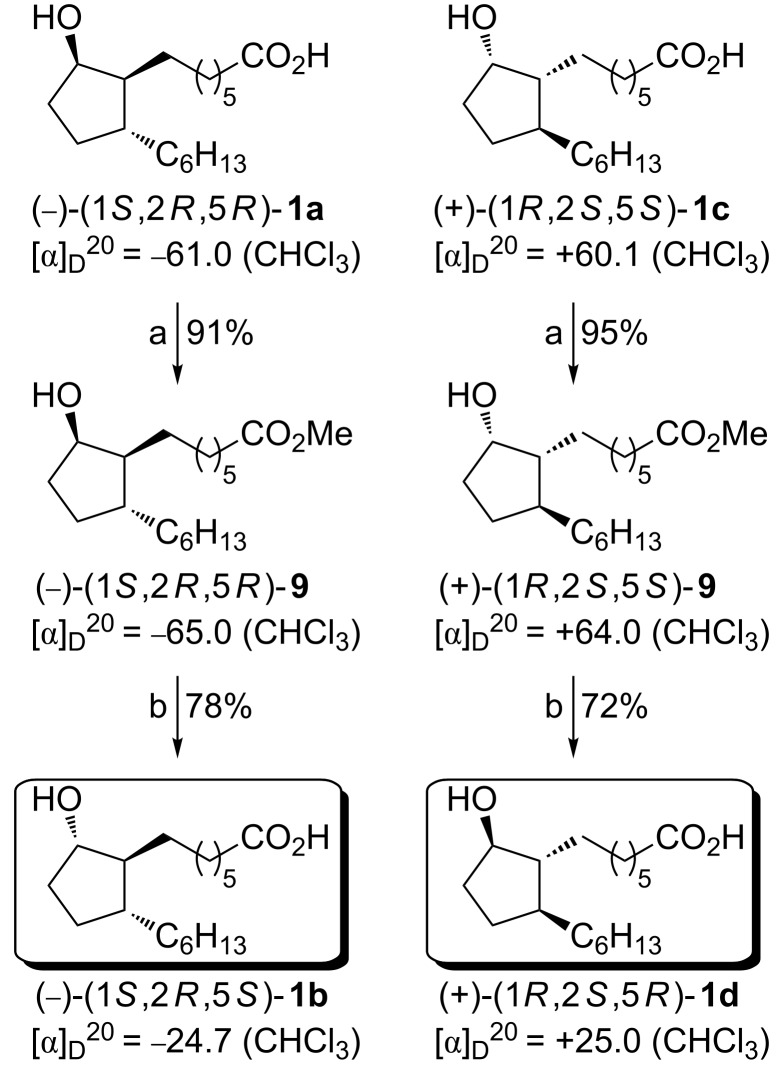
Synthesis of rosaprostol stereoisomers **1b** and **1d**. Reagents and conditions: (a) CH_2_N_2_, Et_2_O, −30 °C; (b) i. PNBA, Ph_3_P, DIAD, THF, rt; ii. LiOH, EtOH, rt, 24 h.

With the two rosaprostol stereoisomers **1a** and **1c** in hand, the synthesis of the remaining two stereoisomers **1b** and **1d** was readily accomplished with a Mitsunobu reaction [28] inverting the configuration at C-5. In fact, **1a** and **1c** were obtained through a two-step sequence starting with the methylation of (−)-**1a** and (+)-**1c** followed by a one-pot Mitsunobu esterification–hydrolysis as shown in Scheme 6. In detail, treatment of rosaprostols (−)-**1a** and (+)-**1c** with diazomethane gave the corresponding rosaprostol methyl esters (−)-**9** and (+)-**9** in yields higher than 90%. Then the esters **9** were subjected to a Mitsunobu esterification under standard conditions (THF, rt) using *p*-nitrobenzoic acid (PNBA), triphenylphosphine and diisopropyl azodicarboxylate (DIAD). The *p*-nitrobenzoic acid esters (+)-**10** and (−)-**10** formed in this reaction (not shown) were immediately hydrolyzed under basic conditions affording the two desired rosaprostol stereoisomers (−)-**1b** and (+)-**1d** in 71 and 68% yield, respectively.

Finally, for the sake of completeness of our total synthesis of stereoisomeric rosaprostols **1**, the *p*-nitrobenzoates (+)-**10** and (−)-**10** were isolated and fully characterized (see Supporting Information File 1 for details).

## Conclusion

In summary, a new concise and efficient synthesis of the four enantiomerically pure stereoisomers of rosaprostol (**1**), an antiulcer drug, was accomplished. The stereoisomers (−)-**1a** and (+)-**1c** were obtained from the enantiomeric 2-phosphoryl-3-hexylcyclopentanones (+)-**3** and (−)-**3**, respectively, in four steps in about 55% overall yield. The other two stereoisomers, (−)-**1b** and (+)-**1d**, were prepared starting from (−)-**1a** and (+)-**1c** in two steps in 70% yield. The present synthesis compares favorably in terms of the required steps (halved number) and efficiency (three-fold improvement in yield) with previous reports. It uses inexpensive reagents and operationally simple reactions. Because the synthetic sequence may be performed on a multigram-scale, it is suitable for preparation of sufficient amounts of material for biological and preclinical studies.

## Supporting Information

File 1 Experimental procedures, spectral data and copies of the ^1^H, ^13^C and ^31^ P NMR spectra for compounds **1** and **3–10** are provided.
