# Supplementary material for: A new and expeditious synthesis of all enantiomerically pure stereoisomers of rosaprostol, an antiulcer drug
Source: Beilstein J Org Chem. 2016 Oct 21;12:2234–9. doi: 10.3762/bjoc.12.215 (PMC5082602; doi:10.3762/bjoc.12.215)
Supplement: File 1 — Experimental procedures, spectral data and copies of the 1H, 13C and 31 P NMR spectra for compounds 1 and 3–10 are provided. [file Beilstein_J_Org_Chem-12-2234-s001.pdf]

Supporting Information

for

A new and expeditious synthesis of all enantiomerically pure stereoisomers of rosaprostol, an antiulcer drug

Wiesława Perlikowska, Remigiusz Żurawiński and Marian Mikołajczyk*

Address: Department of Heteroorganic Chemistry, Centre of Molecular and Macromolecular Studies, Polish Academy of Sciences, Sienkiewicza 112, 90-363 Łódź, Poland

Email: Marian Mikołajczyk - marmikol@cbmm.lodz.pl

*Corresponding author

Experimental procedures, spectral data and copies of the ^{1}H, ^{13}C and ^{31}P NMR spectra for compounds **1** and **3–10** are provided

Table of Contents

1. Experimental procedures S2
2. ^{1}H, ^{13}C and ^{31}P NMR spectra of compounds **1** and **3–10**............................... S7

Experimental procedures

General

Unless stated otherwise, all air and water-sensitive reactions were carried out under a dry argon atmosphere using freshly distilled dry solvents. Glassware was dried prior to use by heating under vacuum. Commercial grade reagents and solvents were used without further purification except as indicated below. THF was distilled over Na/benzophenone prior to use. Benzene was distilled over Na wire and CH_2Cl_2 was distilled from CaH_2. Column chromatography was performed using silica gel (70–230 mesh). NMR spectra were recorded on Bruker AV 200, Bruker DRX 500 or DRX 600 spectrometers. Chemical shifts are quoted in parts per million (ppm). ^1H and ^{13}C chemical shifts are reported relative to an external standard. ^{31}P NMR downfield chemical shifts are expressed with a positive sign relative to an external standard of 85% H_3PO_4. HRMS were recorded on a Finnigan MAT 95 apparatus. Optical rotations were measured using a Perkin-Elmer MC 241 photopolarimeter. Melting points and boiling points are uncorrected.

Dimethyl {5-hexyl-2-[(1-(naphthalen-1-yl)ethyl)amino]cyclopent-1-en-1-yl}phosphonate (−)-(4a) and (−)-(4b): To a magnetically stirred mixture of 8 g of basic aluminum oxide, 15 g of silica gel and 27 g of powdered 5 Å molecular sieves was added a solution of racemic **3** (5.9 g, 0.0184 mol) and enantiopure (+)-(*R*)-1-(1-naphthyl)ethylamine (4.11 g, 3.9 mL, 0.024 mol) in CH_2Cl_2 (155 mL). The reaction mixture was stirred at rt under nitrogen for 5 d and filtered through Celite. The resulting solution was concentrated under reduced pressure and the diastereoisomers were separated by column chromatography (hexane/acetone 15:1) to give the diastereoisomerically pure enamines (−)-(*R*)-**4a** (3.4 g, 43%) and (−)-(*S*)-**4b** (3.6 g, 45%) as colorless liquids. (−)-(*R*)-**4a**: $[\alpha]^{20}_D$= -214.3 (*c* 2.1, CH_2Cl_2); ^{31}P NMR (243 MHz, $CDCl_3$): δ 28.08; ^1H NMR (600 MHz, $CDCl_3$): δ 8.01 (d, *J* = 8.4 Hz, 1H), 7.87 (d, *J* = 8.0 Hz, 1H), 7.73 (d, *J* = 8.1 Hz, 1H), 7.60-7.42 (m, 5H), 5.26 (quint., *J* = 6.9 Hz, 1H), 3.72 (d, *J* = 9.2 Hz, 3H), 3.70 (d, *J* = 9.2 Hz, 3H), 2.63-2.57 (m, 1H), 2.57-2.49 (m, 1H), 1.98-1.91 (m, 1H), 1.75-1.61 (m, 2H), 1.58 (d, *J* = 6.8 Hz, 3H), 1.37 (m, 1H), 1.32-1.18 (m, 7H), 1.18-1.06 (m, 2H), 0.87 (t, *J* = 6.9 Hz, 3H); ^{13}C NMR (150 MHz, $CDCl_3$): δ 167.35 (d, *J* = 17.0 Hz), 141.49, 133.75, 129.91, 128.99, 127.21, 126.09, 125.81, 125.43, 122.33, 122.13, 86.11 (d, *J* = 193.1 Hz), 51.53 (d, *J* = 4.4 Hz), 51.30 (d, *J* = 4.4 Hz), 50.31, 44.27 (d, *J* = 12.5 Hz), 35.40, 31.99 (d, *J* = 19.8 Hz), 31.90, 29.49, 28.29 (d, *J* = 14.5 Hz), 27.17, 24.07, 22.61, 14.07; HRMS (EI) calcd. for $C_{25}H_{36}NPO_3$: 429.2439, found: 429.2433. (-)-(*S*)-**4b**: $[\alpha]^{20}_D$= -211.8 (*c* 2.5, CH_2Cl_2); ^{31}P NMR (81 MHz, $CDCl_3$): δ 28.16; ^1H NMR (600 MHz, $CDCl_3$): δ 8.02 (d, *J* = 8.4 Hz, 1H), 7.88 (d, *J* = 7.7 Hz, 1H), 7.74 (d, *J* = 8.1 Hz, 1H), 7.57-7.43 (m, 5H), 5.25 (p, *J* = 6.9 Hz, 1H), 3.73 (d, *J* = 2.3 Hz, 3H), 3.71 (d, *J* = 2.2 Hz, 3H), 2.70-2.64 (m, 1H), 2.45-2.37 (m, 1H), 2.10-2.03 (m, 1H), 1.93-1.85 (m, 1H), 1.66-1.60 (m, 1H), 1.59 (d, *J* = 6.8 Hz, 3H), 1.28-1.15 (m, 8H), 1.11-0.95 (m, 2H), 0.85 (t, *J* = 7.1 Hz, 3H); ^{13}C NMR (151 MHz, $CDCl_3$) δ 167.23 (d, *J* = 16.9 Hz), 141.52, 133.81, 129.95, 129.01, 127.28, 126.09, 125.72, 125.45, 122.27, 122.21, 85.56 (d, *J* = 193.2 Hz), 51.44 (d, *J* = 4.9 Hz), 51.38 (d, *J* = 4.6 Hz), 50.11, 44.23 (d, *J* = 12.5 Hz), 35.22, 32.04 (d, *J* = 19.7 Hz), 31.91, 29.49, 28.44 (d, *J* = 14.5 Hz), 27.02, 23.97, 22.57, 14.06. HRMS (EI) calcd. for $C_{25}H_{36}NPO_3$: 429.2439, found: 429.2435.

(2*S*,3*R*)-2-Dimethoxyphosphoryl-3-hexylcyclopentan-1-one (+)-(3): A solution of (−)-**4a** (3.4 g, 8.0 mmol) in MeOH/H_2O (50 mL/10 mL) was stirred at rt overnight with a weak acid cation exchanger Dowex 50 WX4 (3 g) and 4 drops of 3 N HCl. The diastereoisomers were separated by column chromatography by elution with acetone. The combined phase was evaporated and aqueous residue lyophilized to give the crude product which was purified by column chromatography (hexane/acetone 5:1) affording (+)-**3** (2.12 g, 97%) as a colorless oil; $[\alpha]^{20}_D$= +60.0 (*c* 2.5, CH_2Cl_2); ^{31}P NMR (81 MHz, $CDCl_3$): δ 25.33; ^1H NMR (600 MHz, $CDCl_3$): δ 3.81-3.69 (m, 6H), 2.62-2.50 (m, 1H), 2.38 (dd, *J* = 26.3, 7.8 Hz, 1H), 2.34-2.21 (m, 3H), 1.73-1.66 (m, 1H), 1.52-1.44 (m, 1H), 1.39-1.31 (m, 1H), 1.30-1.18 (m, 8H), 0.84 (t, *J* = 6.7 Hz, 3H); ^{13}C NMR (151 MHz, $CDCl_3$): δ 211.94 (d, *J* = 3.7 Hz, *C*=O), 53.38 (d, *J* = 6.7 Hz), 52.72 (d, *J* = 136.4 Hz), 52.68 (d, *J* = 6.7 Hz), 38.61 (d, *J* = 3.5 Hz), 38.55, 35.37 (d, *J* = 5.5 Hz), 31.65, 29.10, 27.72 (d, *J* = 11.0 Hz), 26.73, 22.51, 13.99.

(2*R*,3*S*)-2-Dimethoxyphosphoryl-3-hexylcyclopentan-1-one (−)-(3): According to the procedure described above, enamine (−)-**4b** was hydrolyzed to phosphonate (−)-**3** in 95% yield. Colorless oil; $[\alpha]^{20}_{D}$= -60.2 (*c* 2.4, CH_2Cl_2); ^{31}P NMR (81 MHz, $CDCl_3$): δ 25.33; ^{1}H NMR (600 MHz, $CDCl_3$): δ 3.81-3.72 (m, 6H), 2.63-2.52 (m, 1H), 2.39 (dd, *J* = 26.2, 7.7 Hz, 1H), 2.35-2.23 (m, 3H), 1.75-1.67 (m, 1H), 1.55-1.45 (m, 1H), 1.42-1.33 (m, 1H), 1.33-1.19 (m, 8H), 0.89-0.83 (m, 3H); ^{13}C NMR (151 MHz, $CDCl_3$): δ 211.96 (d, *J* = 3.0 Hz, *C*=O), 53.40 (d, *J* = 6.7 Hz), 52.73 (d, *J* = 136.4 Hz), 52.70 (d, *J* = 6.7 Hz), 38.62 (d, *J* = 3.4 Hz), 38.56, 35.38 (d, *J* = 5.5 Hz), 31.66, 29.11, 27.73 (d, *J* = 11.0 Hz), 26.74, 22.52, 14.00; HRMS (EI) calcd. for $C_{13}H_{25}PO_4$: 276.1492, found: 276.1491.

Methyl 7-[(2*S*)-2-hexyl-5-oxocyclopentylidene]heptanoate (+)-(5): To a stirred solution of phosphonate (−)-**3** (1.8 g, 6.5 mmol) and methyl 5-formylpentanecarboxylate (1.14 g, 7.2 mmol) in benzene (50 mL) at rt was added Al_2O_3/KOH [obtained by evaporation of a mixture of Al_2O_3 (1.1 g) and saturated aqueous solution of KOH (0.37 g, 6.6 mmol) under reduced pressure]. After stirring for 3 h, the reaction mixture was filtered through a Celite pad, concentrated in vacuo and subjected to column chromatography (hexane/acetone 20:1) affording (+)-**5** (1.51 g, 75%) as a mixture of isomers (*E*:*Z*=3:1). For analytical purposes isomers were separated. (+)-(*E*)-(*S*)-**5**: R_f=0.30 (hexane:acetone 20:1); $[\alpha]^{20}_{D}$= +105.8 (*c* 2.3, CH_2Cl_2); ^{1}H NMR (500 MHz, $CDCl_3$): δ 6.46 (t, *J* = 7.6 Hz, 1H), 3.63 (s, 3H), 2.88 (q, *J* = 6.7 Hz, 1H), 2.40-2.18 (m, 4H), 2.18-2.09 (m, 2H), 1.94-1.75 (m, 2H), 1.66-1.54 (m, 2H), 1.49-1.41 (m, 2H), 1.41-1.30 (m, 5H), 1.30-1.19 (m, 7H), 0.85 (t, *J* = 6.4 Hz, 3H); ^{13}C NMR (126 MHz, $CDCl_3$): δ 207.74, 173.97, 141.99, 136.18, 51.40, 38.42, 35.97, 34.57, 33.81, 31.74, 29.27, 29.08, 28.81, 28.33, 27.45, 24.75, 24.63, 22.54, 14.00; HRMS (CI) $(M+H)^+$ calcd. for $C_{19}H_{32}O_3$: 309.2430, found: 309.2434. (+)-(*Z*)-(*S*)-**5**: **:** R_f=0.35 (hexane:acetone 20:1); $[\alpha]^{20}_{D}$= +12.5 (*c* 1.0, CH_2Cl_2); ^{1}H NMR (500 MHz, $CDCl_3$): δ 5.82 (td, *J* = 7.5, 2.2 Hz, 1H), 3.65 (s, 3H), 2.71-2.54 (m, 2H), 2.35-2.25 (m, 3H), 2.25-2.13 (m, 1H), 2.13-2.00 (m, 1H), 1.67-1.54 (m, 3H), 1.48-1.21 (m, 15H), 0.88 (t, *J* = 6.8 Hz, 3H); ^{13}C NMR (126 MHz, $CDCl_3$): δ 209.19, 174.19, 140.03, 139.72, 51.43, 42.17, 38.51, 34.54, 33.95, 31.80, 29.39, 29.03, 28.76, 27.47, 26.77, 26.40, 24.75, 22.61, 14.06; HRMS (CI) $(M+H)^+$ calcd. for $C_{19}H_{32}O_3$: 309.2430, found: 309.2430.

Methyl 7-[(2*R*)-2-hexyl-5-oxocyclopentylidene]heptanoate (−)-(5): According to the procedure described above, phosphonate (−)-**3** (1.7 g, 6.2 mmol) was transformed into (−)-**5** (1.46 g, 77%) as a mixture of isomers (*E*:*Z*=2.5:1). For analytical purposes isomers were separated. (−)-(*E*)-(*R*)-**5**:; $[\alpha]^{20}_{D}$= −105.9 (*c* 2.9, CH_2Cl_2); (-)-(*Z*)-(*R*)-**5:** $[\alpha]^{20}_{D}$= -12.8 (*c* 2.7, CH_2Cl_2). ^{1}H and ^{13}C NMR spectra were consistent with the corresponding spectra of (+)-(*E*)-(*S*)-**5** and (+)-(*Z*)-(S)-**5**.

Methyl 7-[(1*S*,2*R*)-2-hexyl-5-oxocyclopentyl]heptanoate (+)-(6): To a magnetically stirred mixture of tellurium powder (1.6 g, 12.5 mmol) in ethanol (50 mL) under a positive pressure of an argon was added sodium borohydride (0.615 g, 16.3 mmol) in one portion. The reaction mixture was warmed to 40 °C and when a clear, almost colorless solution had formed, the temperature was lowered to 0 °C and acetic acid (0.6 mL) was added. Then a solution of (−)-(*R*)-***5*** (1.32 g, 4.3 mmol) in ethanol (3 mL) was added and the reaction mixture was stirred for 6 h at rt, then filtered through a Celite pad and concentrated. The residual liquid was purified by column chromatography (hexane/acetone 15:1) affording a mixture of *trans* and *cis* isomers as evidenced by the ^{13}C NMR spectra (two carbonyl signals at δ = 221.2 ppm and 220.5 ppm for *trans* and *cis* isomers, respectively). The mixture was dissolved in MeOH (12 mL) and treated with *p*-toluenesulfonic acid (0.09 g). After stirring overnight the reaction mixture was neutralized with $NaHCO_3$, filtered and evaporated. The crude product was purified by column chromatography (hexane/acetone 15:1) affording a mixture of (+)-(1*S*,2*R*)-**6** and its (1*R*)-epimer (1.23 g, 93%) as a colorless liquid in a ratio of 96:4. (+)-(1*S*,2*R*)-**6**: $[\alpha]^{20}_{D}$ = +29.3 (*c* 2.3, CH_2Cl_2) (for trans:cis = 96:4); ^{1}H NMR (500 MHz, $CDCl_3$): δ 3.62 (s, 3H, CH_3O), 2.26 (t, *J* = 7.4 Hz, 2H, $CH_2CO_2CH_3$), 2.16-1.97 (m, 2H, CH_2 (ring)), 1.83-1.72 (m, 1H, C*H* (ring)), 1.69-1.52 (m, 4H), 1.52-1.44 (m, 2H), 1.44-1.10 (m, 17H), 0.90-0.80 (m, 3H, CH_3CH_2); ^{13}C NMR (126 MHz, $CDCl_3$): δ 221.38, 174.15, 54.94, 51.33, 41.47, 37.80, 34.69, 33.94, 31.75, 29.48, 29.42, 28.88, 27.88, 26.99, 26.95, 26.59, 24.80, 22.55, 14.01; HRMS (EI) calcd. for $C_{19}H_{34}O_3$: 310.2495, found: 310.2507.

Methyl 7-[(1*R*,2*S*)-2-hexyl-5-oxocyclopentyl]heptanoate (−)-(6): According to the procedure described above, enone (+)-**5** (1.28 g, 0.0042 mol) was reduced to a mixture of (−)-(1*R*,2*S*)-**6** and its (1*R*)-epimer (1.22 g, 95%) in a ratio of 93:7. (−)-(1*R*,2*S*)-**6:** $[\alpha]^{20}_{D}$ = -30.1 (*c* 3.5, CH_2Cl_2) (for *trans*:*cis* = 93:7); ^{1}H NMR (500 MHz, $CDCl_3$): δ 3.62 (s, 3H), 2.25 (t, *J* = 7.5 Hz, 2H), 2.15-1.98 (m, 2H), 1.81-1.72 (m, 1H), 1.66-1.51 (m, 4H), 1.51-1.44 (m, 2H), 1.44-1.13 (m, 17H), 0.85 (t, *J* = 6.7 Hz, 3H); ^{13}C NMR (126 MHz, $CDCl_3$): δ 221.37, 174.13, 54.93, 51.32, 41.45, 37.79, 34.68, 33.92, 31.73, 29.47, 29.41, 28.86, 27.85, 26.98, 26.94, 26.58, 24.78, 22.54, 13.99.

7-[(1*R*,2*S*)-2-Hexyl-5-oxocyclopentyl]heptanoic acid (−)-(7): A solution of (−)-**6** (0.679 g, 2.2 mmol) in 95% ethanol (26 mL) was heated at 45 °C with 1 N NaOH (13 mL). After 3 h, the reaction mixture was concentrated under reduced pressure, acidified by the addition of 5% aqueous HCl and extracted with chloroform. The organic extracts were dried over Na_2SO_4 and concentrated. The crude product was purified by column chromatography (hexane/acetone 2:1) to yield acid (−)-**7** (0.58 g, 89%). $[\alpha]^{20}_{D}$= −32.1 (*c* 2.8, $CHCl_3$); ^{1}H NMR (500 MHz, $CDCl_3$): δ 2.33 (t, *J* = 7.5 Hz, 2H, C*H*$_2$CO$_2$H), 2.30-2.26 (m, 1H), 2.19-2.01 (m, 2H, C*H*$_2$ (ring)), 1.86-1.75 (m, 1H, C*H* (ring)), 1.71-1.57 (m, 4H), 1.52 (q, *J* = 13.9, 7.3 Hz, 2H), 1.45-1.15 (m, 17H), 0.88 (t, *J* = 6.9 Hz, 3H, C*H*$_3$CH$_2$); ^{13}C NMR (126 MHz, $CDCl_3$): δ 221.80 (*C*=O), 179.95 (*C*OOH), 55.02, 41.52, 37.87, 34.73, 33.97, 31.80, 29.51, 29.47, 28.83, 27.92, 27.04, 27.00, 26.62, 24.57, 22.60, 14.06; HRMS (EI) calcd. for $C_{18}H_{32}O_3$: 296.2351, found: 296.2352.

7-[(1*S*,2*R*)-2-Hexyl-5-oxocyclopentyl]heptanoic acid (+)-(7): According to the procedure described above methyl ester (+)-**6** (0.687 g, 2.4 mmol) was hydrolyzed to give acid (+)-**7** (0.649 g, 90%). $[\alpha]^{20}_{D}$= +31.8 (*c* 2.2, $CHCl_3$); ^{1}H NMR ($CDCl_3$, 500 MHz): δ 2.33 (t, *J* = 7.5 Hz, 3H), 2.29 (d, *J* = 8.9 Hz, 1H), 2.20-2.01 (m, 2H), 1.86-1.75 (m, 1H), 1.73-1.56 (m, 4H), 1.52 (dd, *J* = 13.9, 7.2 Hz, 2H), 1.45-1.18 (m, 16H), 0.88 (t, *J* = 6.7 Hz, 3H); ^{13}C NMR ($CDCl_3$, 125 MHz): δ 221.8, 179.9, 55.0, 41.5, 37.9, 34.7, 34.0, 31.8, 29.5, 29.5, 28.8, 27.9, 27.0, 27.0, 26.6, 24.6, 22.6, 14.1.

7-[(1*R*,2*S*,5*S*)-2-Hexyl-5-hydroxycyclopentyl]heptanoic acid (+)-(1c): To a stirred solution of acid (−)-**7** (0.424 g, 1.4 mmol) in THF (30 mL) at −75 °C was added dropwise L-Selectride (3 mL, 1 M in THF, 3 mmol) under an argon atmosphere. After 4 h at −75 °C the mixture was hydrolyzed with saturated NH_4Cl solution and left standing overnight. The organic layer was decanted and the aqueous layer extracted with Et_2O (3 × 20 mL). The combined organic layers were dried over $MgSO_4$, filtered, evaporated under reduced pressure and the residue was purified by column chromatography (hexane/acetone 1:1) to afford (+)-**1c** (0.369 g, 86%). $[\alpha]^{20}_{D}$= +60.1 (*c* 2.1, $CHCl_3$); ^{1}H NMR (500 MHz, $CDCl_3$): δ 6.47 (bs, 1H, CO$_2$*H*), 4.19 (t, *J* = 3.9 Hz, 1H, C*H*OH), 2.32 (t, *J* = 7.5 Hz, 2H, C*H*$_2$CO$_2$H), 2.01-1.91 (m, 1H), 1.85-1.75 (m, 1H, C*H* (ring)), 1.68-1.53 (m, 4H), 1.53-1.44 (m, 1H), 1.44-1.06 (m, 19H), 1.06-0.96 (m, 1H), 0.86 (t, *J* = 6.9 Hz, 3H, C*H*$_3$CH$_2$); ^{13}C NMR (126 MHz, $CDCl_3$): δ 179.31, 74.54, 51.43, 41.79, 35.07, 34.01, 33.32, 31.88, 29.61 (2 C), 28.96, 28.89, 28.28, 28.11, 27.56, 24.59, 22.64, 14.08; HRMS (EI) calcd. for $C_{18}H_{34}O_3$: 297.2429, found: 297.2436.

7-[(1*S*,2*R*,5*R*)-2-Hexyl-5-hydroxycyclopentyl]heptanoic acid (−)-(1a): According to the procedure described above ketoacid (+)-**7** (0.377 g, 1.3 mmol) was transformed into rosaprostol stereoisomer (−)-**1a** (0.330 g, 87%). $[\alpha]^{20}_{D}$= −61.0 (*c* 2.6, $CHCl_3$); mp = 41-42 °C; ^{1}H NMR (500 MHz, $CDCl_3$): δ 6.41 (bs, 1H), 4.15 (s, 1H), 2.27 (t, *J* = 7.4 Hz, 1H), 1.97-1.86 (m, 1H), 1.80-1.70 (m, 1H), 1.62-1.50 (m, 3H), 1.50-1.05 (m, 14H), 1.05-0.93 (m, 1H), 0.82 (t, *J* = 6.2 Hz, 2H); ^{13}C NMR ($CDCl_3$, 125 MHz) δ 179.5, 74.5, 51.4, 41.8, 35.1, 34.0, 33.4, 31.9, 29.6 (2 C), 29.0, 28.9, 28.3, 28.1, 27.6, 24.6, 22.7, 14.1.

7-[(1*R*,2*R*,5*R*)-2-Hexyl-5-hydroxycyclopentyl]heptan-1-ol (8): To a stirred solution of (+)-**6** (688 mg, 2.22 mmol) in THF (30 mL) at −70 °C was added dropwise L-Selectride (1 M in THF, 7 mL, 7 mmol) under an argon atmosphere. The mixture was stirred at −70 °C for 0.5 h and then at −45 °C for 3 h and then saturated aqueous solution of NH_4Cl was added. After separation of the organic layer, the aqueous solution was extracted with Et_2O (3 × 20 mL). The combined organic extracts were dried over anhydrous $MgSO_4$, filtered, concentrated under reduced pressure and the residue was subjected to column chromatography (petroleum ether/acetone 4:1) affording **8** (687 mg, 99%) as a colorless oil.

$[\alpha]_D^{20}$ = −67.7 (*c* 2.2, $CHCl_3$); ^1H NMR (600 MHz, $CDCl_3$): δ 4.18-4.16 (m, 1H), 3.6 (t, *J* = 6.6 Hz, 2H), 1.98-1.77 (m, 4H), 1.66-1.46(m, 6H), 1.44-1.07(m, 19H), 1.04-0.95(m,1H), 0.86 (t, *J* = 6.8 Hz, 3H); ^{13}C NMR (126 MHz, $CDCl_3$): δ 74.38, 62.82, 51.44, 41.85, 35.08, 33.47, 32.66, 31.86, 29.95, 29.60, 29.27, 28.94, 28.26, 28.26, 27.62, 25.62, 22.62, 14.05; HRMS (EI) (M-H_2O) calcd. for $C_{18}H_{34}O_2$: 266.2600, found: 266.2609.

7-[(1*S*,2*R*,5*R*)-2-Hexyl-5-hydroxycyclopentyl]heptanoic acid (−)-(1a): To a suspension of 10% Pd/C (26 mg, 0.244 mmol) in water (26 mL) was added sodium borohydride (20 mg, 0.53 mmol), potassium hydroxide (814 mg, 15.5 mmol) and a solution of diol **8** (686 mg, 2.42 mmol) in MeOH (13 mL). The reaction mixture was vigorously stirred in open air atmosphere at room temperature for 48 h. Then, the mixture was acidified with 0.1 M aqueous HCl and extracted with $CHCl_3$ (5 × 20 mL). The organic extracts were dried over MgSO4, concentrated under reduced pressure and the residue was purified by column chromatography (petroleum ether/acetone 2:1) affording (−)-**1a** (649 mg, 90%). $[\alpha]_D^{20}$ = −60.5 (*c* 3.2, $CHCl_3$); mp 41-42 ^oC, ^1H NMR (500 MHz, $CDCl_3$): δ 6.50 (bs, 2H), 4.16 (m, 1H), 2.27 (t, *J* = 7.4 Hz, 2H), 1.94-1.89 (m, 1H), 1.83-1.72 (m, 1H), 1.62-1.52 (m, 4H), 1.50-1.12 (m, 19H), 1.09-0.94 (m, 1H), 0.82 (t, *J* = 6.8 Hz, 3H); ^{13}C NMR (125 MHz, $CDCl_3$): δ 178.73, 74.39, 51.37, 41.71, 35.02, 33.93, 33.22, 31.80, 29.54, 29.54, 28.91, 28.82, 28.21, 28.05, 27.49, 24.56, 22.56, 13.99.

Methyl 7-[(1*R*,2*S*,5*S*)-2-hexyl-5-hydroxycyclopentyl]heptanoate (+)-(9): To a magnetically stirred ethereal solution of diazomethane (0.078 g, 0.0019 mol, 1.5 equiv) at −30 °C was slowly added rosaprostol stereoisomer (+)-**1c** (0.368 g, 0.0012 mol) in Et_2O (10 mL). The yellow solution was stirred overnight, the solvent removed under reduced pressure and the crude product purified by column chromatography (hexane/acetone 15:1) affording methyl ester (+)-**9** (0.368 g, 95%) as a colorless liquid. $[\alpha]^{20}_D$= +64.0 (*c* 2.1, $CHCl_3$); ^1H NMR (500 MHz, $CDCl_3$): δ 4.15 (t, *J* = 4.0 Hz, 1H, C*H*OH), 3.62 (s, 3H, OC*H*$_3$), 2.27 (t, *J* = 7.5 Hz, 2H, C*H*$_2$$CO_2CH_3$), 1.99-1.89 (m, 1H), 1.84-1.73 (m, 1H, C*H* (ring)), 1.62-1.52 (m, 4H), 1.52-1.09 (m, 20H), 1.04-0.93 (m, 1H), 0.84 (t, *J* = 6.8 Hz, 3H, C*H*$_3$$CH_2$); ^{13}C NMR (126 MHz, $CDCl_3$): δ 174.26, 74.24, 51.41, 51.37, 41.76, 35.04, 33.96, 33.44, 31.83, 29.58, 29.57, 29.00, 28.89, 28.24, 28.11, 27.53, 24.77, 22.60, 14.03; HRMS (EI) (M-H_2O) calcd. for $C_{19}H_{34}O_2$: 294.2551, found: 294.2559.

Methyl 7-[(1*S*,2*R*,5*R*)-2-hexyl-5-hydroxycyclopentyl]heptanoate (−)-(9): According to the procedure described above rosaprostol stereoisomer (−)-**1a** (0.33 g, 1.1 mmol) was transformed into methyl ester (−)-**9** (0.314 g, 91%) as a colorless liquid. $[\alpha]^{20}_D$= −65.0 (*c* 2.4, $CHCl_3$); ^1H NMR (500 MHz, $CDCl_3$): δ 4.17 (t, *J* = 4.4 Hz, 1H, C*H*OH), 3.64 (s, 3H, OC*H*$_3$), 2.29 (t, *J* = 7.5 Hz, 2H, C*H*$_2$$CO_2CH_3$), 2.00-1.90 (m, 1H), 1.85-1.75 (m, 1H), 1.65-1.54 (m, 4H), 1.53-1.12 (m, 20H), 1.05-0.96 (m, 1H), 0.86 (t, *J* = 6.9 Hz, 3H, C*H*$_3$$CH_2$); ^{13}C NMR (126 MHz, $CDCl_3$): δ 174.38, 74.42, 51.52, 51.49, 41.89, 35.14, 34.08, 33.57, 31.94, 29.70, 29.68, 29.11, 29.01, 28.35, 28.23, 27.65, 24.89, 22.71, 14.14.

7-[(1*R*,2*S*,5*R*)-2-Hexyl-5-hydroxycyclopentyl]heptanoic acid (+)-(1d): A stirred suspension of methyl ester (+)-**9** (216 mg, 0.693 mmol), Ph_3P (236 mg, 0.91 mmol) and *p*-nitrobenzoic acid (PNBA, 127 mg, 0.762 mmol) in THF (10 mL) was placed in an ice-water bath for 15 min. Then, diisopropylazodicarboxylate (DIAD, 168 mg, 0.831 mmol) was added dropwise. The yellow homogenous mixture was stirred at 0 °C for 30 min, then at rt overnight. The solvent was removed in vacuo and the crude product was dissolved in EtOH (7 mL). A 1 M aq. solution of LiOH (6 mL) was added at rt and the mixture was stirred overnight. EtOH was evaporated in vacuo, the residue was acidified by addition of 5% aq. solution of HCl and extracted with $CHCl_3$ (3 × 20 mL). The organic phase was dried over $MgSO_4$, concentrated, and the crude product was purified by column chromatography (hexane/acetone 2:1) to yield rosaprostol stereoisomer (−)-**1d** (148 mg, 72%). $[\alpha]^{20}_D$= +25.0 (*c* 0.6, $CHCl_3$); ^1H NMR (500 MHz, $CDCl_3$): δ 6.21 (bs, 1H), 3.91-3.84 (m, 1H), 2.33 (t, *J* = 7.5 Hz, 2H), 1.82-1.68 (m, 2H), 1.68-1.54 (m, 3H), 1.54-1.43 (m, 1H), 1.43-1.13 (m, 21H), 0.87 (t, *J* = 6.8

Hz, 3H); ^{13}C NMR (126 MHz, $CDCl_3$): δ 179.38, 79.26, 54.39, 44.59, 35.98, 34.20, 34.01, 33.49, 31.88, 29.59, 29.55, 29.34, 28.97, 28.26, 27.61, 24.61, 22.65, 14.09.

7-[(1*S*,2*R*,5*S*)-2-Hexyl-5-hydroxycyclopentyl]heptanoic acid (−)-(1b): By analogy to the procedure described above, ester (−)-**9** (211 mg, 0.675 mmol) was transformed into rosaprostol stereoisomer (−)-**1b** (157 mg, 78%). $[\alpha]^{20}_{D}$= -24.7 (*c* 1.7, $CHCl_3$); mp 28-30 ^{o}C; ^{1}H NMR (500 MHz, $CDCl_3$): δ 6.75 (bs, 1H, CO_2H), 3.92-3.81 (m, 1H, C*H*OH), 2.31 (t, *J* = 7.5 Hz, 2H, CH_2CO_2H), 1.81-1.67 (m, 2H), 1.67-1.51 (m, 3H), 1.51-1.42 (m, 1H), 1.42-1.15 (m, 21H), 0.86 (t, *J* = 6.9 Hz, 3H, CH_3CH_2); ^{13}C NMR (126 MHz, $CDCl_3$): δ 179.31, 79.19, 54.29, 44.56, 35.96, 34.12, 34.03, 33.47, 31.86, 29.59, 29.54, 29.32, 28.96, 28.25, 27.58, 24.62, 22.63, 14.08.

Methyl 7-[(1*R*,2*S*,5*R*)-2-hexyl-5-(4-nitrobenzoyloxy)cyclopentyl]heptanoate (−)-(10): A stirred suspension of methyl ester (+)-**9** (0.216 g, 0.693 mmol), Ph_3P (0.236 g, 0.91 mmol) and *p*-nitrobenzoic acid (PNBA, 0.127 g, 0.762 mmol) in THF (10 mL) was placed in an ice-water bath for 15 min. Then diisopropylazodicarboxylate (DIAD, 0.168 g, 0.831 mmol) was added dropwise. The yellow homogenous mixture was stirred at 0 °C for 30 min, then at rt overnight. The solvent was removed in vacuo and the crude product was purified by column chromatography (hexane/acetone 15:1) affording (−)-**10** (0.256 g, 80%). $[\alpha]^{20}_{D}$ = −29.6 (*c* 1.6, $CHCl_3$); ^{1}H NMR (500 MHz, $CDCl_3$): δ 8.27 (d, *J* = 8.8 Hz, 2H, $C_{Ar}H$), 8.16 (d, *J* = 8.9 Hz, 2H, $C_{Ar}H$), 5.11-5.06 (m, 2H), 3.63 (s, 3H, OCH_3), 2.26 (t, *J* = 7.5 Hz, 2H, $CH_2CO_2CH_3$), 2.02-1.85 (m, 1H), 1.82-1.73 (m, 1H), 1.71-1.63 (m, 1H), 1.62-1.46 (m, 4H), 1.46-1.20 (m, 18H), 0.87 (t, *J* = 6.8 Hz, 3H, CH_3CH_2); ^{13}C NMR (126 MHz, $CDCl_3$): δ 174.24, 164.35, 150.43, 136.26, 130.61 (2 C), 123.53 (2 C), 83.49, 51.47, 51.29, 44.48, 35.87, 34.03, 33.40, 31.91, 31.68, 30.19, 29.58, 29.54, 29.07, 28.25, 27.52, 24.92, 22.69, 14.14; HRMS (EI) calcd. for $C_{26}H_{39}NO_6$: 461.2778, found: 461.2785.

Methyl 7-[(1*S*,2*R*,5*S*)-2-hexyl-5-(4-nitrobenzoyloxy)cyclopentyl]heptanoate (+)-(10): According to the procedure described above, methyl ester (−)-**9** (0.211 g, 0.676 mmol) was transformed into diester (+)-**10** (0.256 g, 82%). $[\alpha]^{20}_{D}$= +30.2 (*c* 2.4, $CHCl_3$); ^{1}H NMR (500 MHz, $CDCl_3$): δ 8.27 (d, *J* = 8.9 Hz, 2H, $C_{Ar}H$), 8.17 (d, *J* = 8.9 Hz, 2H, $C_{Ar}H$), 5.12-5.04 (m, 2H), 3.64 (s, 3H, OCH_3), 2.26 (t, *J* = 7.5 Hz, 2H, $CH_2CO_2CH_3$), 2.01-1.86 (m, 2H), 1.82-1.75 (m, 1H), 1.72-1.65 (m, 1H), 1.63-1.47 (m, 4H), 1.47-1.21 (m, 18H), 0.87 (t, *J* = 6.8 Hz, 3H, CH_3CH_2); ^{13}C NMR (126 MHz, $CDCl_3$): δ 174.17, 164.29, 150.36, 136.19, 130.54 (2 C), 123.46 (2 C), 83.43, 51.40, 51.22, 44.41, 35.80, 33.97, 33.33, 31.84, 31.61, 30.12, 29.51, 29.48, 29.00, 28.18, 27.46, 24.86, 22.62, 14.08.

^{1}H, ^{13}C and ^{31}P NMR spectra of compounds 1 and 3–10

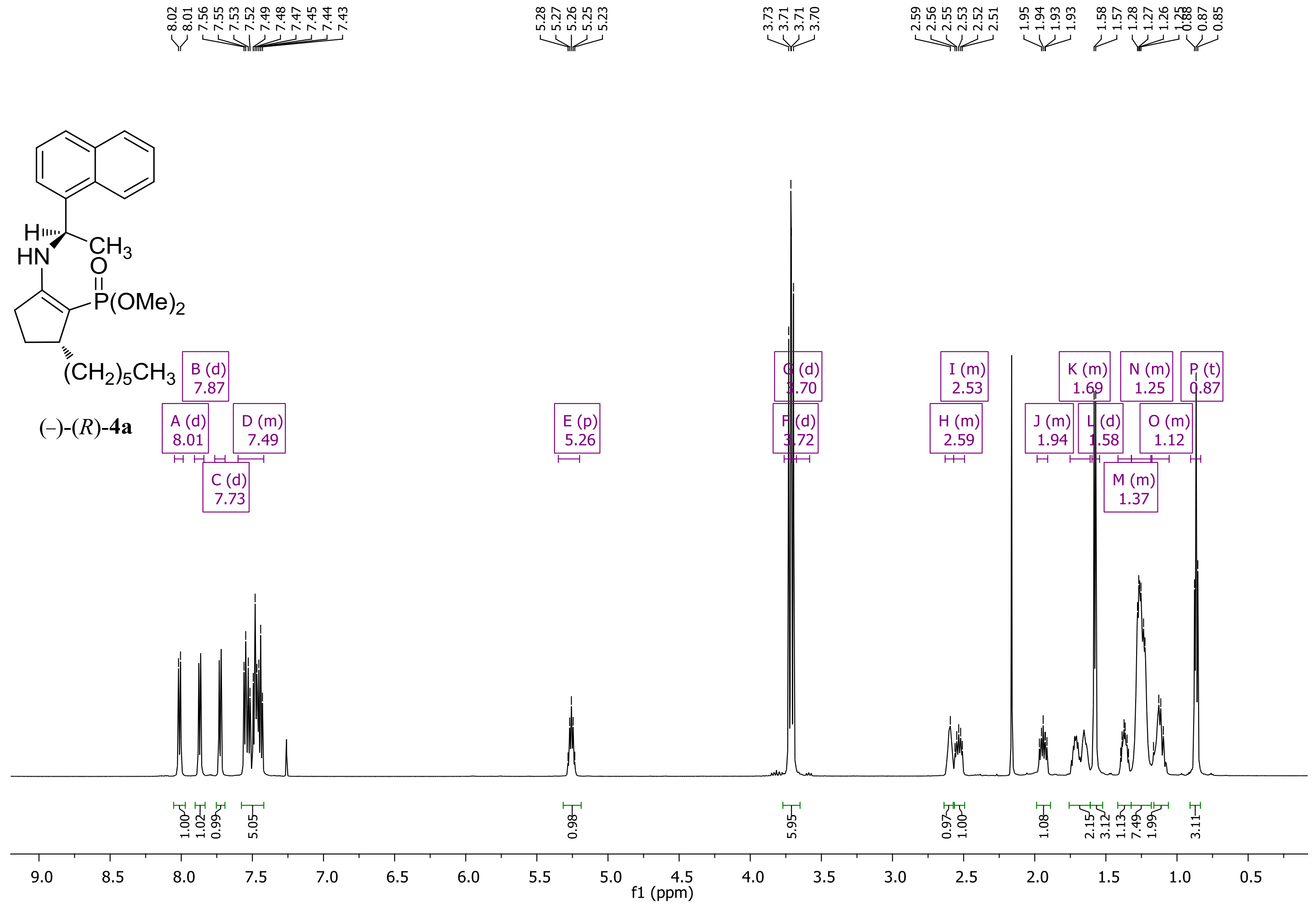

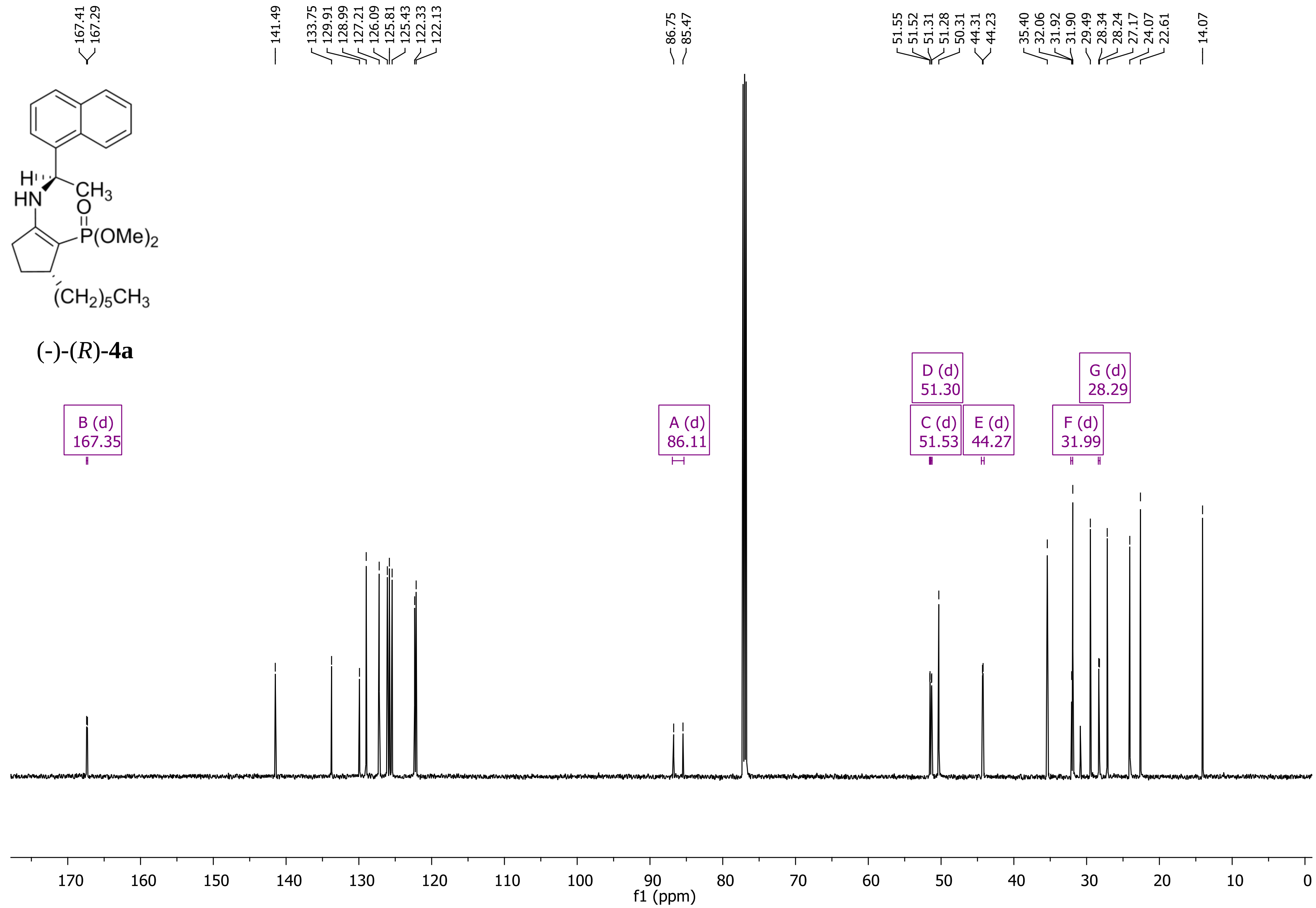
H
HN
CH3
O
P(OMe)2
(CH2)5CH3
167.41
167.29
141.49
133.75
129.91
128.99
127.21
126.09
125.81
125.43
122.33
122.13
86.75
85.47
51.55
51.52
51.31
51.28
50.31
44.31
44.23
35.40
32.06
31.92
31.90
29.49
28.34
28.24
27.17
24.07
22.61
14.07
B (d)
167.35
A (d)
86.11
D (d)
51.30
C (d)
51.53
E (d)
44.27
F (d)
31.99
G (d)
28.29
170 160 150 140 130 120 110 100 90 80 70 60 50 40 30 20 10 0
f1 (ppm)

(-)-(*R*)-**4a**

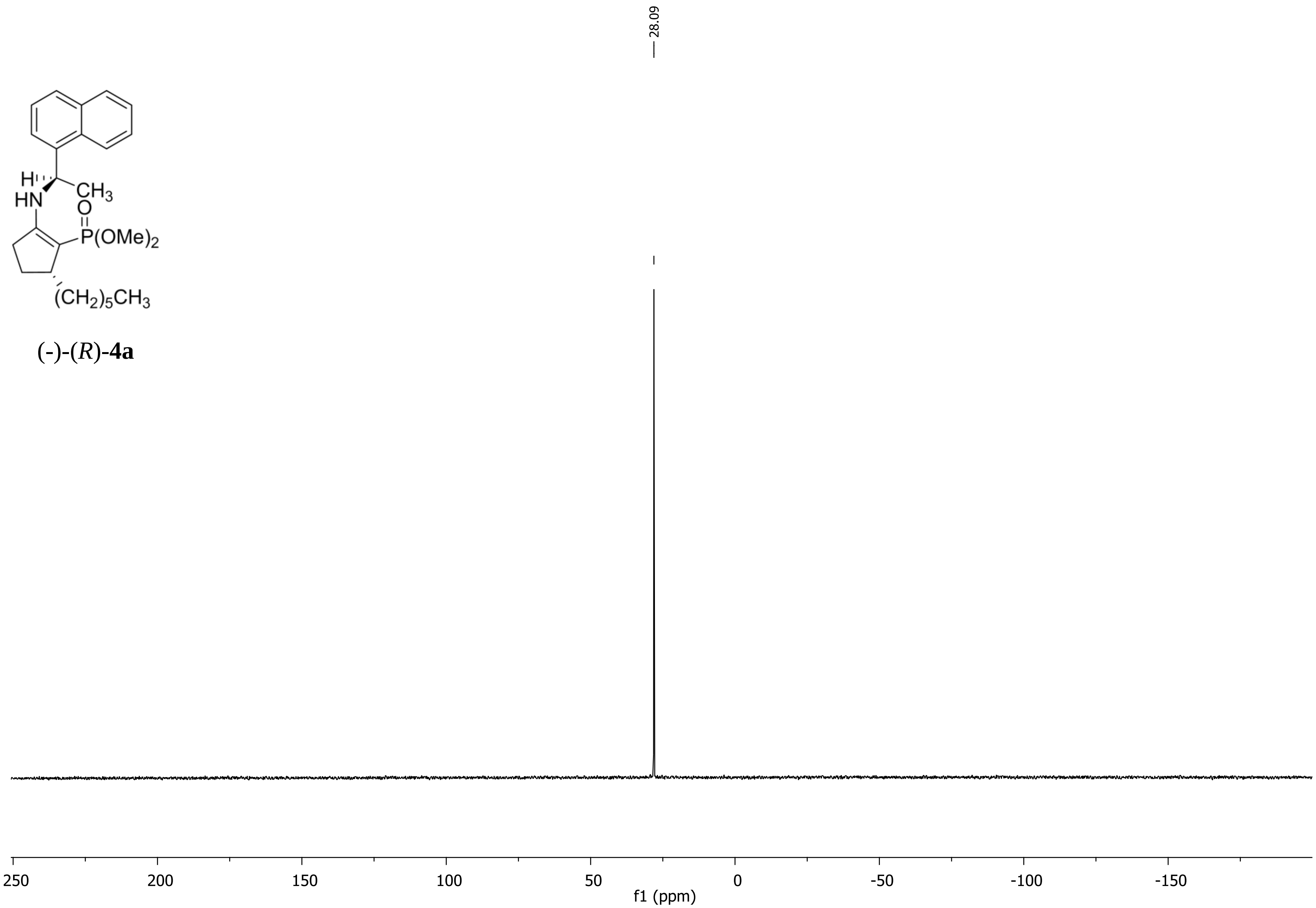
H
HN
CH3
O
P(OMe)2
(CH2)5CH3
28.09
250
200
150
100
50
0
-50
-100
-150
f1 (ppm)

(-)-(*R*)-**4a**

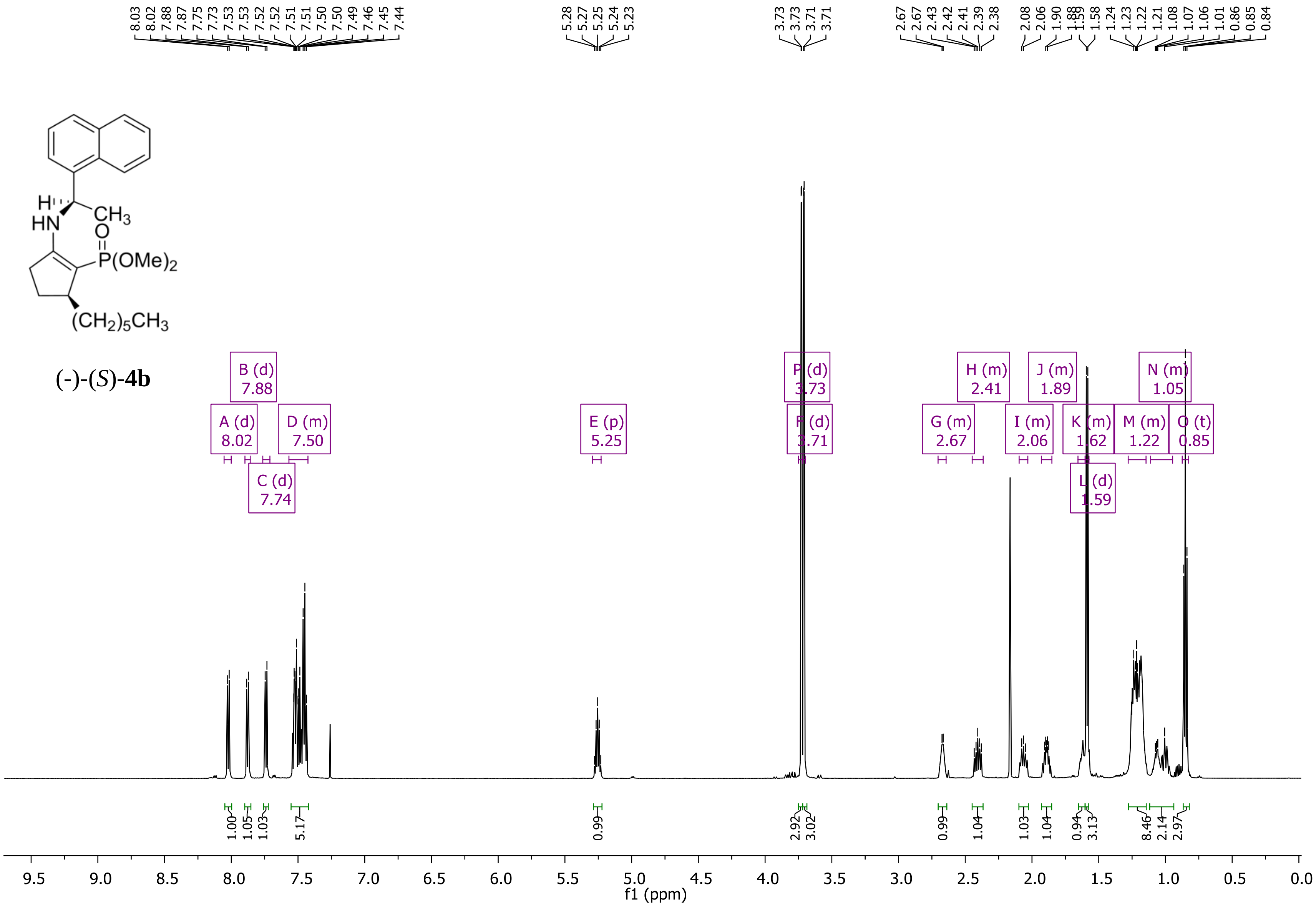

(-)-(*S*)-**4b**

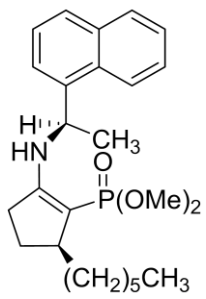

(-)-(*S*)-**4b**

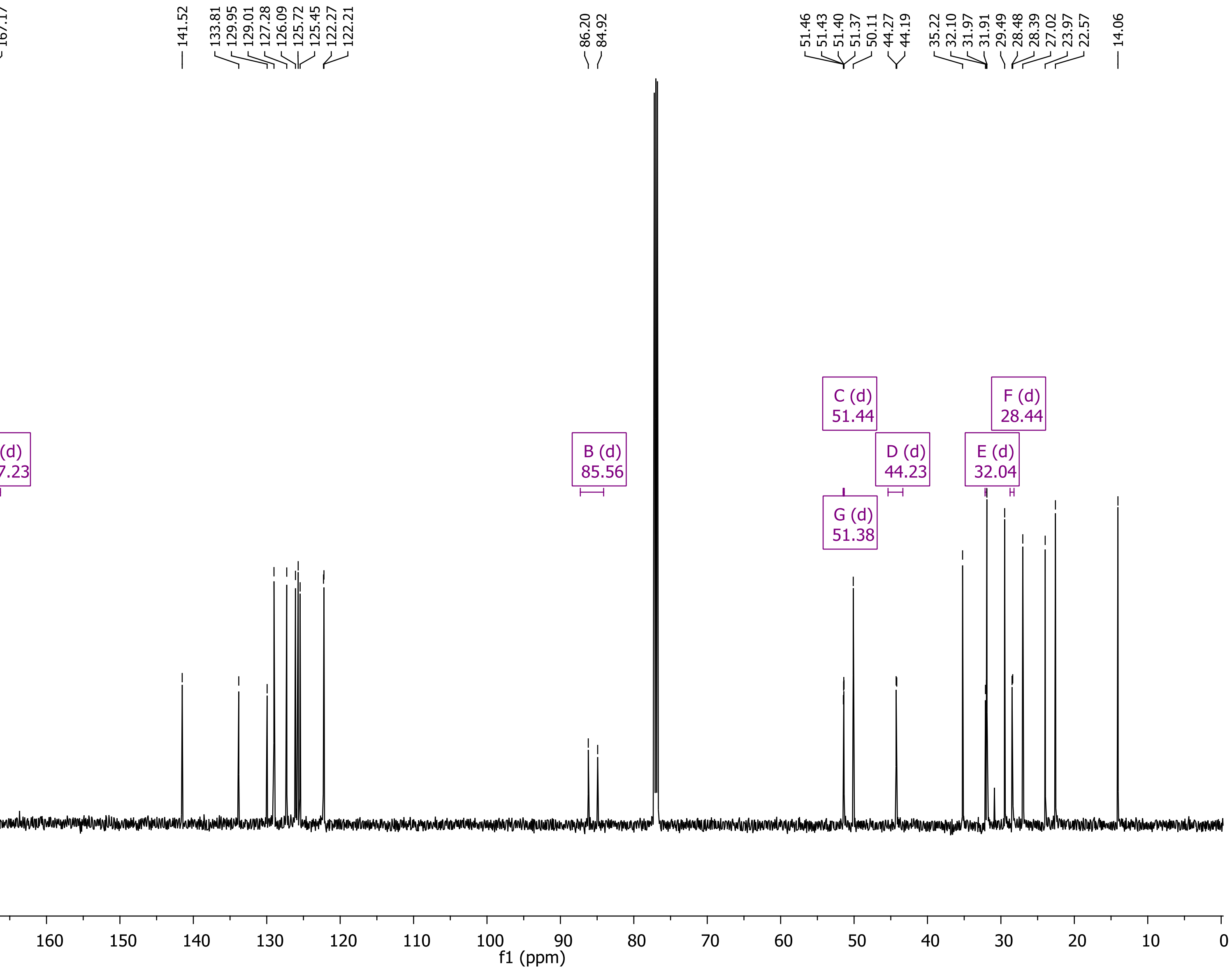

167.28
167.17
141.52
133.81
129.95
129.01
127.28
126.09
125.72
125.45
122.27
122.21
86.20
84.92
51.46
51.43
51.40
51.37
50.11
44.27
44.19
35.22
32.10
31.97
31.91
29.49
28.48
28.39
27.02
23.97
22.57
14.06
A (d) 167.23
B (d) 85.56
C (d) 51.44
D (d) 44.23
E (d) 32.04
F (d) 28.44
G (d) 51.38
180 170 160 150 140 130 120 110 100 90 80 70 60 50 40 30 20 10 0
f1 (ppm)

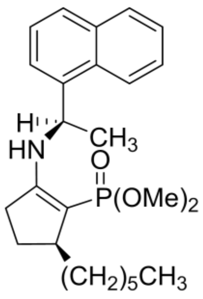
H
CH3
HN
O
P(OMe)2
(CH2)5CH3

(-)-(*S*)-**4b**

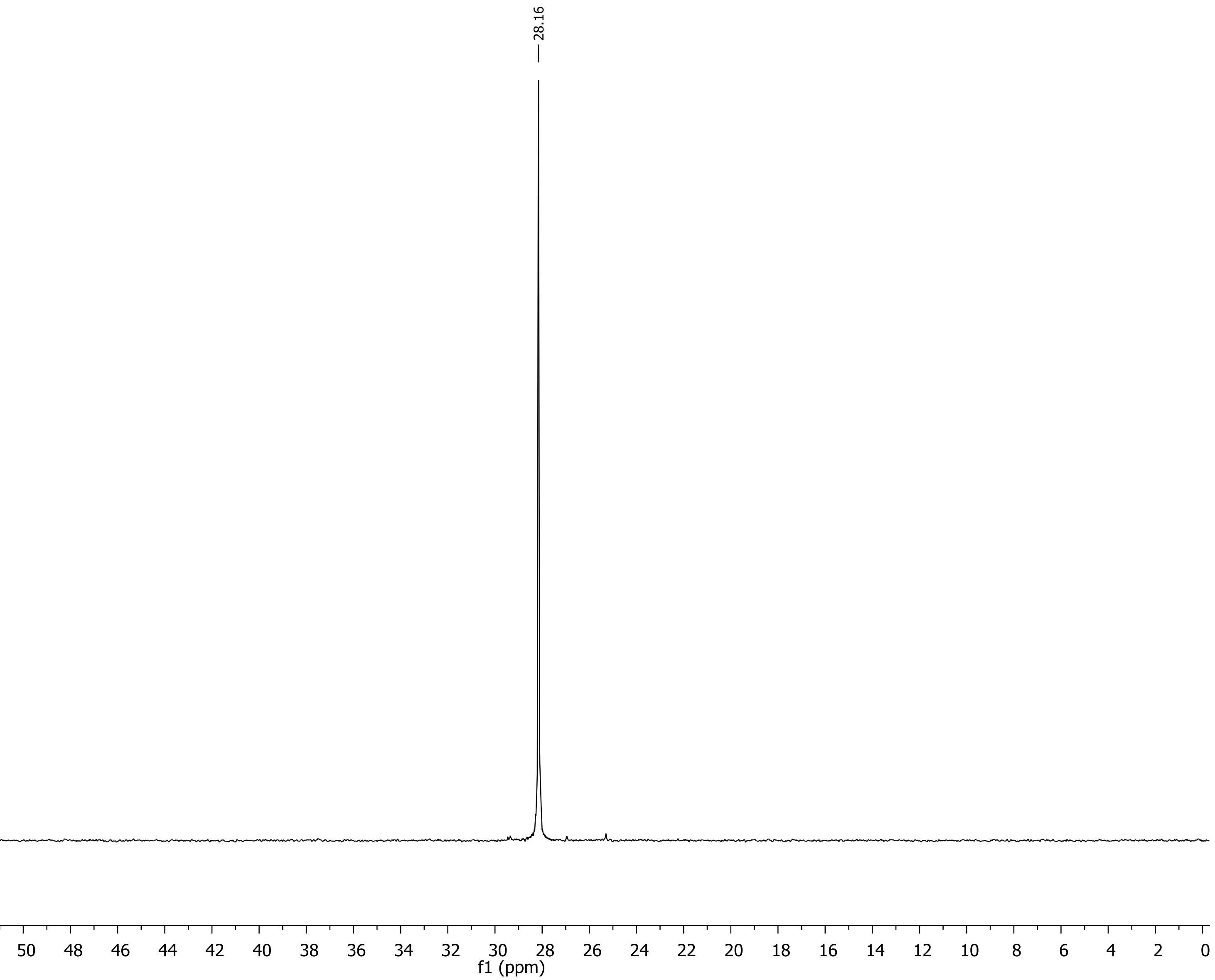
28.16
58 56 54 52 50 48 46 44 42 40 38 36 34 32 30 28 26 24 22 20 18 16 14 12 10 8 6 4 2 0
f1 (ppm)

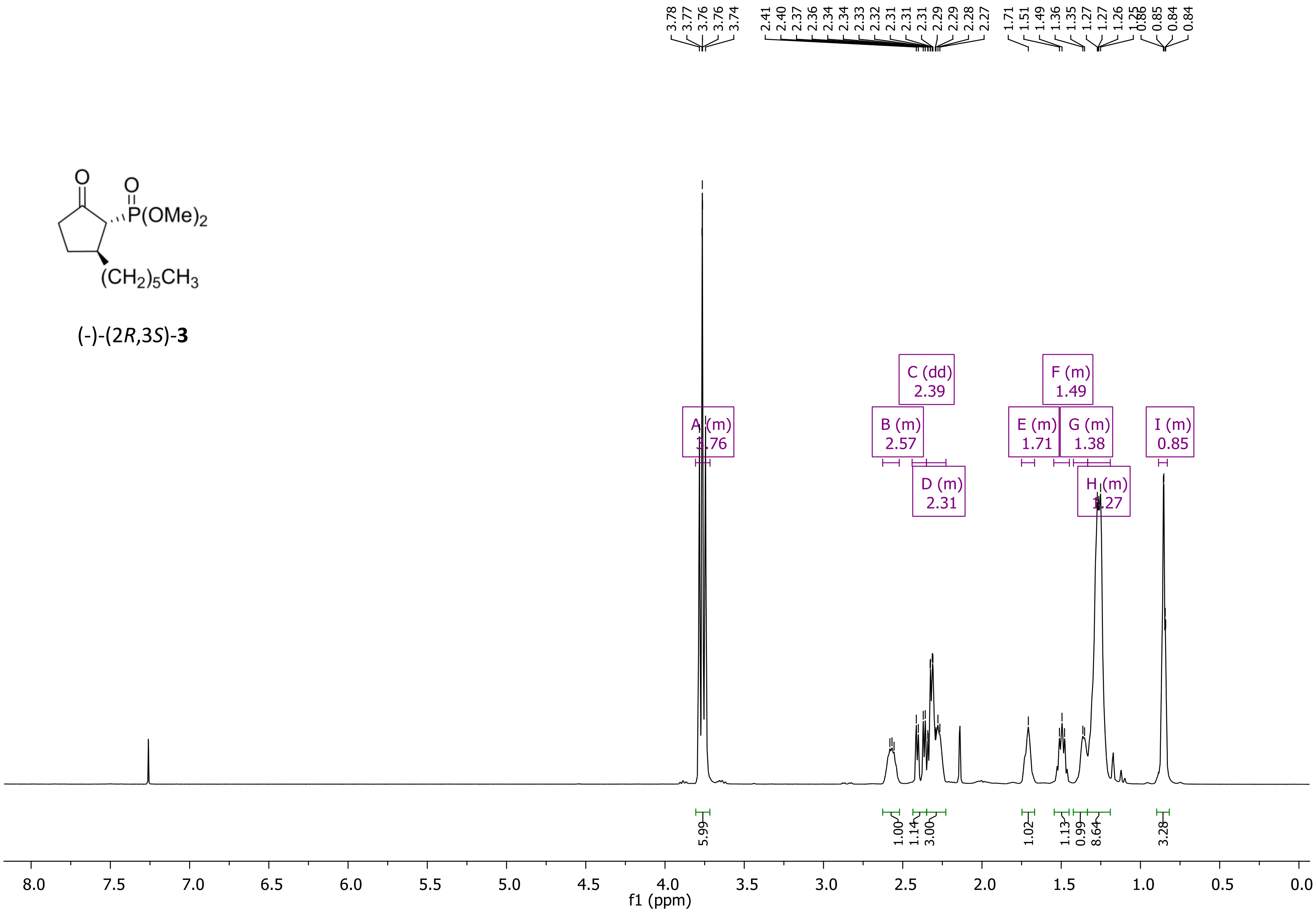

3.78 3.77 3.76 3.76 3.74
2.41 2.40 2.37 2.36 2.34 2.34 2.33 2.32 2.31 2.31 2.31 2.29 2.29 2.28 2.27
1.71 1.51 1.49 1.36 1.35 1.27 1.27 1.26 1.25 0.86 0.85 0.84 0.84
A (m) 3.76
B (m) 2.57
C (dd) 2.39
D (m) 2.31
E (m) 1.71
F (m) 1.49
G (m) 1.38
H (m) 1.27
I (m) 0.85
5.99
1.00 1.14 3.00
1.02
1.13 0.99 8.64
3.28
8.0 7.5 7.0 6.5 6.0 5.5 5.0 4.5 4.0 3.5 3.0 2.5 2.0 1.5 1.0 0.5 0.0
f1 (ppm)

(-)-(2*R*,3*S*)-**3**

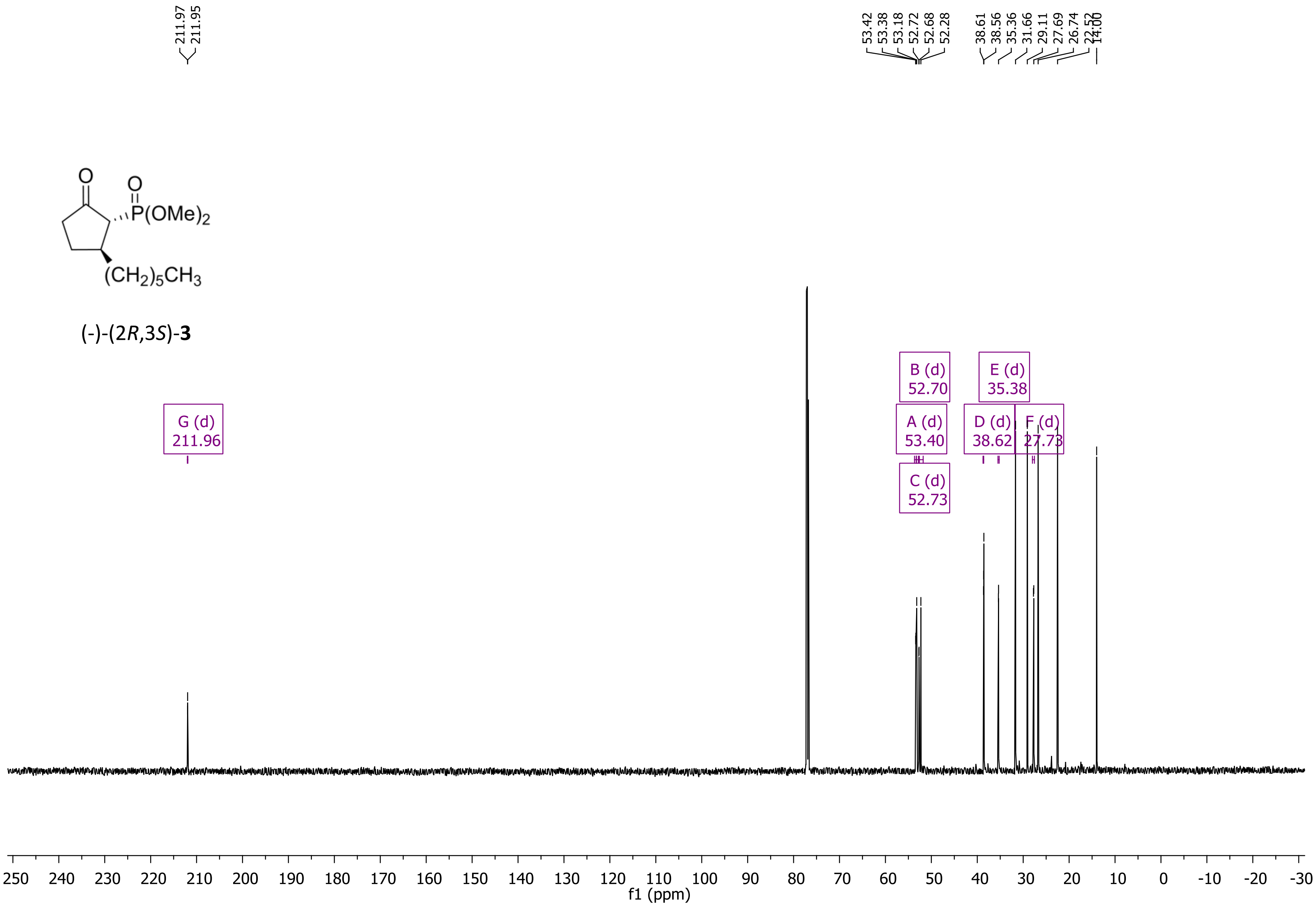
211.97
211.95
53.42
53.38
53.18
52.72
52.68
52.28
38.61
38.56
35.36
31.66
29.11
27.69
26.74
22.52
14.00
G (d) 211.96
B (d) 52.70
A (d) 53.40
C (d) 52.73
E (d) 35.38
D (d) 38.62
F (d) 27.73
250 240 230 220 210 200 190 180 170 160 150 140 130 120 110 100 90 80 70 60 50 40 30 20 10 0 -10 -20 -30
f1 (ppm)

(-)-(2*R*,3*S*)-**3**

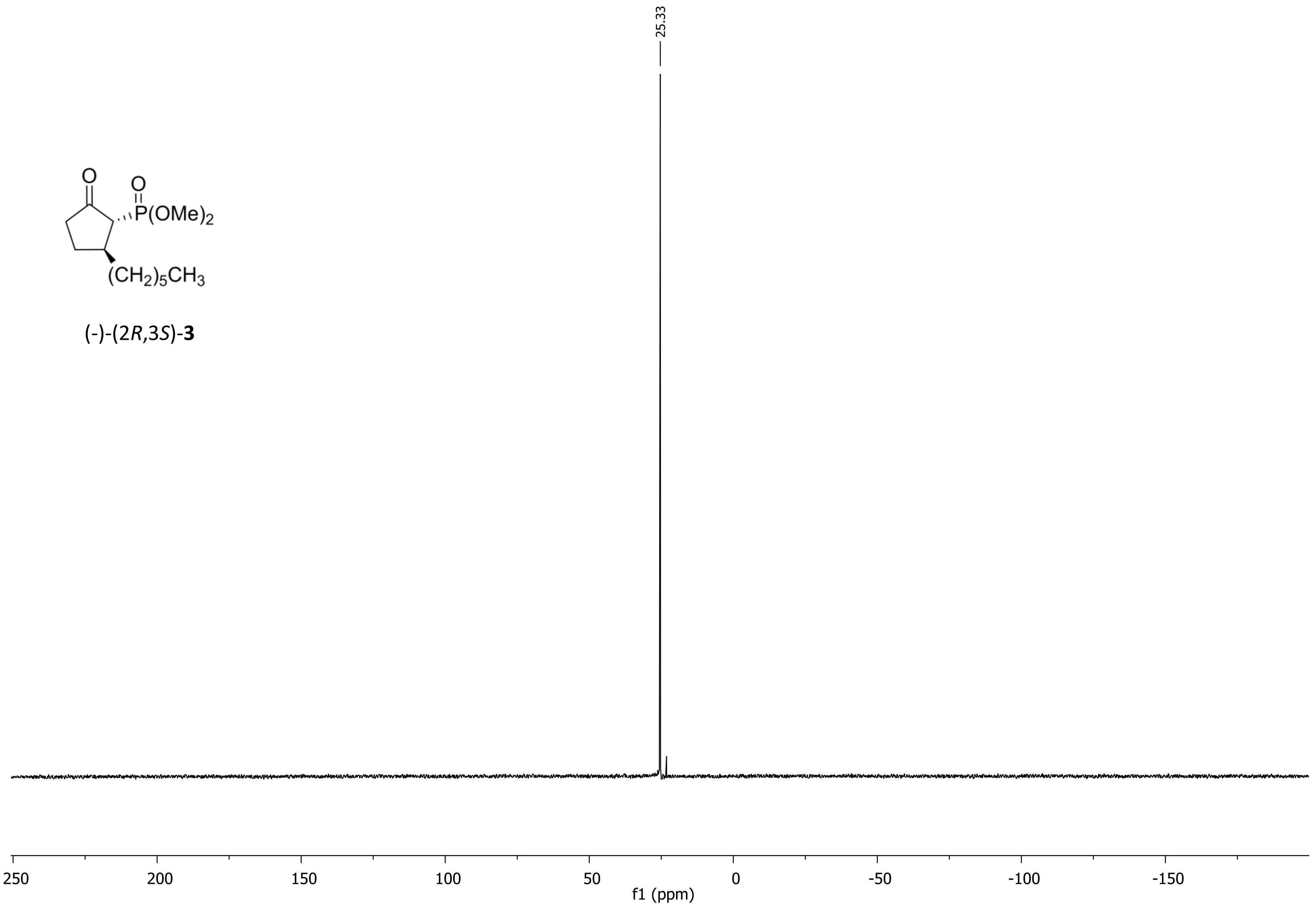

(-)-(2*R*,3*S*)-**3**

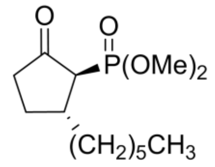

(+)-(2*S*,3*R*)-**3**

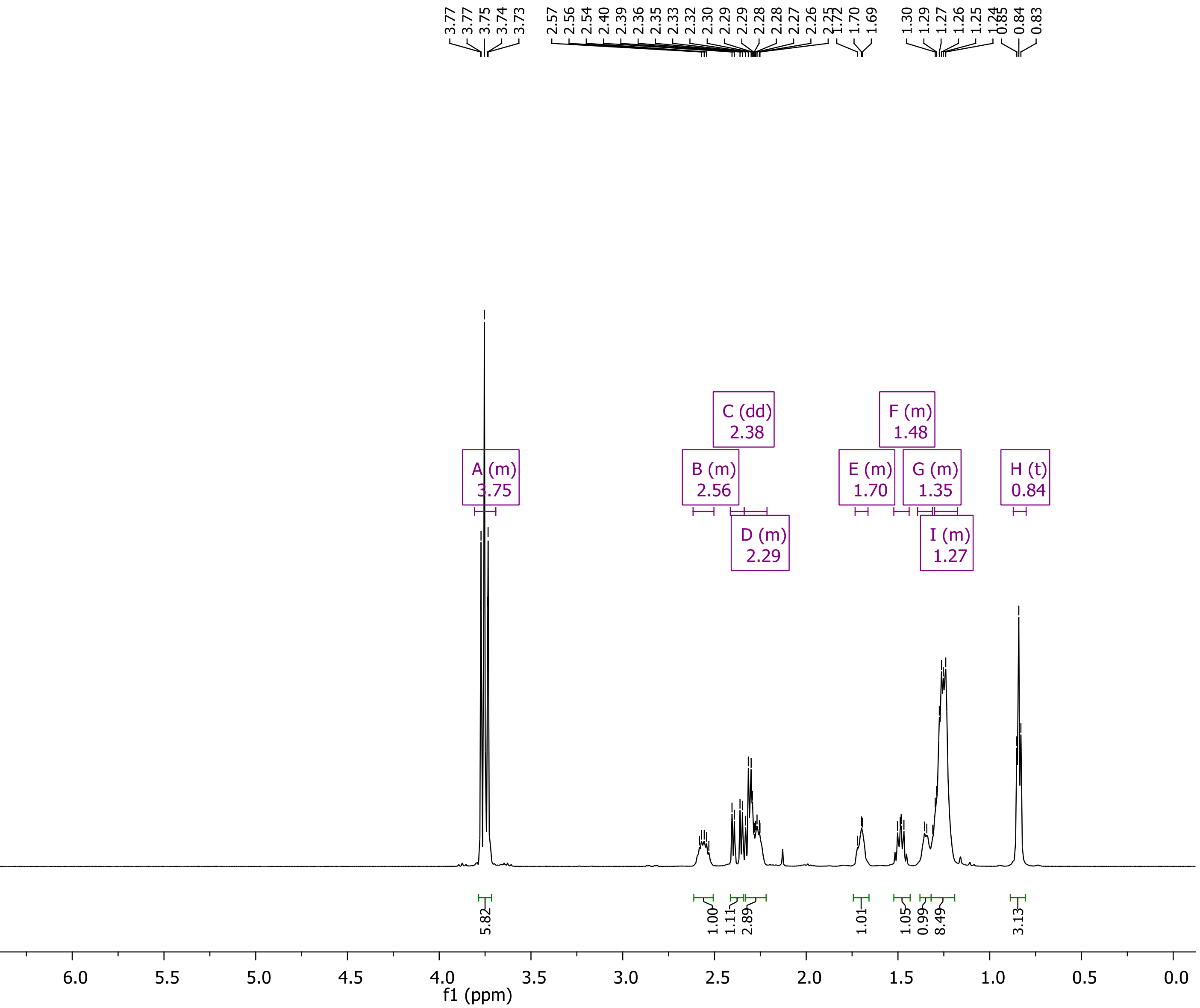

3.77 3.77 3.75 3.74 3.73
2.57 2.56 2.54 2.40 2.39 2.36 2.35 2.33 2.32 2.30 2.29 2.29 2.28 2.28 2.27 2.26 2.25 1.72 1.70 1.69
1.30 1.29 1.27 1.26 1.25 1.24 0.85 0.84 0.83
A (m) 3.75
B (m) 2.56
C (dd) 2.38
D (m) 2.29
E (m) 1.70
F (m) 1.48
G (m) 1.35
I (m) 1.27
H (t) 0.84
5.82
1.00
1.11
2.89
1.01
1.05
0.99
8.49
3.13
7.5 7.0 6.5 6.0 5.5 5.0 4.5 4.0 3.5 3.0 2.5 2.0 1.5 1.0 0.5 0.0
f1 (ppm)

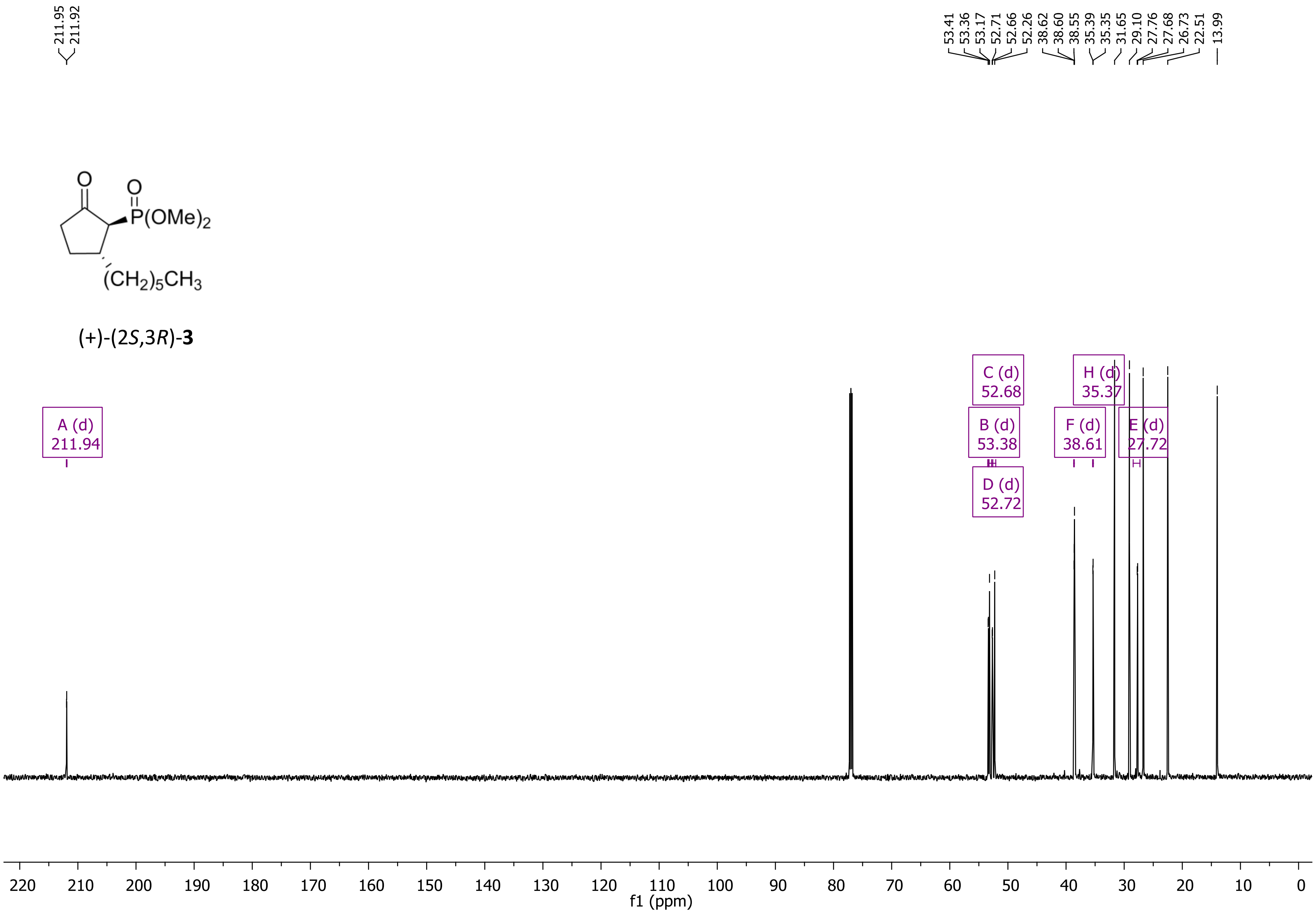

211.95
211.92
53.41
53.36
53.17
52.71
52.66
52.26
38.62
38.60
38.55
35.39
35.35
31.65
29.10
27.76
27.68
26.73
22.51
13.99
P(OMe)2
(CH2)5CH3
(+)-(2S,3R)-3
A (d) 211.94
B (d) 53.38
C (d) 52.68
D (d) 52.72
F (d) 38.61
H (d) 35.37
E (d) 27.72
220
210
200
190
180
170
160
150
140
130
120
110
100
90
80
70
60
50
40
30
20
10
0
f1 (ppm)

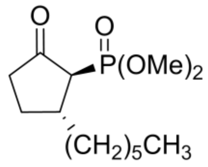

O
O
P(OMe)2
(CH2)5CH3

(+)-(2*S*,3*R*)-**3**

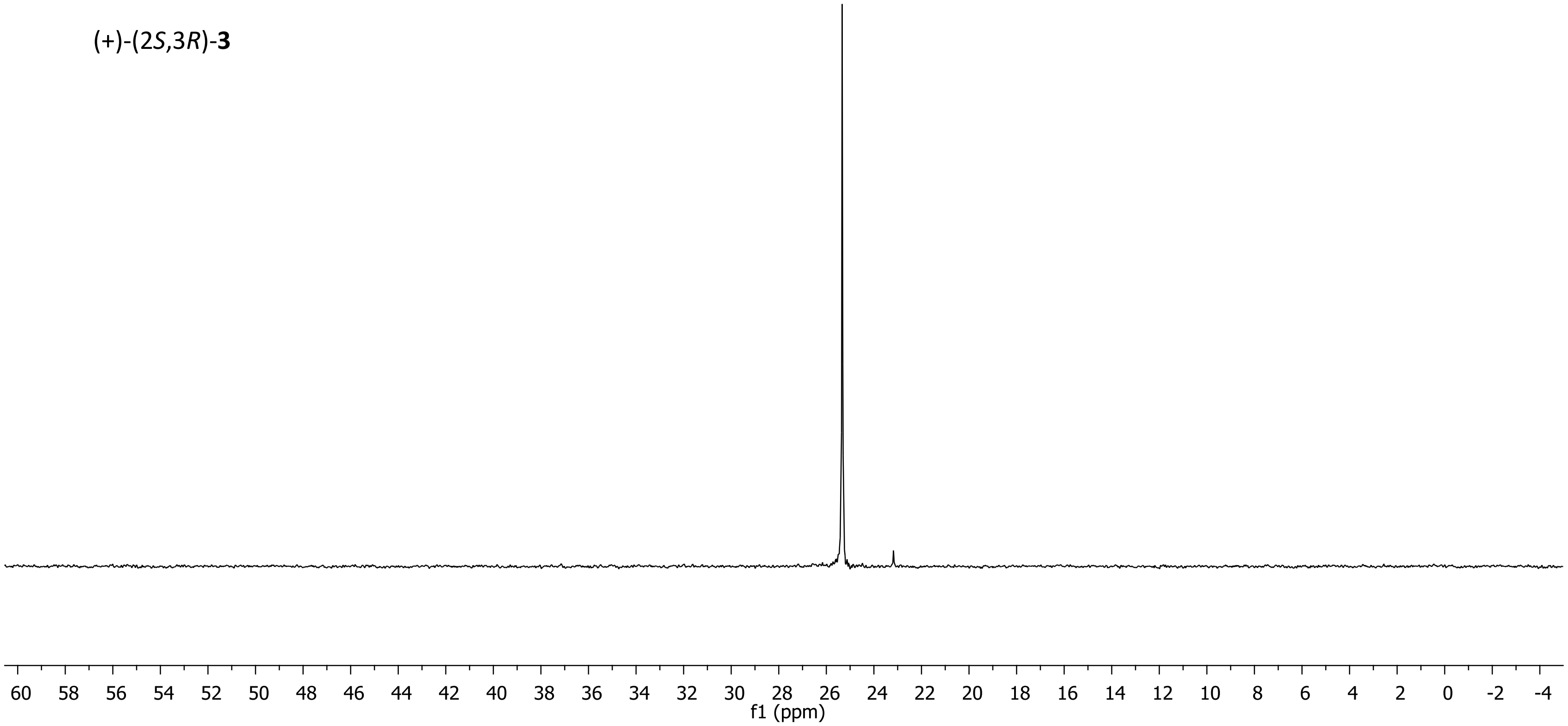

25.33
60 58 56 54 52 50 48 46 44 42 40 38 36 34 32 30 28 26 24 22 20 18 16 14 12 10 8 6 4 2 0 -2 -4
f1 (ppm)

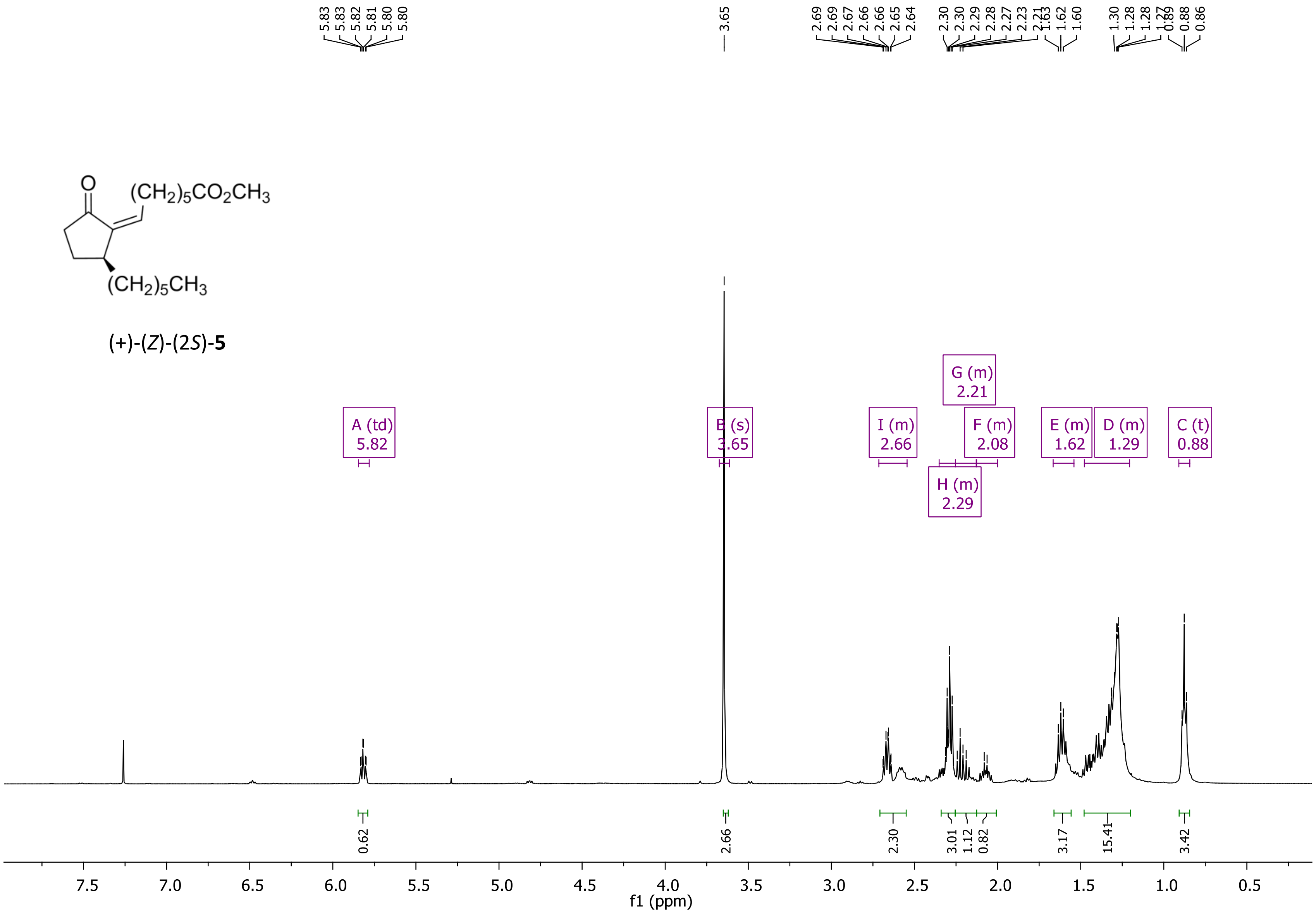
O
(CH2)5CO2CH3
(CH2)5CH3
5.83
5.83
5.82
5.81
5.80
5.80
3.65
2.69
2.69
2.67
2.66
2.66
2.65
2.64
2.30
2.30
2.29
2.28
2.27
2.23
2.21
1.63
1.62
1.60
1.30
1.28
1.28
1.27
0.89
0.88
0.86
A (td) 5.82
B (s) 3.65
I (m) 2.66
G (m) 2.21
H (m) 2.29
F (m) 2.08
E (m) 1.62
D (m) 1.29
C (t) 0.88
0.62
2.66
2.30
3.01
1.12
0.82
3.17
15.41
3.42
7.5
7.0
6.5
6.0
5.5
5.0
4.5
4.0
3.5
3.0
2.5
2.0
1.5
1.0
0.5
f1 (ppm)

(+)-(*Z*)-(2*S*)-**5**

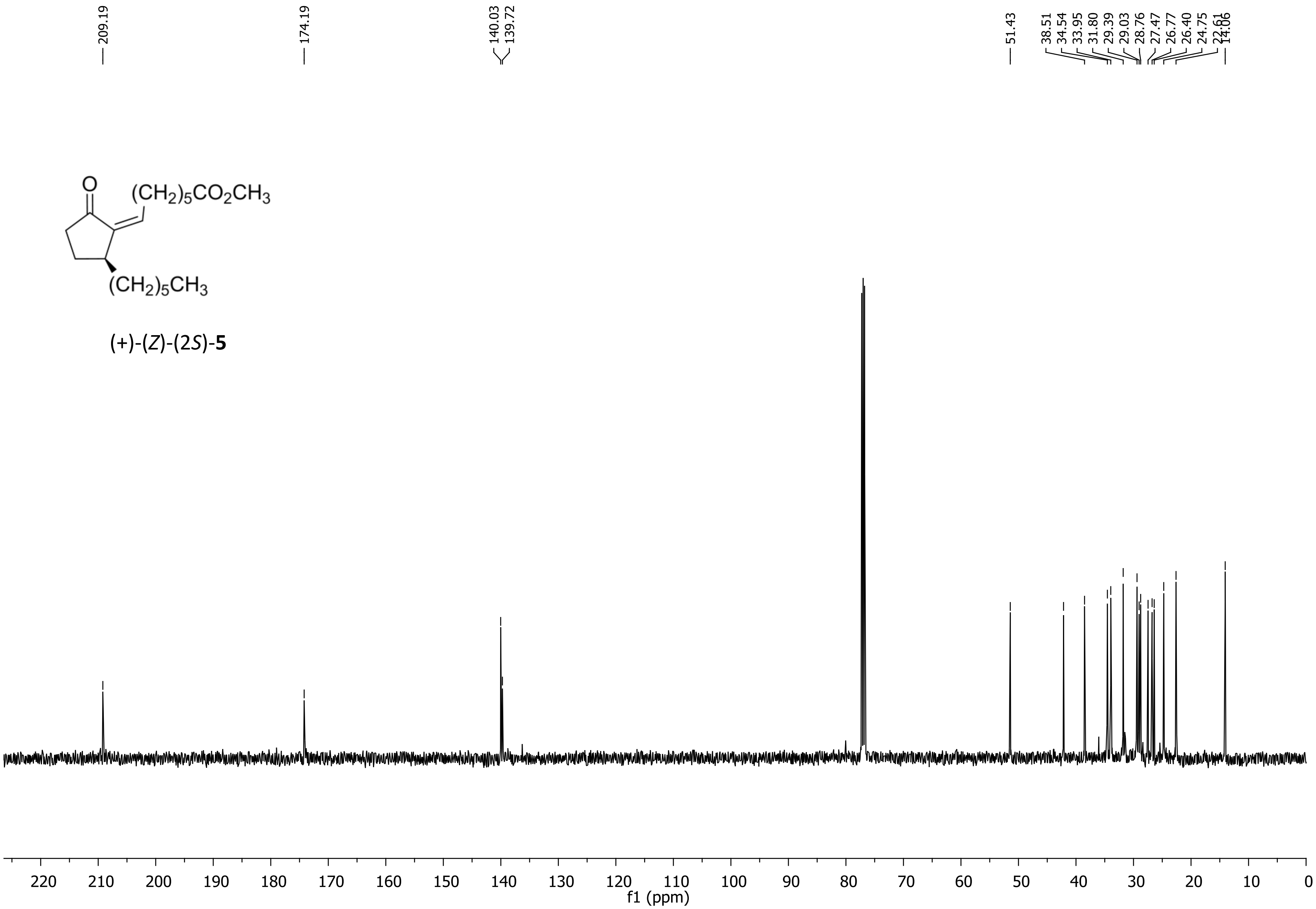
209.19
174.19
140.03
139.72
51.43
38.51
34.54
33.95
31.80
29.39
29.03
28.76
27.47
26.77
26.40
24.75
22.61
14.06
O
(CH2)5CO2CH3
(CH2)5CH3
220 210 200 190 180 170 160 150 140 130 120 110 100 90 80 70 60 50 40 30 20 10 0
f1 (ppm)

(+)-(*Z*)-(2*S*)-**5**

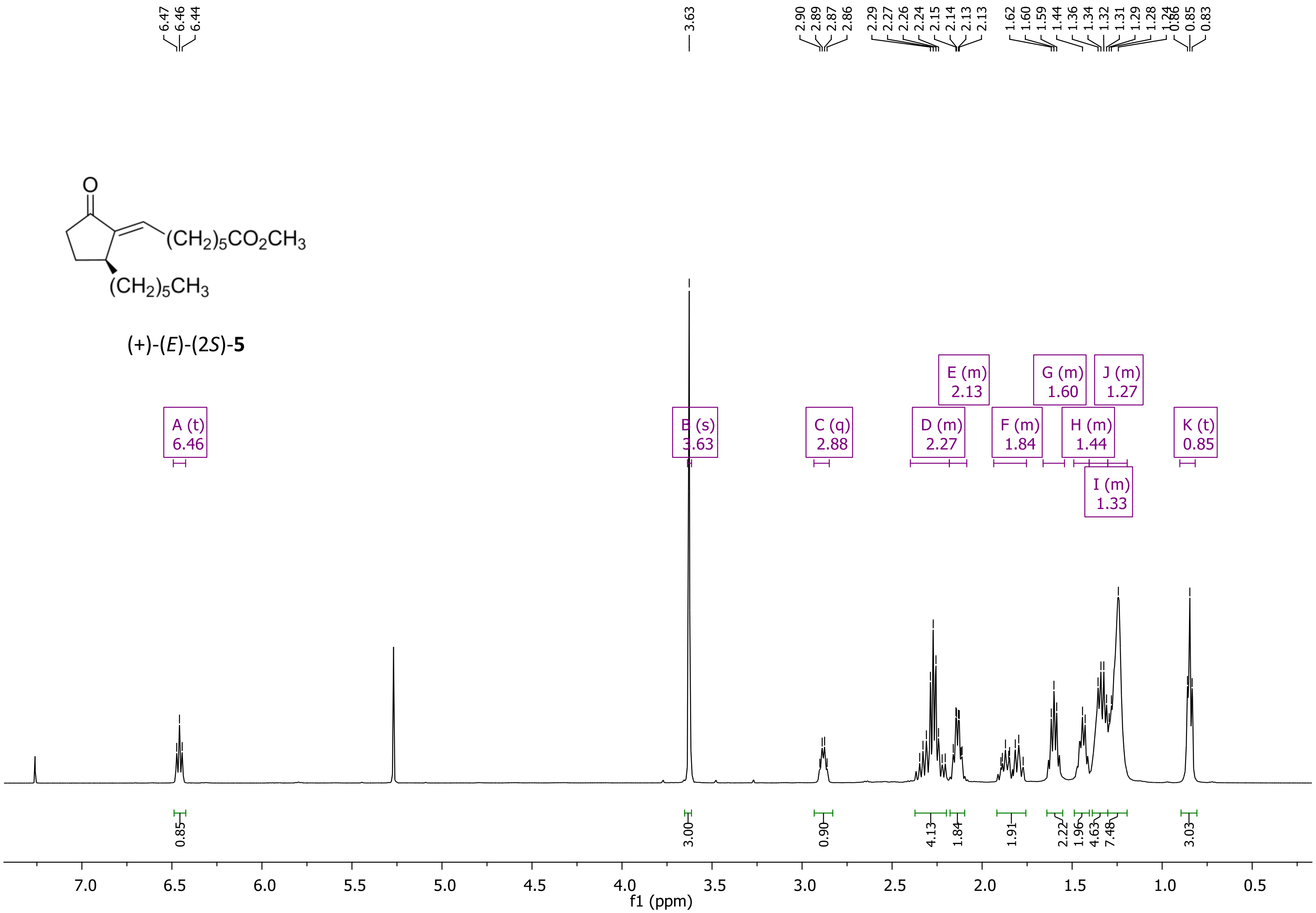

6.47
6.46
6.44
3.63
2.90
2.89
2.87
2.86
2.29
2.27
2.26
2.24
2.15
2.14
2.13
2.13
1.62
1.60
1.59
1.44
1.36
1.34
1.32
1.31
1.29
1.28
1.24
0.86
0.85
0.83
A (t)
6.46
B (s)
3.63
C (q)
2.88
D (m)
2.27
E (m)
2.13
F (m)
1.84
G (m)
1.60
H (m)
1.44
I (m)
1.33
J (m)
1.27
K (t)
0.85
0.85
3.00
0.90
4.13
1.84
1.91
2.22
1.96
4.63
7.48
3.03
7.0
6.5
6.0
5.5
5.0
4.5
4.0
3.5
3.0
2.5
2.0
1.5
1.0
0.5
f1 (ppm)

(+)-(*E*)-(2*S*)-**5**

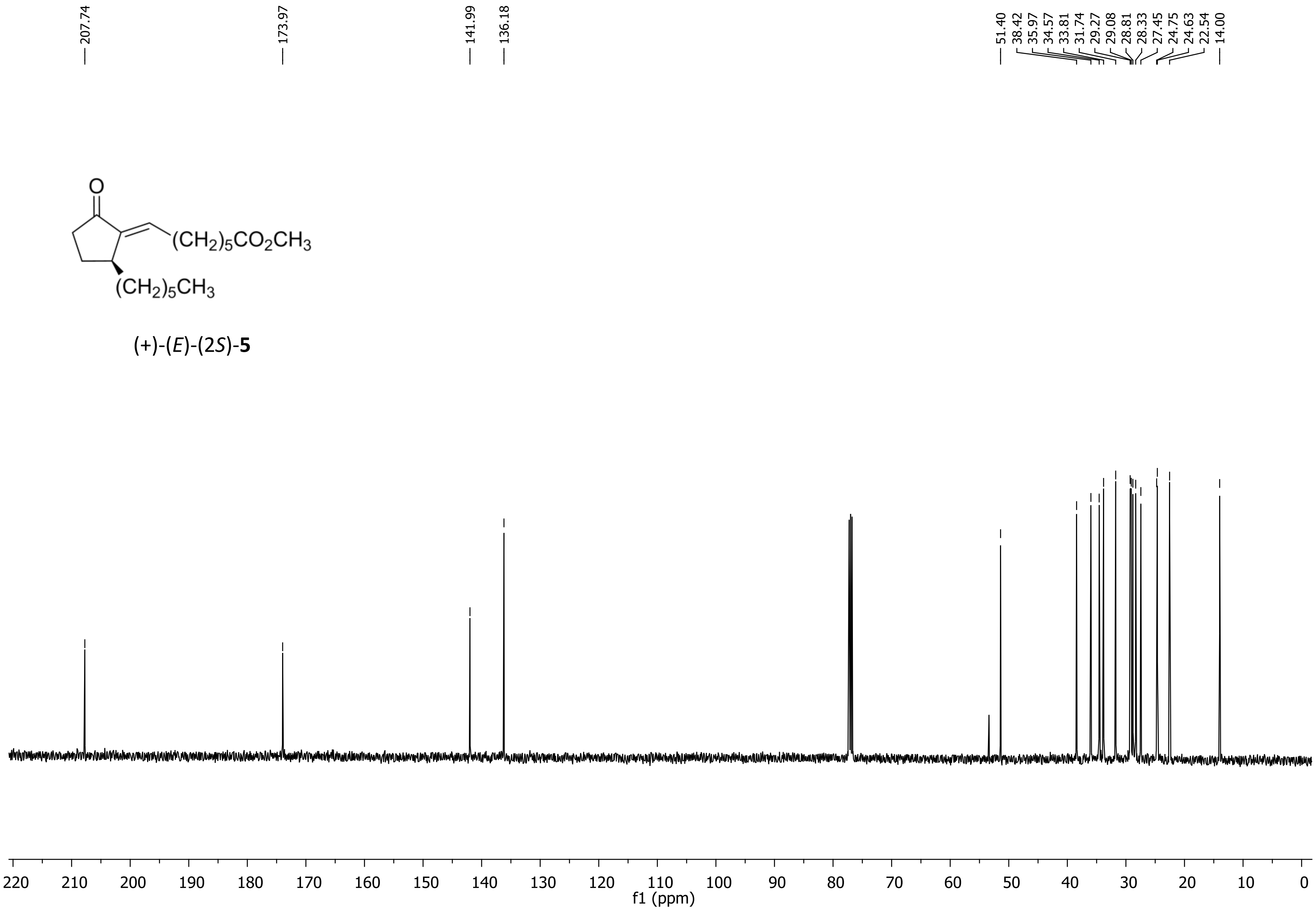

207.74
173.97
141.99
136.18
51.40
38.42
35.97
34.57
33.81
31.74
29.27
29.08
28.81
28.33
27.45
24.75
24.63
22.54
14.00
O
(CH2)5CO2CH3
(CH2)5CH3
(+)-(E)-(2S)-5
220 210 200 190 180 170 160 150 140 130 120 110 100 90 80 70 60 50 40 30 20 10 0
f1 (ppm)

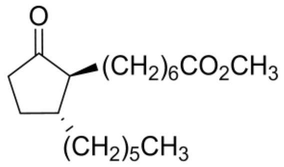
O
(CH2)6CO2CH3
(CH2)5CH3

(+)-(1*S*,2*R*)-**6**

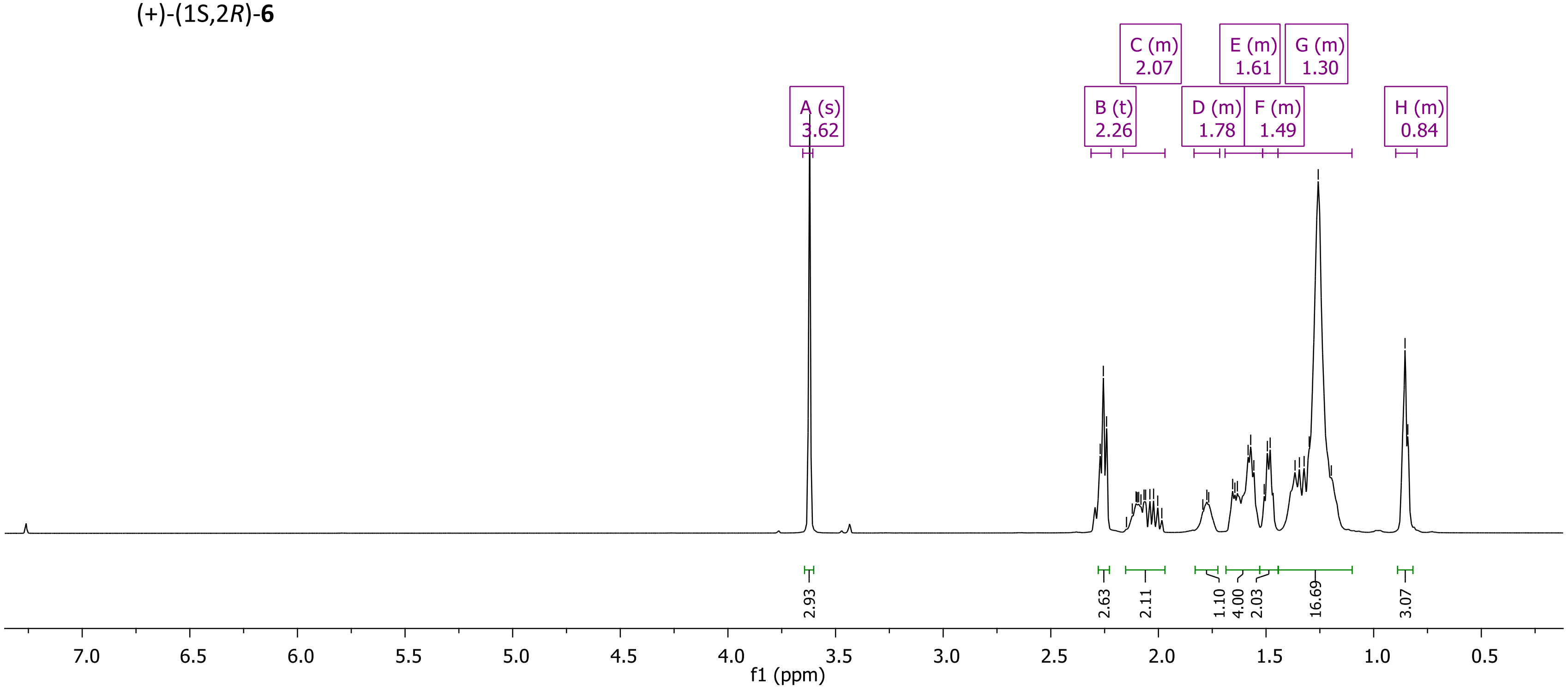
3.62
2.27
2.26
2.24
2.15
2.12
2.10
2.10
2.09
2.08
2.07
2.06
2.04
2.02
2.00
1.98
1.58
1.57
1.56
1.49
1.48
1.37
1.35
1.32
1.30
1.26
0.85
0.84
A (s) 3.62
B (t) 2.26
C (m) 2.07
D (m) 1.78
E (m) 1.61
F (m) 1.49
G (m) 1.30
H (m) 0.84
2.93
2.63
2.11
1.10
4.00
2.03
16.69
3.07
7.0
6.5
6.0
5.5
5.0
4.5
4.0
3.5
3.0
2.5
2.0
1.5
1.0
0.5
f1 (ppm)

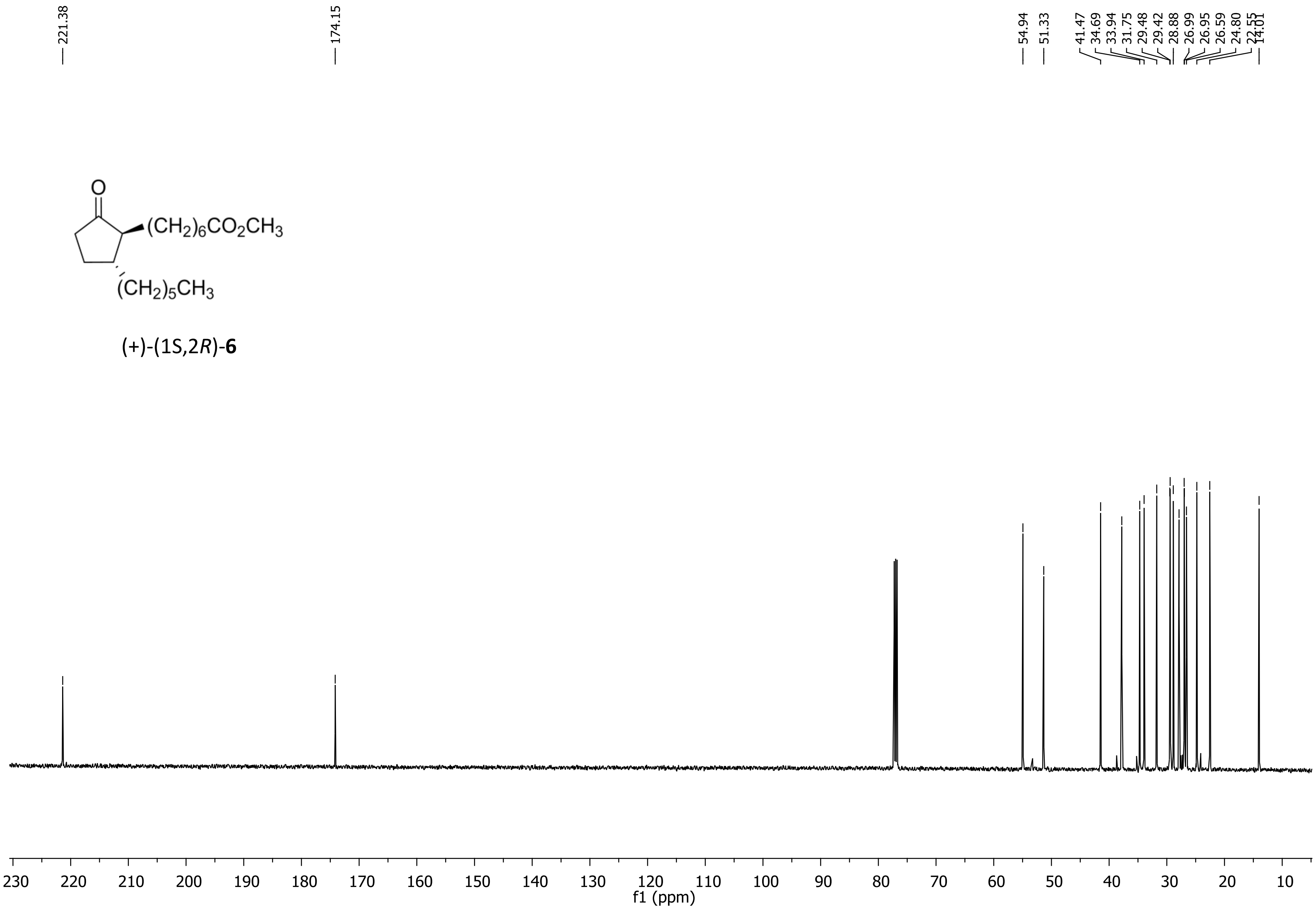

221.38
174.15
54.94
51.33
41.47
34.69
33.94
31.75
29.48
29.42
28.88
26.99
26.95
26.59
24.80
22.55
14.01
O
$(CH_2)_6CO_2CH_3$
$(CH_2)_5CH_3$
230 220 210 200 190 180 170 160 150 140 130 120 110 100 90 80 70 60 50 40 30 20 10
f1 (ppm)

(+)-(1*S*,2*R*)-**6**

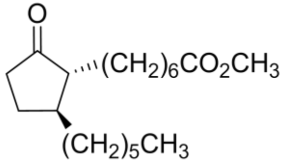

(-)-(1R,2*S*)-**6**

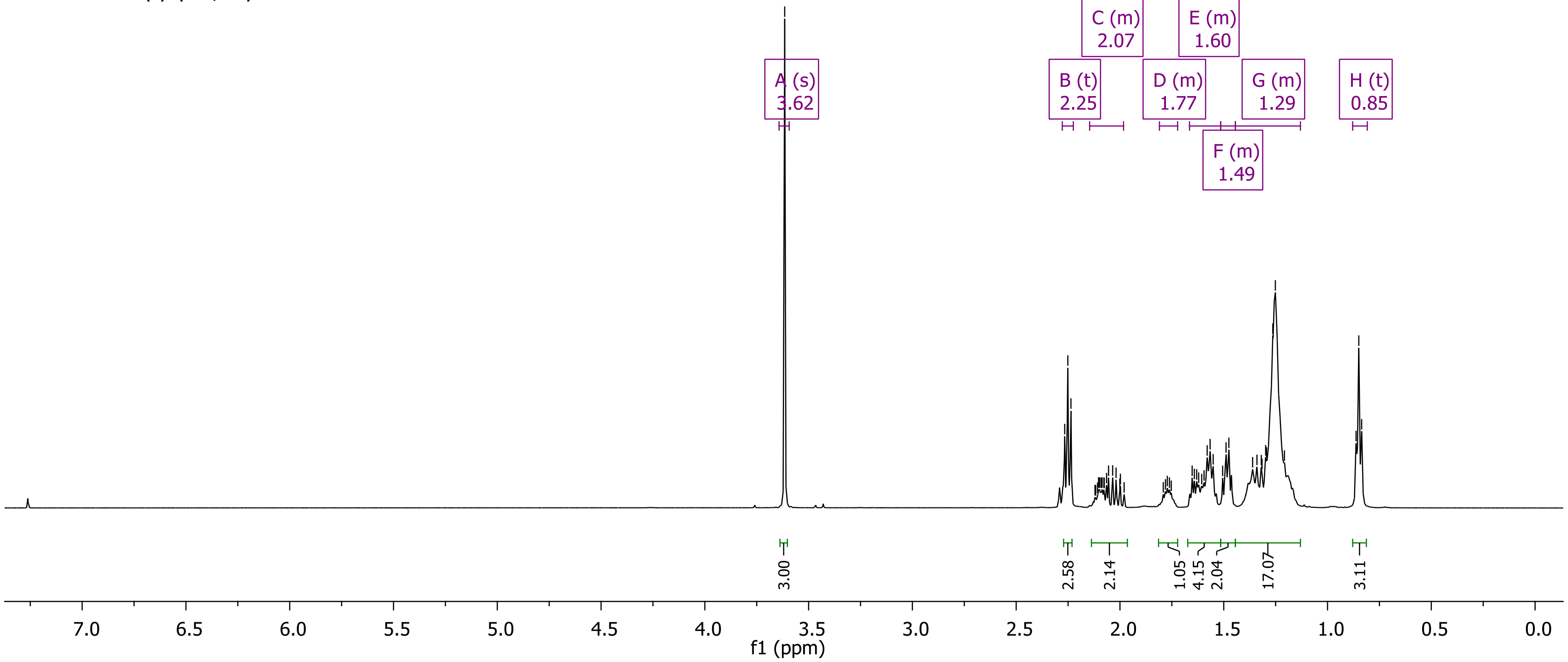

3.62
2.27
2.25
2.24
2.12
2.11
2.10
2.10
2.09
2.08
2.08
2.06
2.05
2.04
2.02
2.00
2.00
1.98
1.58
1.57
1.55
1.49
1.48
1.30
1.29
1.26
1.25
1.21
0.86
0.85
0.84
A (s)
3.62
B (t)
2.25
C (m)
2.07
D (m)
1.77
E (m)
1.60
F (m)
1.49
G (m)
1.29
H (t)
0.85
3.00
2.58
2.14
1.05
4.15
2.04
17.07
3.11
7.0
6.5
6.0
5.5
5.0
4.5
4.0
3.5
3.0
2.5
2.0
1.5
1.0
0.5
0.0
f1 (ppm)

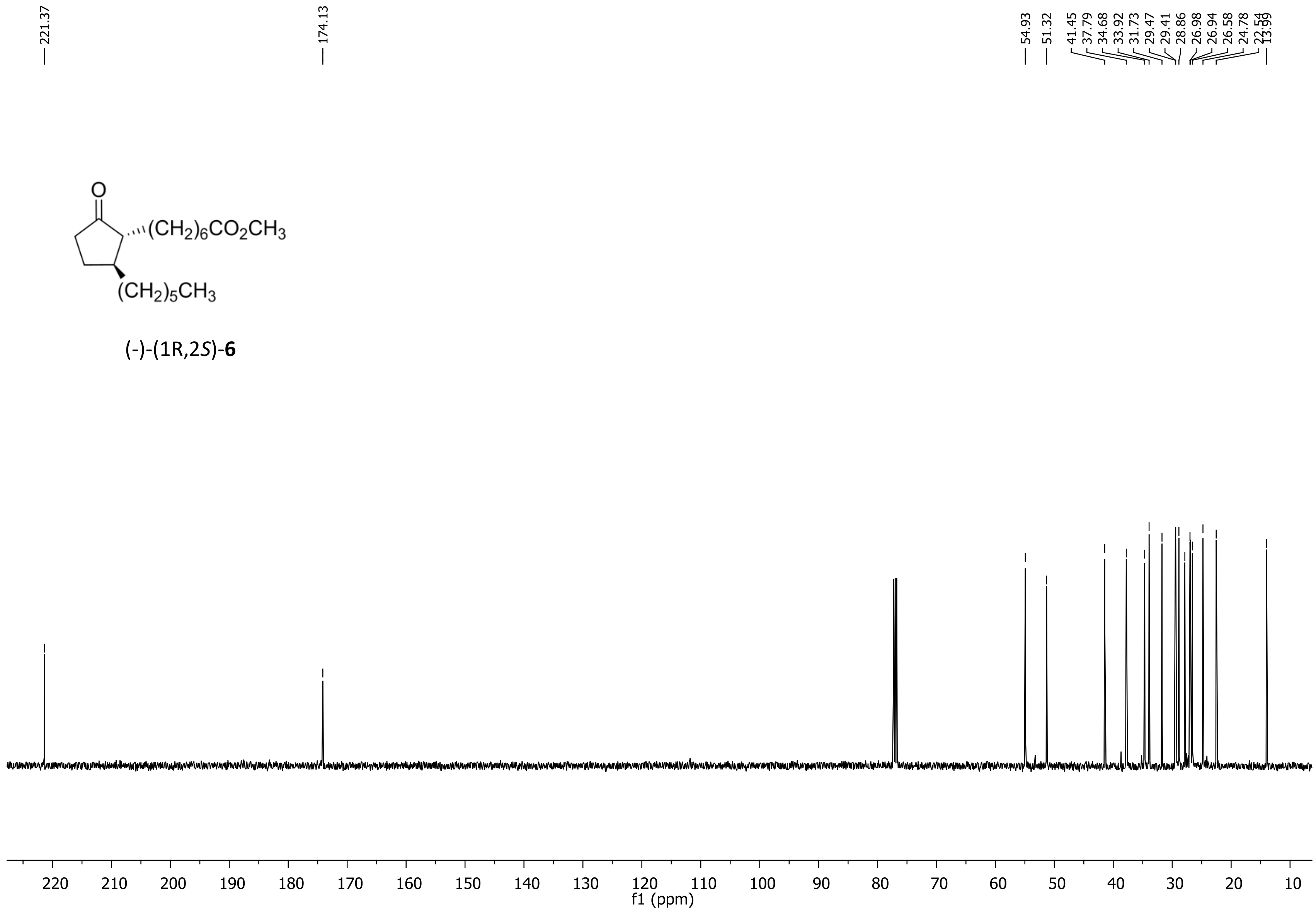

221.37
174.13
54.93
51.32
41.45
37.79
34.68
33.92
31.73
29.47
29.41
28.86
26.98
26.94
26.58
24.78
22.54
13.99
O
(CH2)6CO2CH3
(CH2)5CH3
220
210
200
190
180
170
160
150
140
130
120
110
100
90
80
70
60
50
40
30
20
10
f1 (ppm)

(-)-(1R,2*S*)-**6**

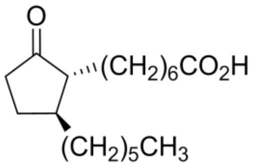
O
(CH2)6CO2H
(CH2)5CH3

(-)-(1R,2*S*)-**7**

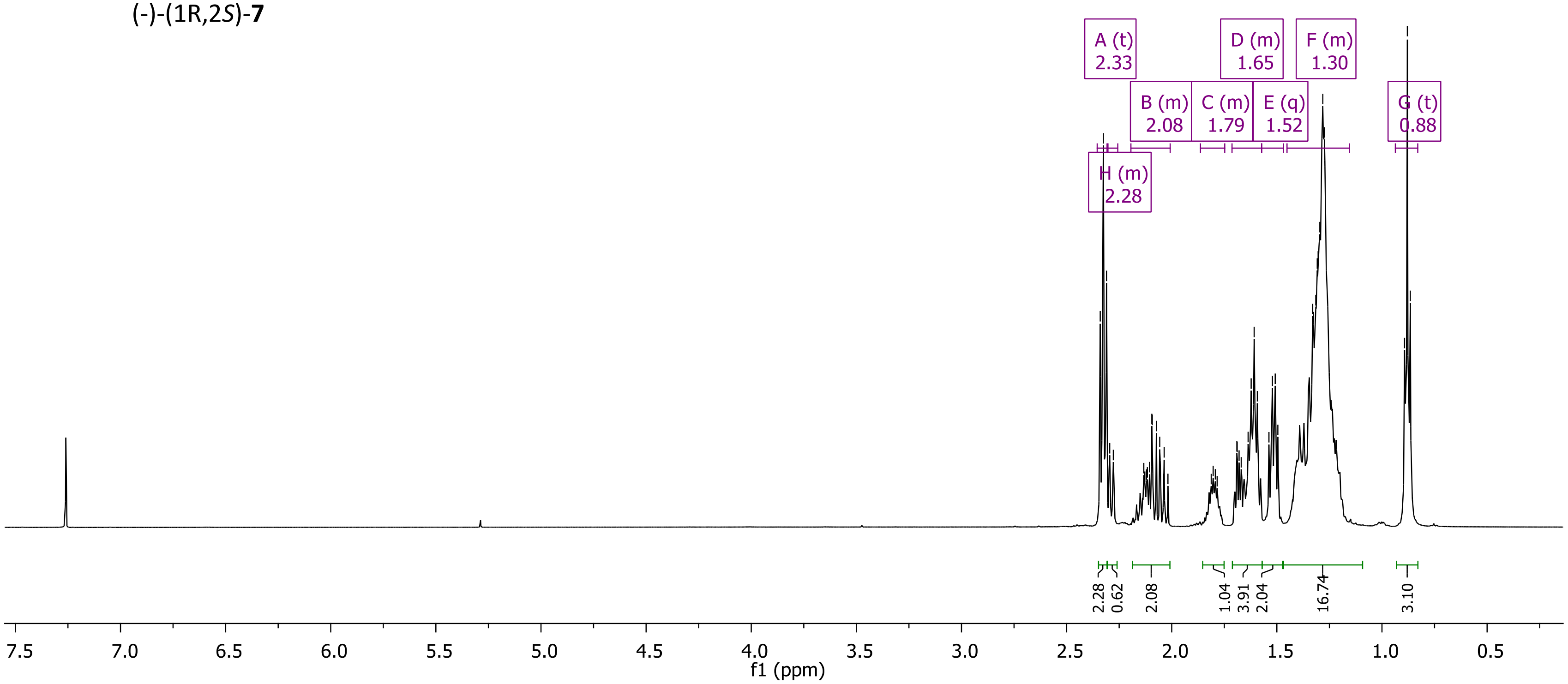
2.34 2.33 2.31 2.30 2.28 2.13 2.13 2.12 2.12 2.11 2.09 2.09 2.07 2.06 2.04 2.04 2.02 1.61 1.51 1.33 1.33 1.31 1.31 1.30 1.30 1.28 1.27 0.89 0.88 0.87
A (t) 2.33
B (m) 2.08
C (m) 1.79
D (m) 1.65
E (q) 1.52
F (m) 1.30
G (t) 0.88
H (m) 2.28
2.28 0.62 2.08 1.04 3.91 2.04 16.74 3.10
7.5 7.0 6.5 6.0 5.5 5.0 4.5 4.0 3.5 3.0 2.5 2.0 1.5 1.0 0.5
f1 (ppm)

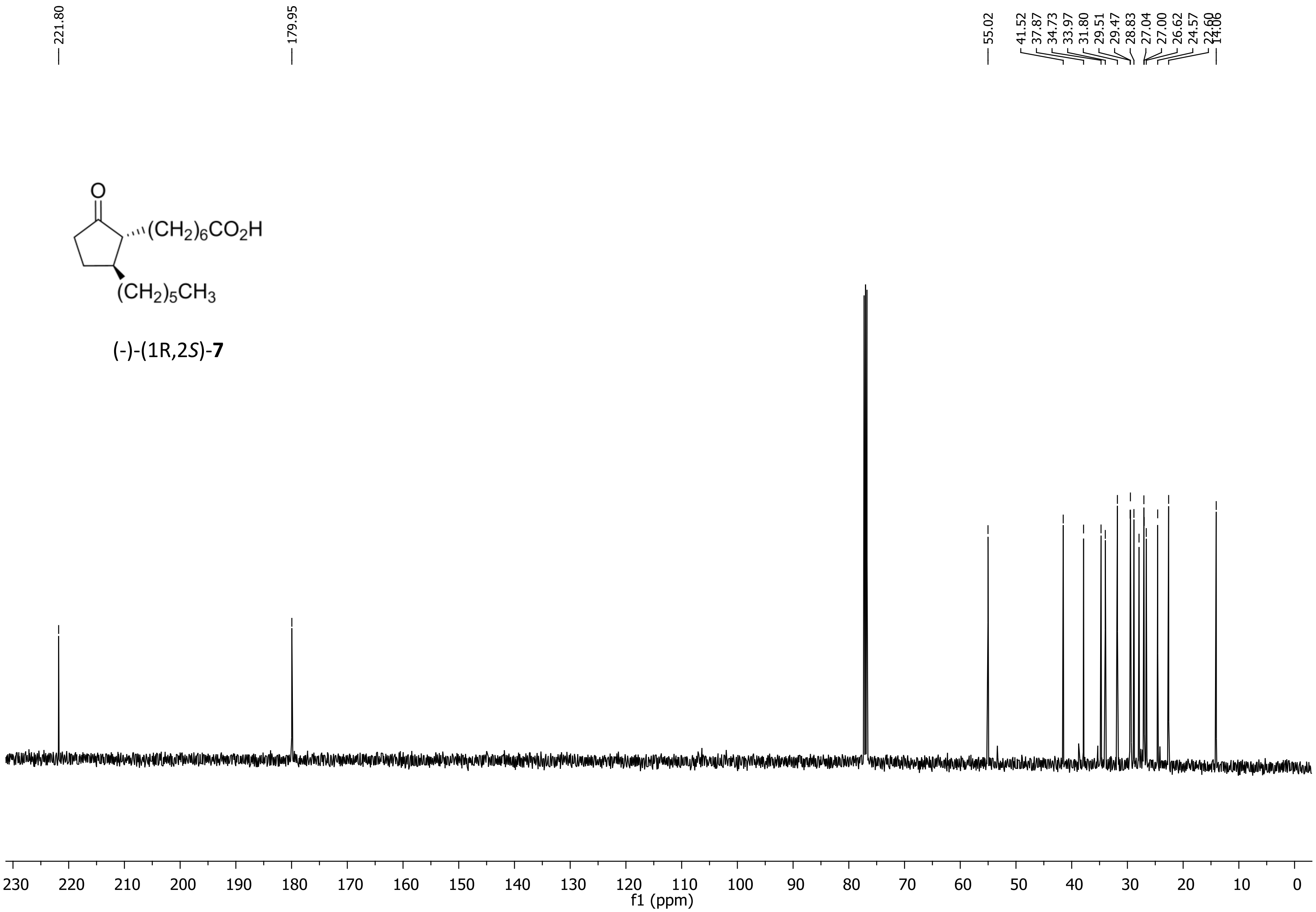
221.80
179.95
55.02
41.52
37.87
34.73
33.97
31.80
29.51
29.47
28.83
27.04
27.00
26.62
24.57
22.60
14.06
O
(CH2)6CO2H
(CH2)5CH3
230 220 210 200 190 180 170 160 150 140 130 120 110 100 90 80 70 60 50 40 30 20 10 0
f1 (ppm)

(-)-(1R,2*S*)-**7**

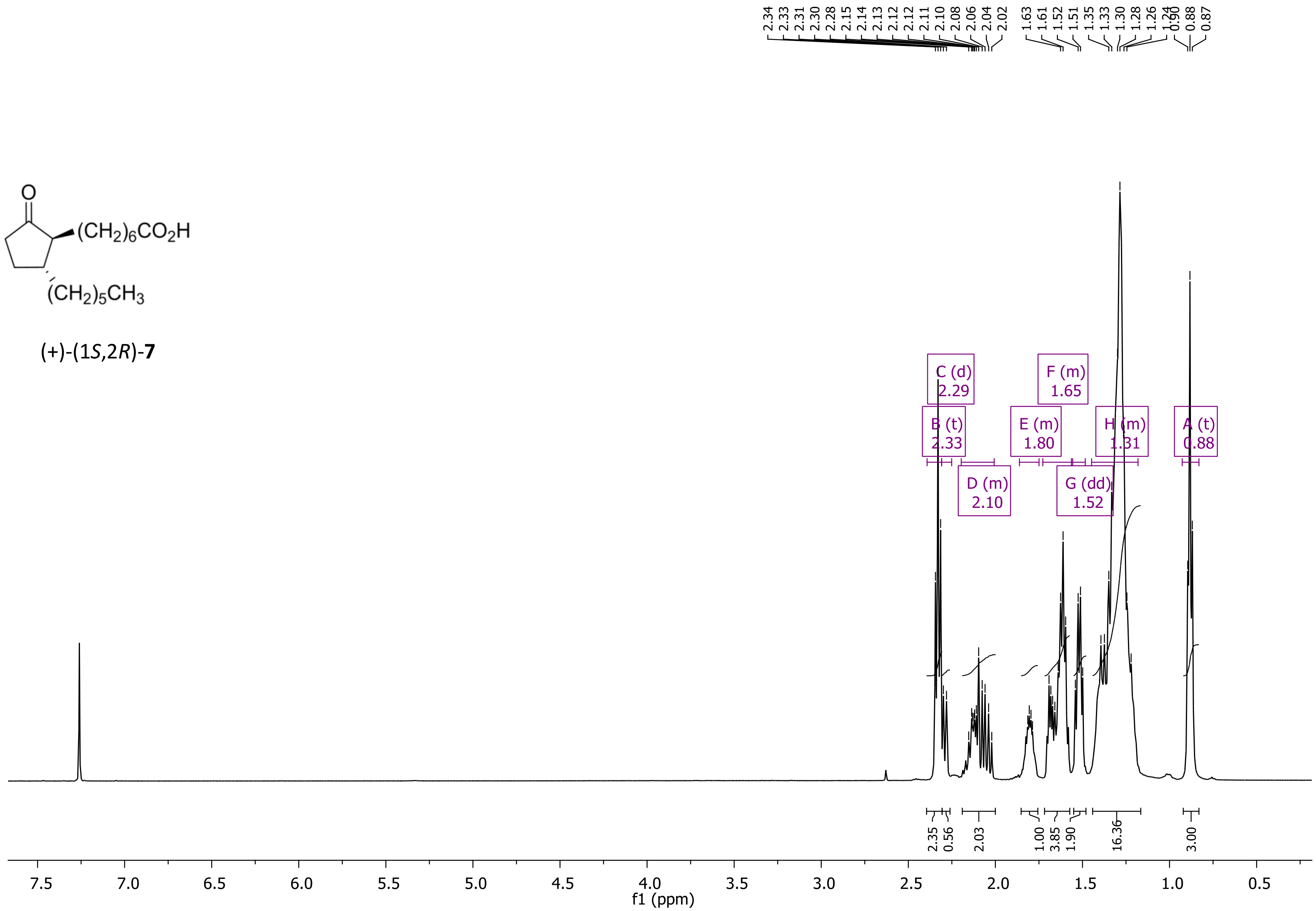

2.34
2.33
2.31
2.30
2.28
2.15
2.14
2.13
2.12
2.12
2.11
2.10
2.08
2.06
2.04
2.02
1.63
1.61
1.52
1.51
1.35
1.33
1.30
1.28
1.26
1.24
0.90
0.88
0.87
C (d)
2.29
B (t)
2.33
D (m)
2.10
E (m)
1.80
F (m)
1.65
G (dd)
1.52
H (m)
1.31
A (t)
0.88
2.35
0.56
2.03
1.00
3.85
1.90
16.36
3.00
7.5
7.0
6.5
6.0
5.5
5.0
4.5
4.0
3.5
3.0
2.5
2.0
1.5
1.0
0.5
f1 (ppm)

(+)-(1*S*,2*R*)-**7**

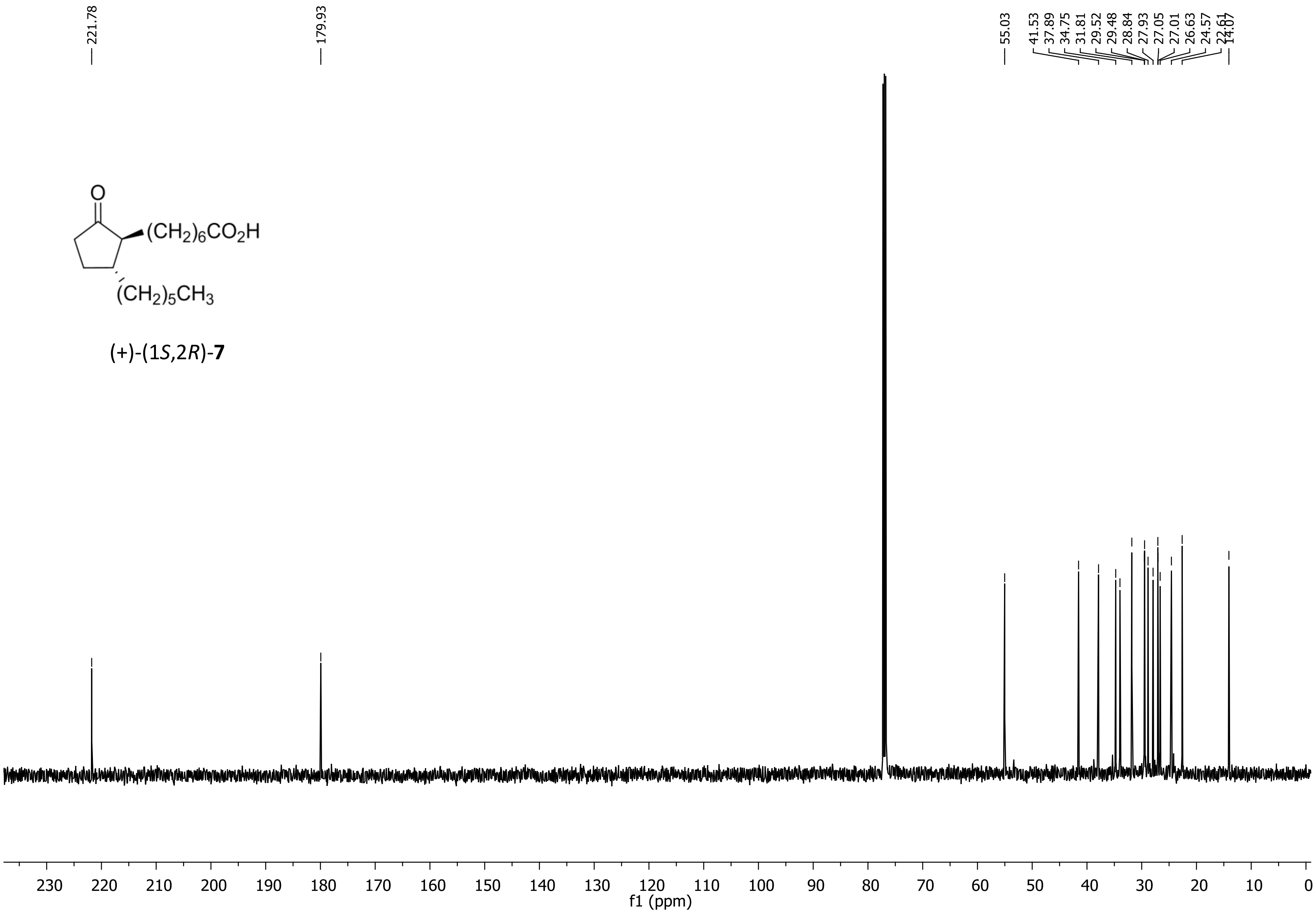
221.78
179.93
55.03
41.53
37.89
34.75
31.81
29.52
29.48
28.84
27.93
27.05
27.01
26.63
24.57
22.61
14.07
O
(CH2)6CO2H
(CH2)5CH3
230 220 210 200 190 180 170 160 150 140 130 120 110 100 90 80 70 60 50 40 30 20 10 0
f1 (ppm)

(+)-(1*S*,2*R*)-**7**

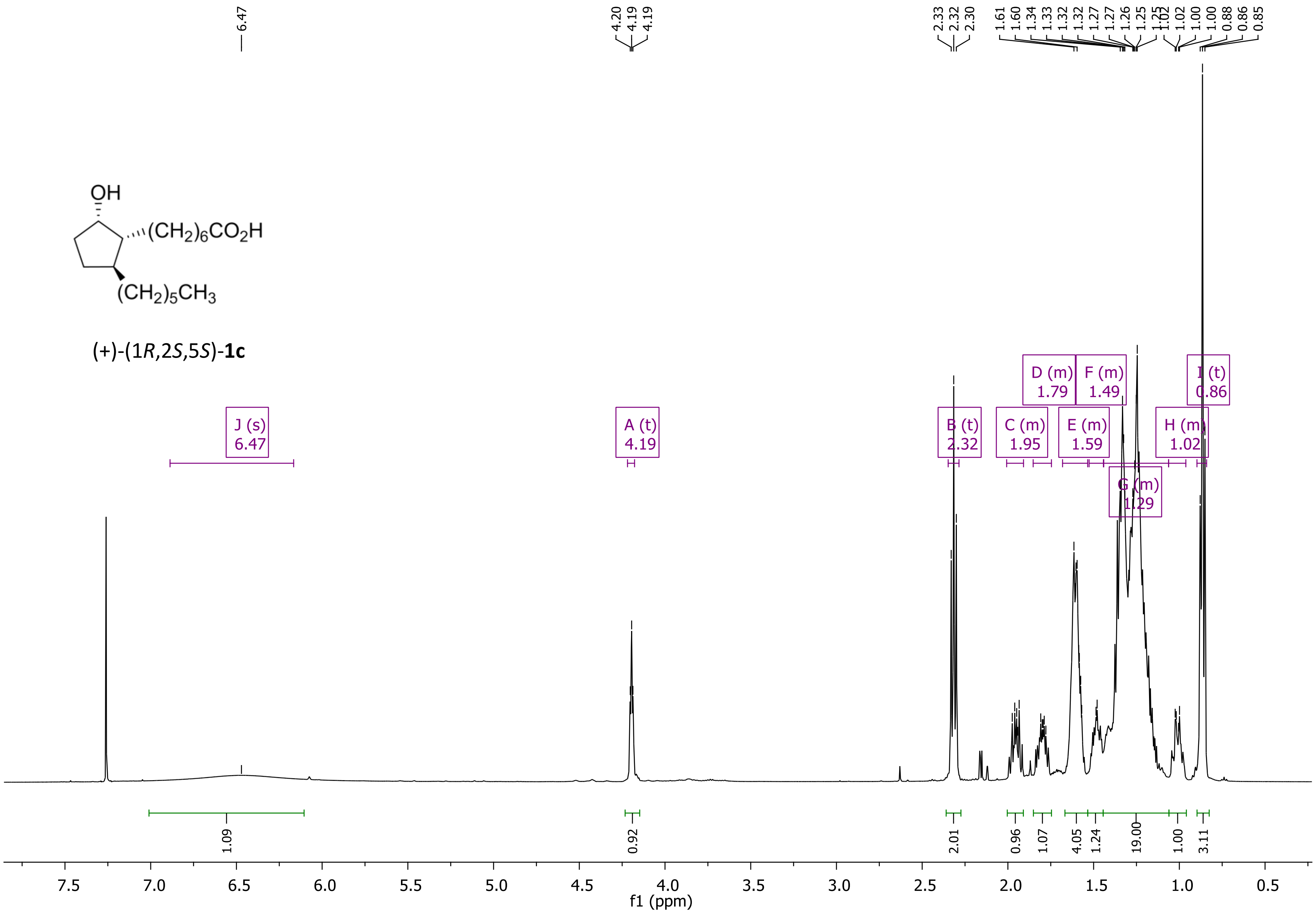
OH
(CH2)6CO2H
(CH2)5CH3
(+)-(1R,2S,5S)-1c
J (s) 6.47
A (t) 4.19
B (t) 2.32
C (m) 1.95
D (m) 1.79
E (m) 1.59
F (m) 1.49
G (m) 1.29
H (m) 1.02
I (t) 0.86
f1 (ppm)

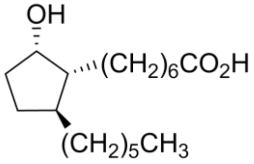
OH
(CH2)6CO2H
(CH2)5CH3

(+)-(1*R*,2*S*,5*S*)-**1c**

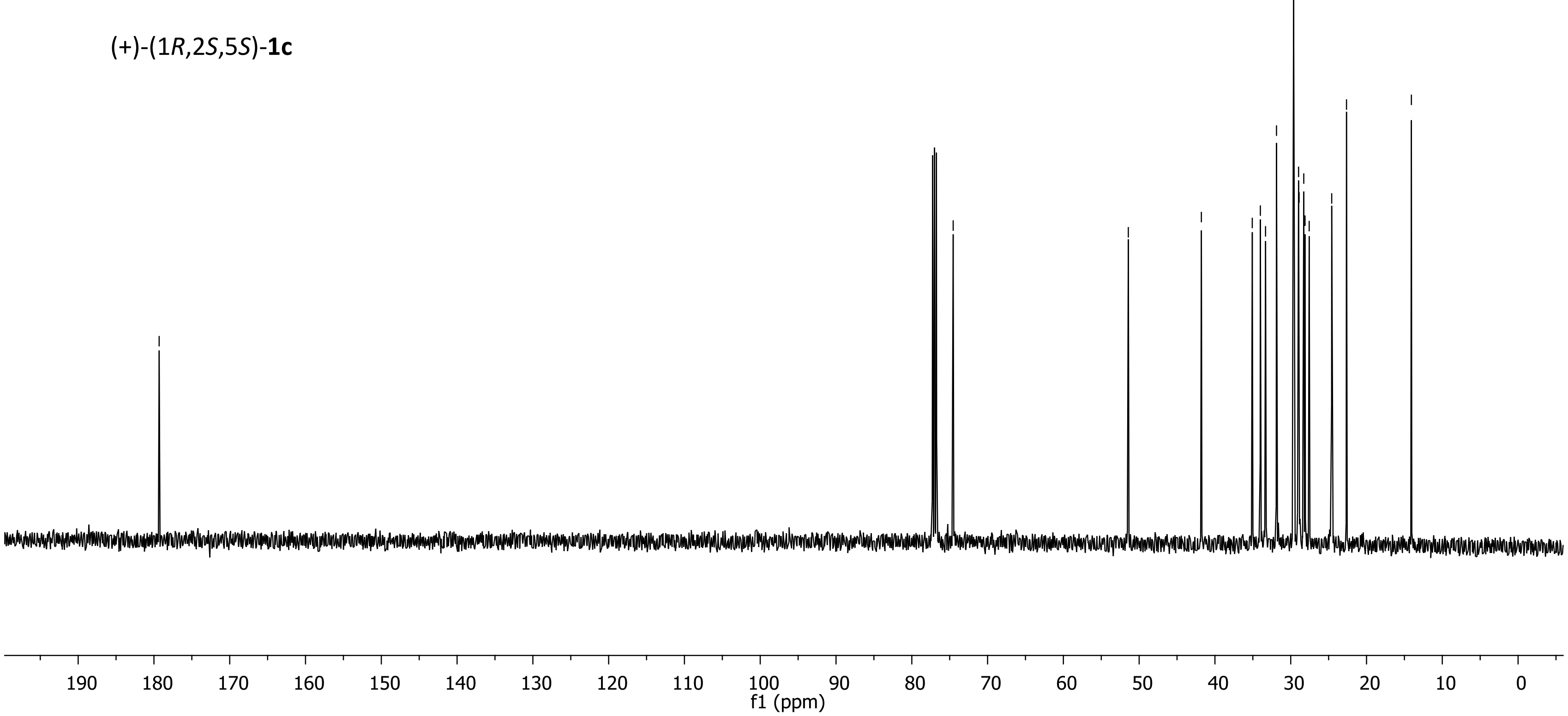
179.31
74.54
51.43
41.79
35.07
34.01
31.88
29.61
28.96
28.89
28.28
28.11
24.59
22.64
14.08
190
180
170
160
150
140
130
120
110
100
90
80
70
60
50
40
30
20
10
0
f1 (ppm)

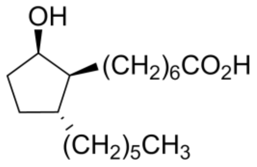

(-)-(1*S*,2*R*,5*R*)-**1a**

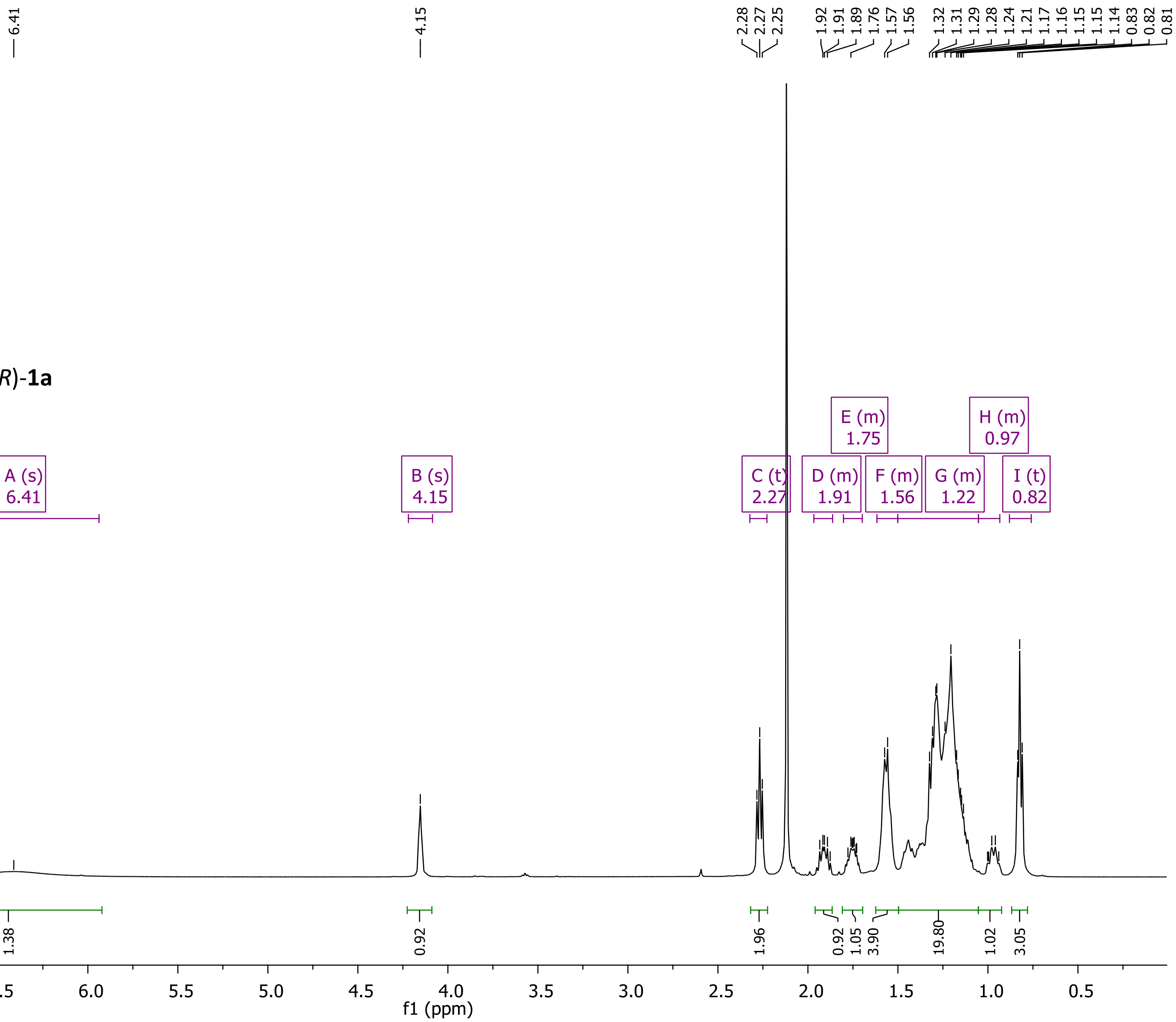

6.41
4.15
2.28
2.27
2.25
1.92
1.91
1.89
1.76
1.57
1.56
1.32
1.31
1.29
1.28
1.24
1.21
1.17
1.16
1.15
1.15
1.14
0.83
0.82
0.81
A (s) 6.41
B (s) 4.15
C (t) 2.27
D (m) 1.91
E (m) 1.75
F (m) 1.56
G (m) 1.22
H (m) 0.97
I (t) 0.82
1.38
0.92
1.96
0.92
1.05
3.90
19.80
1.02
3.05
8.0
7.5
7.0
6.5
6.0
5.5
5.0
4.5
4.0
3.5
3.0
2.5
2.0
1.5
1.0
0.5
f1 (ppm)

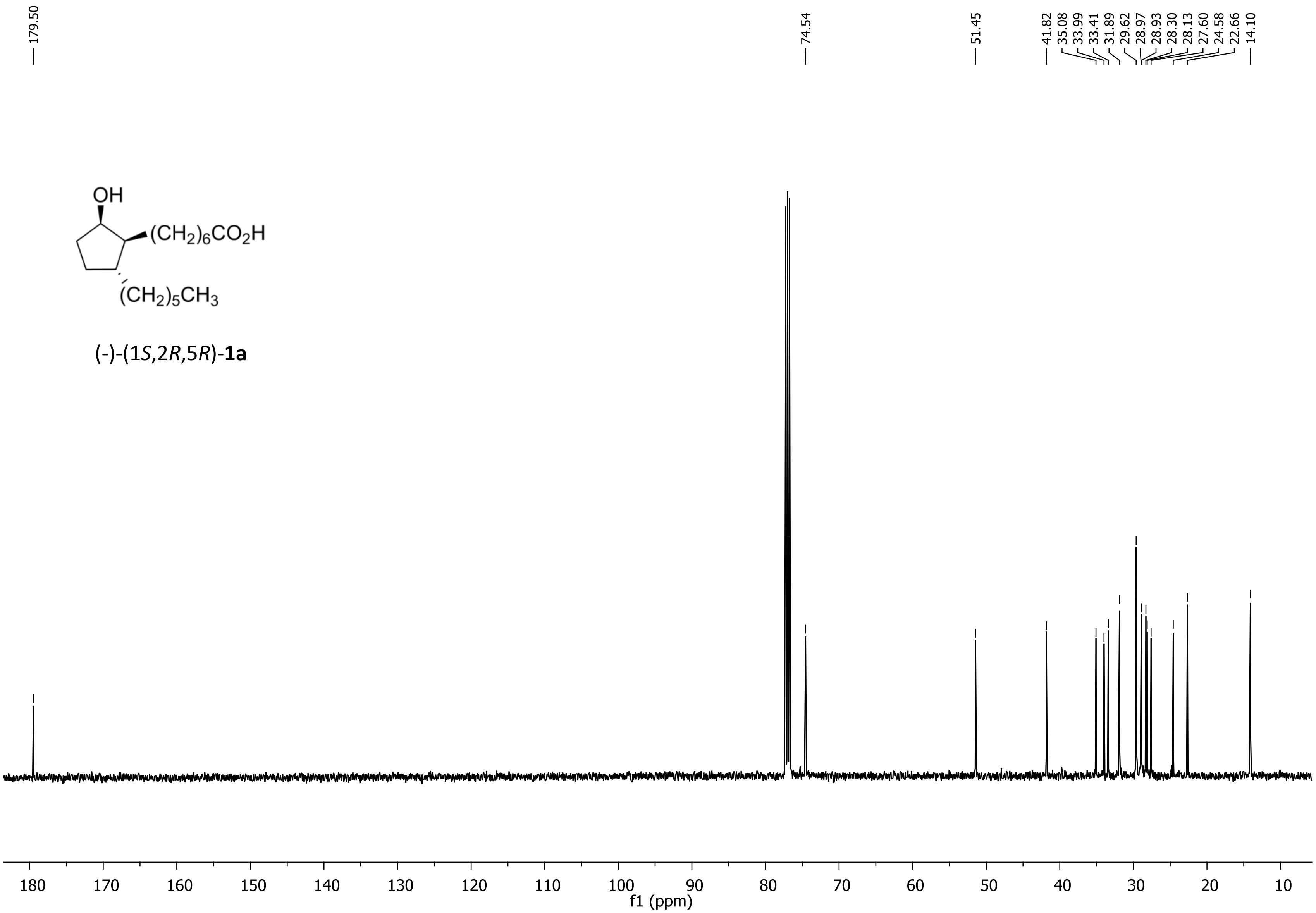
179.50
74.54
51.45
41.82
35.08
33.99
33.41
31.89
29.62
28.97
28.93
28.30
28.13
27.60
24.58
22.66
14.10
OH
$(CH_2)_6CO_2H$
$(CH_2)_5CH_3$
180 170 160 150 140 130 120 110 100 90 80 70 60 50 40 30 20 10
f1 (ppm)

(-)-(1*S*,2*R*,5*R*)-**1a**

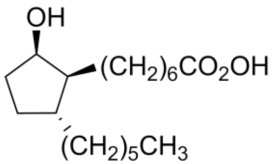
OH
(CH2)6CO2OH
(CH2)5CH3

(-)-(1*S*,2*R*,5*R*)-**8**

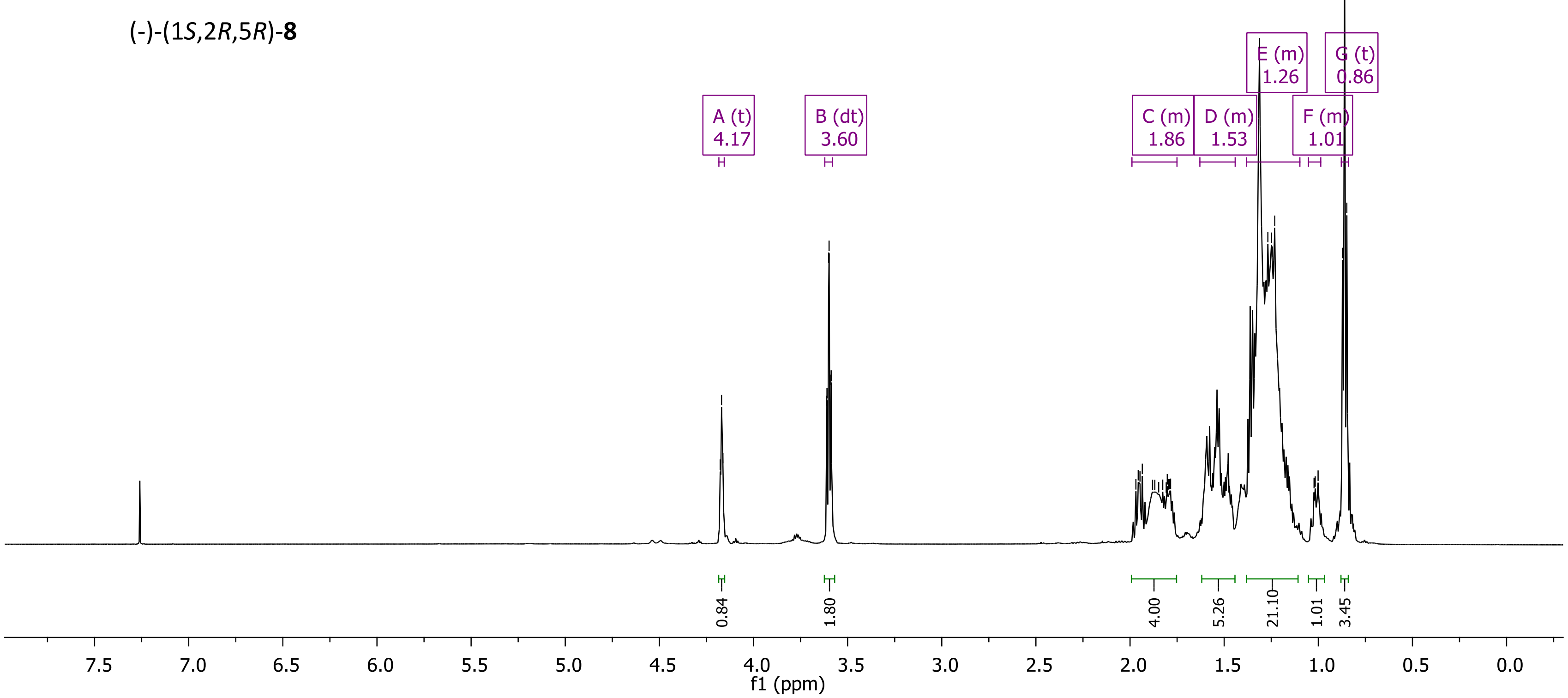
4.18
4.17
4.16
3.61
3.61
3.60
3.60
3.59
3.59
1.97
1.96
1.95
1.93
1.88
1.87
1.85
1.83
1.81
1.80
1.80
1.79
1.78
1.31
1.27
1.25
1.23
1.02
1.02
1.00
0.87
0.86
0.85
A (t)
4.17
B (dt)
3.60
C (m)
1.86
D (m)
1.53
E (m)
1.26
F (m)
1.01
G (t)
0.86
0.84
1.80
4.00
5.26
21.10
1.01
3.45
7.5
7.0
6.5
6.0
5.5
5.0
4.5
4.0
3.5
3.0
2.5
2.0
1.5
1.0
0.5
0.0
f1 (ppm)

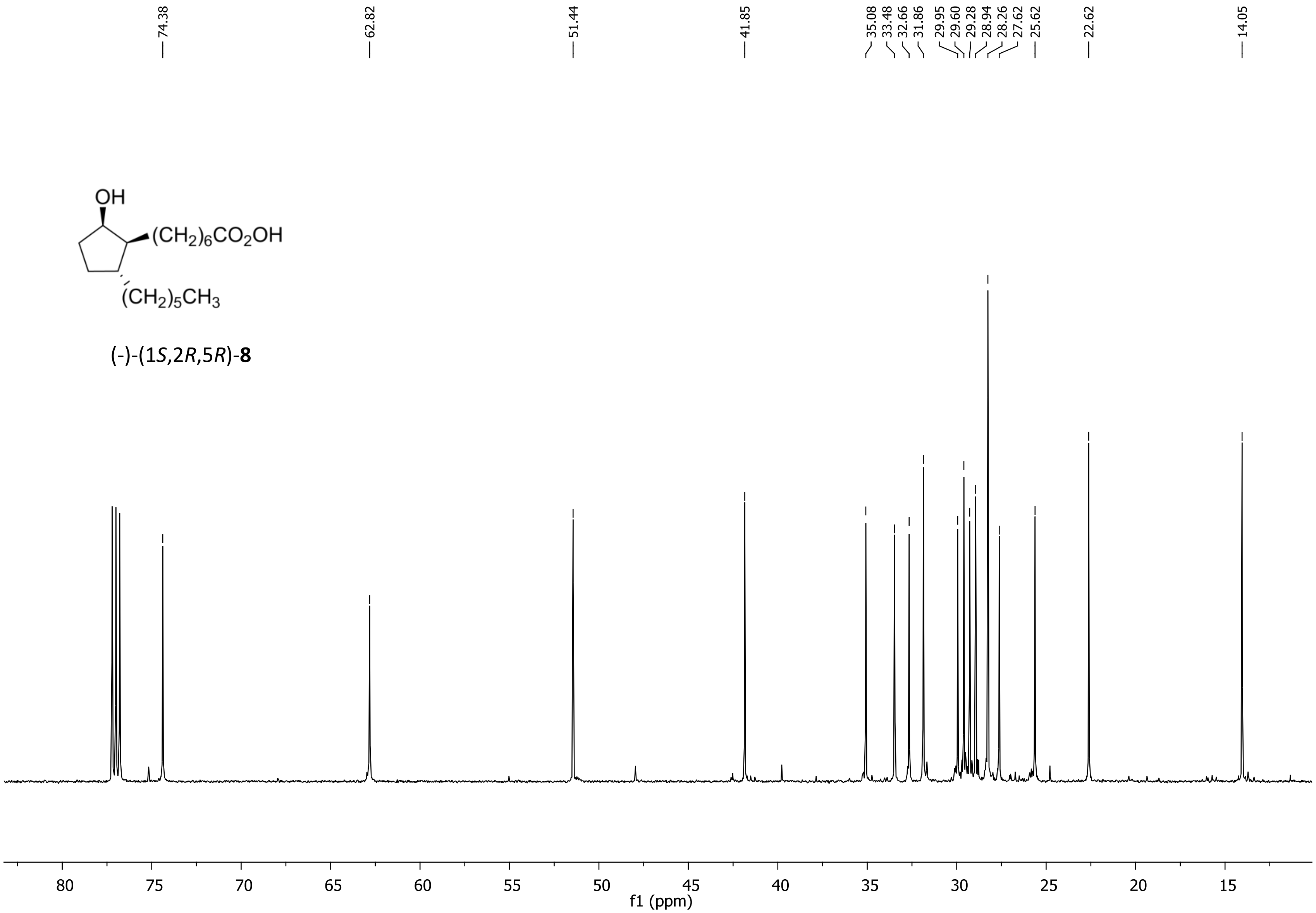
74.38
62.82
51.44
41.85
35.08
33.48
32.66
31.86
29.95
29.60
29.28
28.94
28.26
27.62
25.62
22.62
14.05
OH
(CH2)6CO2OH
(CH2)5CH3
80
75
70
65
60
55
50
45
40
35
30
25
20
15
f1 (ppm)

(-)-(1*S*,2*R*,5*R*)-**8**

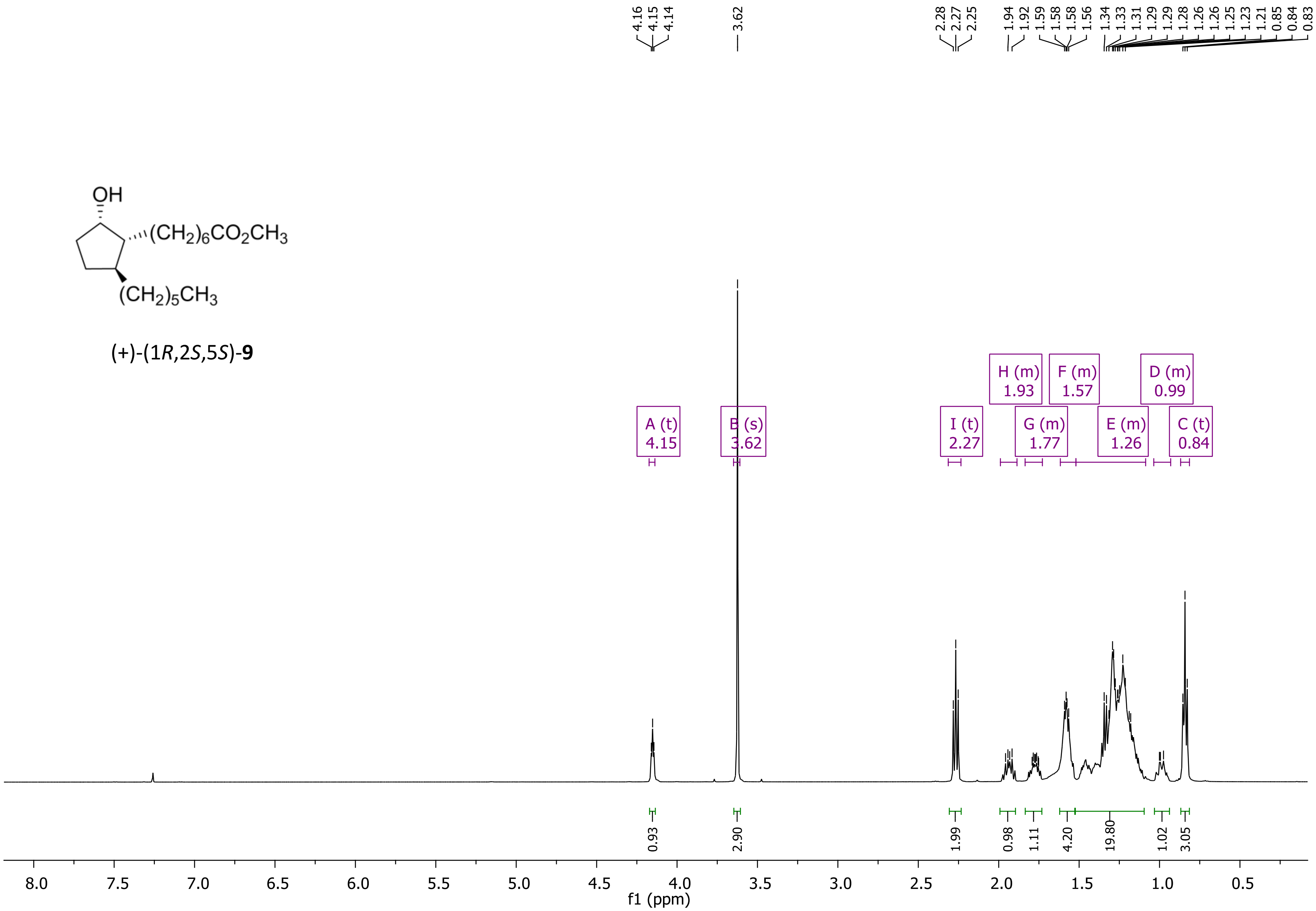
OH
(CH2)6CO2CH3
(CH2)5CH3
(+)-(1R,2S,5S)-9
4.16
4.15
4.14
3.62
2.28
2.27
2.25
1.94
1.92
1.59
1.58
1.58
1.56
1.34
1.33
1.31
1.29
1.29
1.28
1.26
1.26
1.25
1.23
1.21
0.85
0.84
0.83
H (m) 1.93
F (m) 1.57
D (m) 0.99
A (t) 4.15
B (s) 3.62
I (t) 2.27
G (m) 1.77
E (m) 1.26
C (t) 0.84
0.93
2.90
1.99
0.98
1.11
4.20
19.80
1.02
3.05
8.0 7.5 7.0 6.5 6.0 5.5 5.0 4.5 4.0 3.5 3.0 2.5 2.0 1.5 1.0 0.5
f1 (ppm)

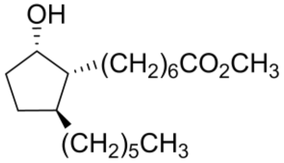

(+)-(1*R*,2*S*,5*S*)-**9**

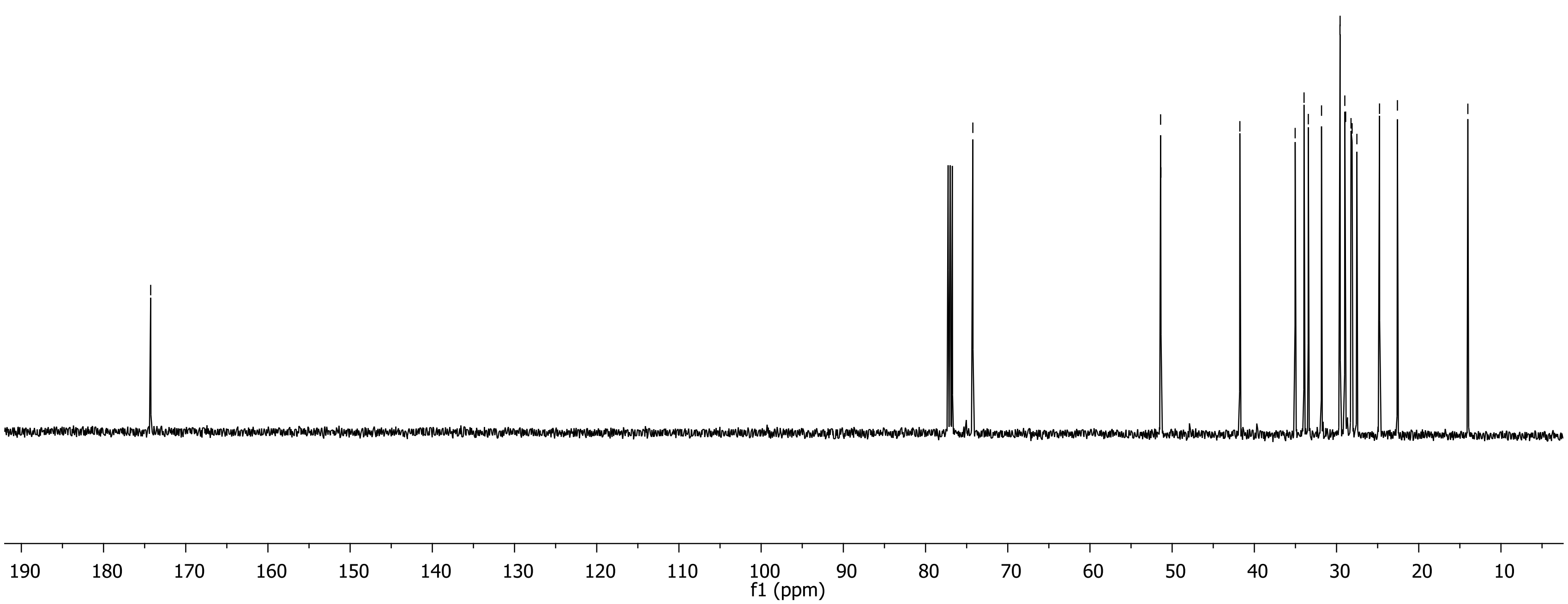
174.26
74.24
51.41
51.37
41.76
33.96
33.44
31.83
29.58
29.57
29.00
28.89
28.24
28.11
24.77
22.60
14.03
190 180 170 160 150 140 130 120 110 100 90 80 70 60 50 40 30 20 10
f1 (ppm)

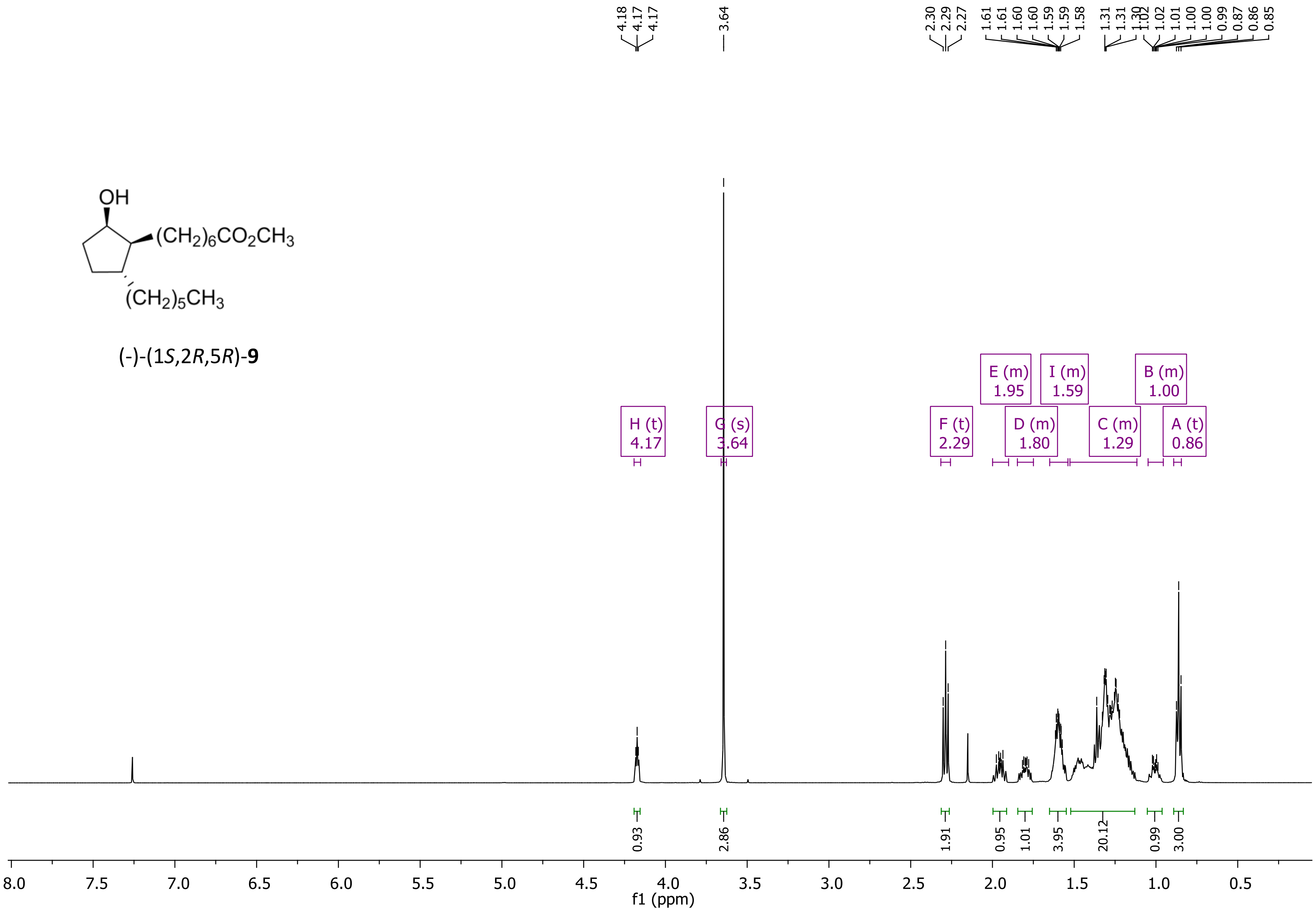

(-)-(1*S*,2*R*,5*R*)-**9**
4.18
4.17
4.17
3.64
2.30
2.29
2.27
1.61
1.61
1.60
1.59
1.59
1.58
1.31
1.31
1.30
1.02
1.02
1.01
1.00
1.00
0.99
0.87
0.86
0.85
E (m) 1.95
I (m) 1.59
B (m) 1.00
H (t) 4.17
G (s) 3.64
F (t) 2.29
D (m) 1.80
C (m) 1.29
A (t) 0.86
0.93
2.86
1.91
0.95
1.01
3.95
20.12
0.99
3.00
8.0
7.5
7.0
6.5
6.0
5.5
5.0
4.5
4.0
3.5
3.0
2.5
2.0
1.5
1.0
0.5
f1 (ppm)

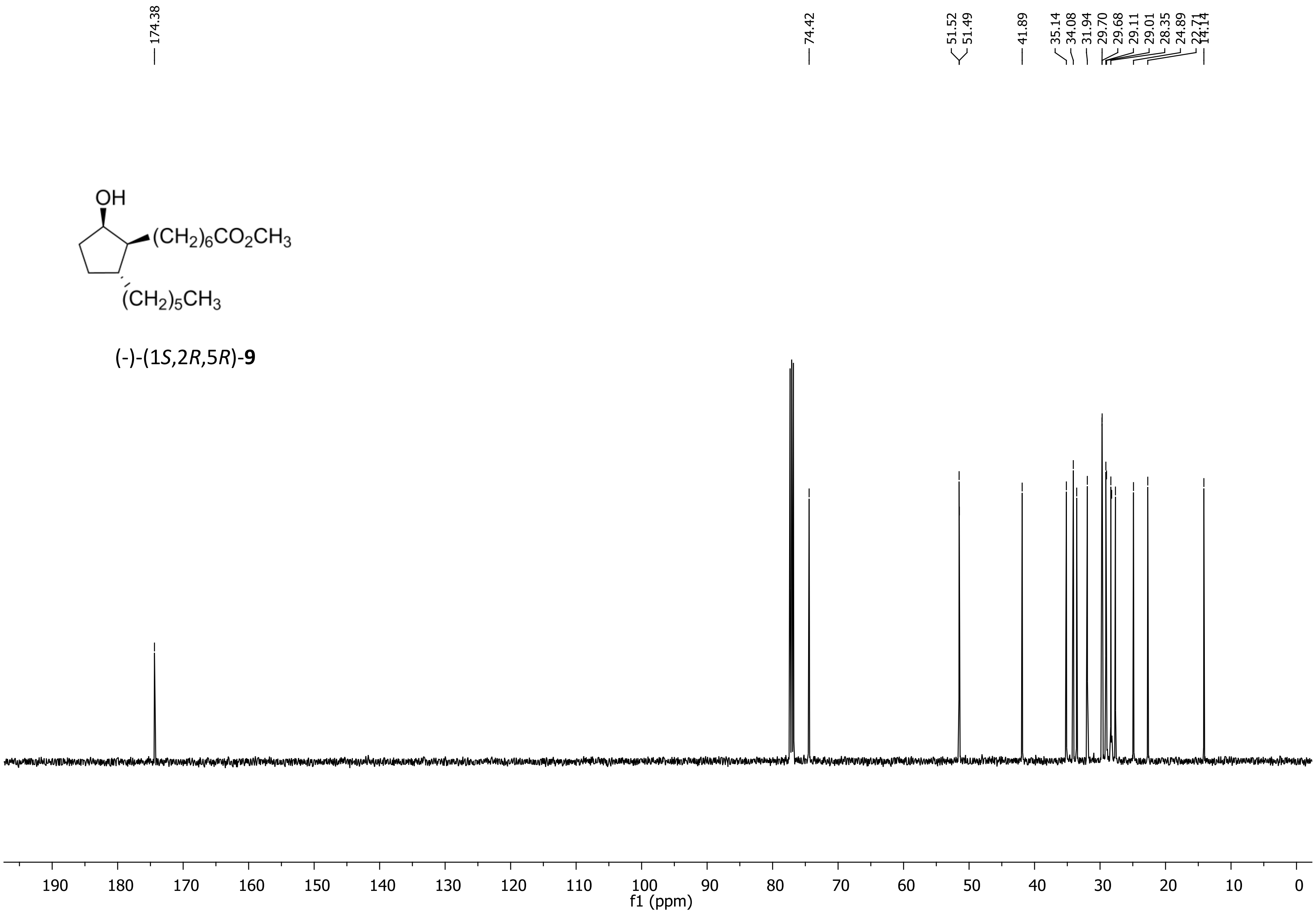
OH
(CH2)6CO2CH3
(CH2)5CH3
174.38
74.42
51.52
51.49
41.89
35.14
34.08
31.94
29.70
29.68
29.11
29.01
28.35
24.89
22.71
14.14
190
180
170
160
150
140
130
120
110
100
90
80
70
60
50
40
30
20
10
0
f1 (ppm)

(-)-(1*S*,2*R*,5*R*)-**9**

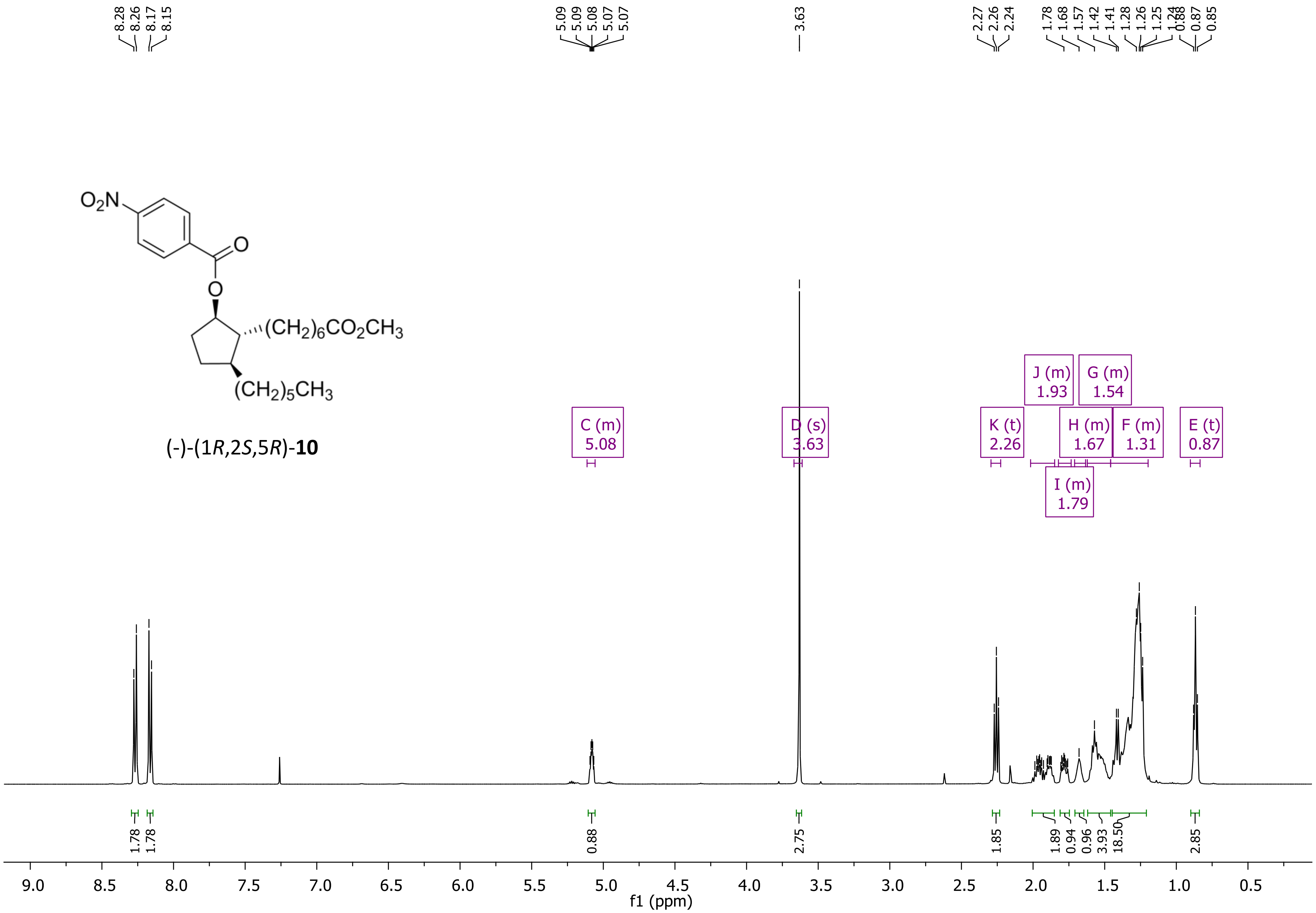

8.28
8.26
8.17
8.15
5.09
5.09
5.08
5.07
5.07
3.63
2.27
2.26
2.24
1.78
1.68
1.57
1.42
1.41
1.28
1.26
1.25
1.24
0.88
0.87
0.85
J (m) 1.93
G (m) 1.54
C (m) 5.08
D (s) 3.63
K (t) 2.26
H (m) 1.67
F (m) 1.31
E (t) 0.87
I (m) 1.79
1.78
1.78
0.88
2.75
1.85
1.89
0.94
0.96
3.93
18.50
2.85
9.0
8.5
8.0
7.5
7.0
6.5
6.0
5.5
5.0
4.5
4.0
3.5
3.0
2.5
2.0
1.5
1.0
0.5
f1 (ppm)

(-)-(1*R*,2*S*,5*R*)-**10**

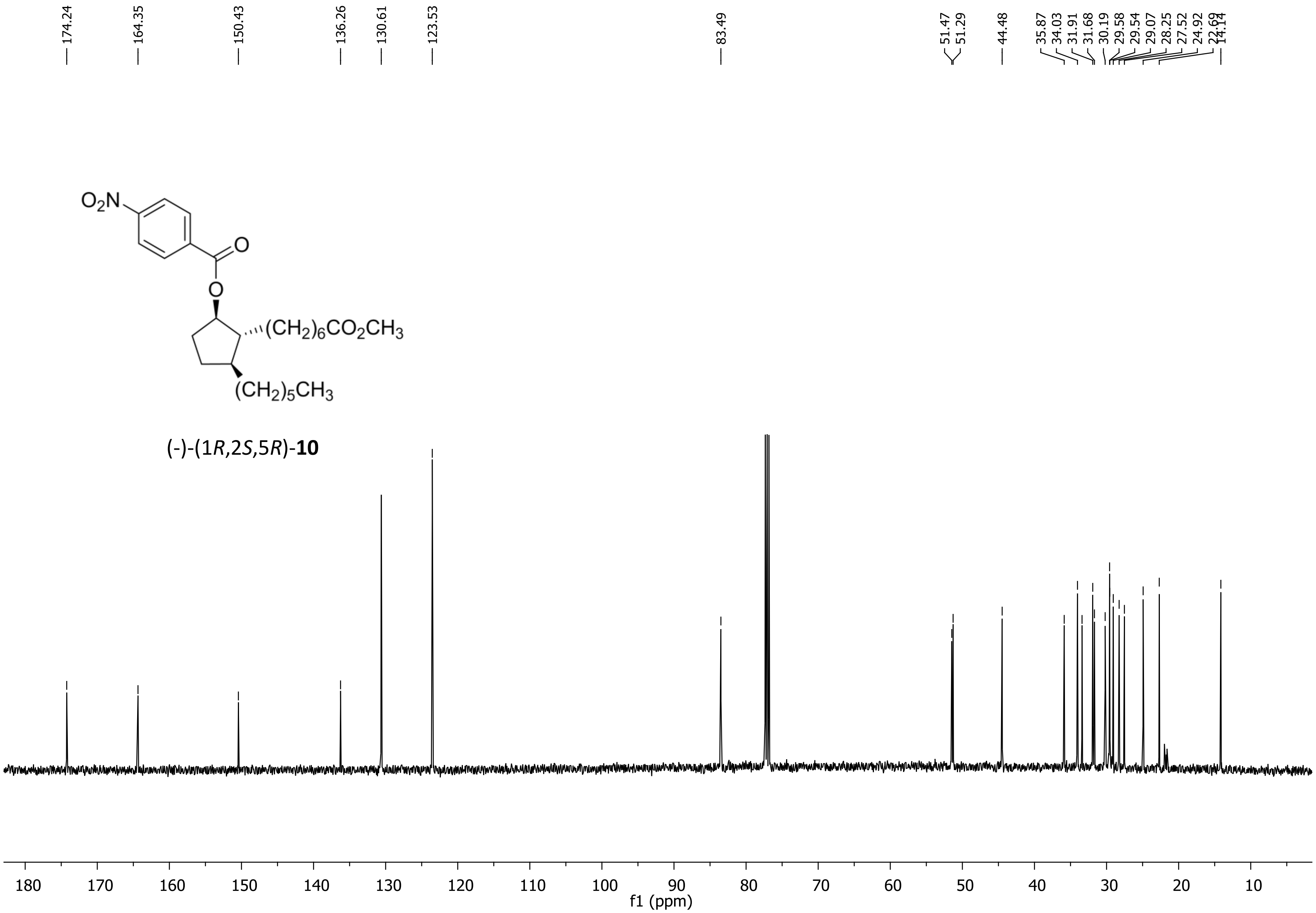

174.24
164.35
150.43
136.26
130.61
123.53
83.49
51.47
51.29
44.48
35.87
34.03
31.91
31.68
30.19
29.58
29.54
29.07
28.25
27.52
24.92
22.69
14.14
O_2N
O
O
$(CH_2)_6CO_2CH_3$
$(CH_2)_5CH_3$
180 170 160 150 140 130 120 110 100 90 80 70 60 50 40 30 20 10
f1 (ppm)

(-)-(1*R*,2*S*,5*R*)-**10**

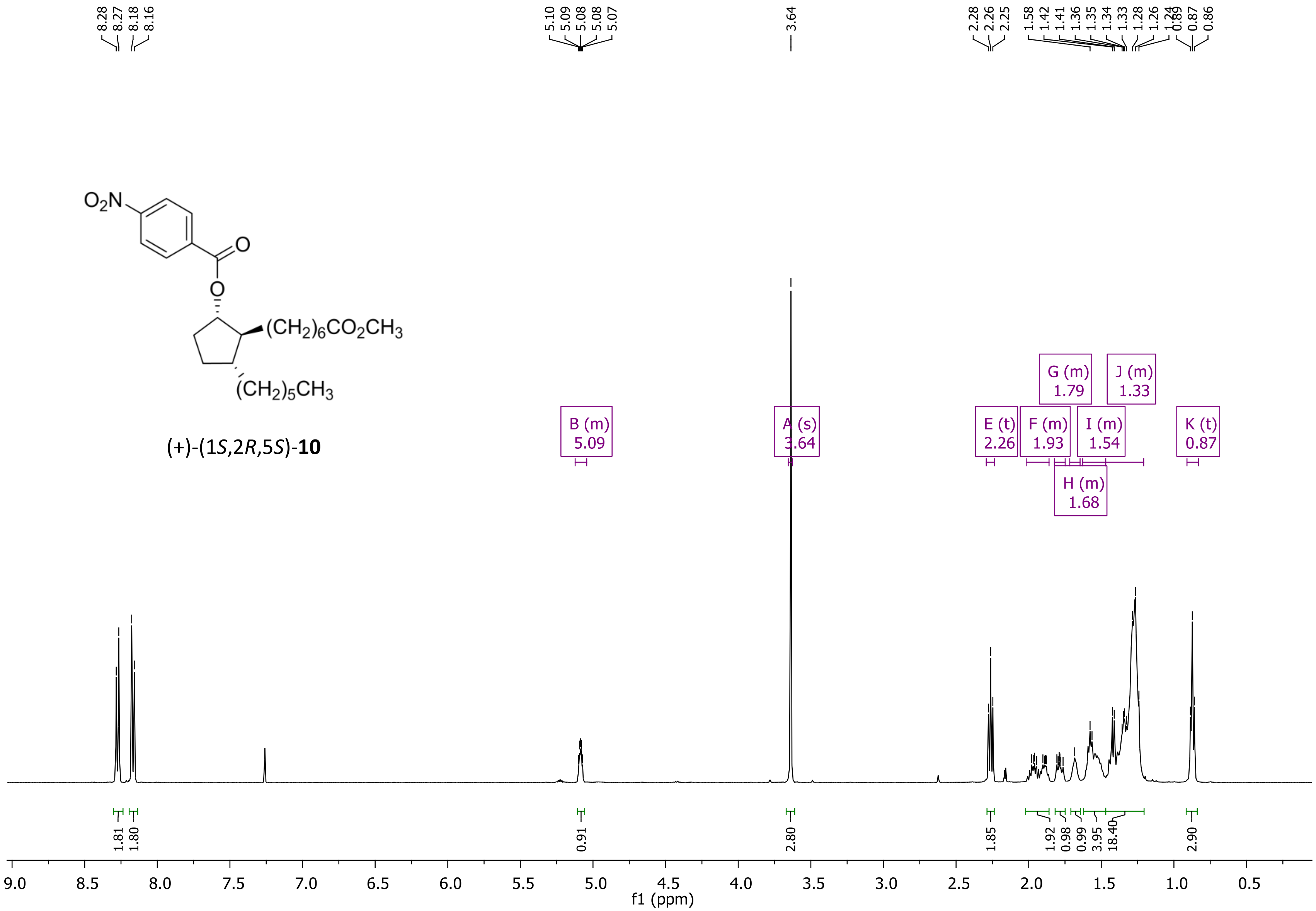
8.28
8.27
8.18
8.16
5.10
5.09
5.08
5.08
5.07
3.64
2.28
2.26
2.25
1.58
1.42
1.41
1.36
1.35
1.34
1.33
1.28
1.26
0.87
0.86
O_2N
O
O
$(CH_2)_6CO_2CH_3$
$(CH_2)_5CH_3$
G (m) 1.79
J (m) 1.33
B (m) 5.09
A (s) 3.64
E (t) 2.26
F (m) 1.93
I (m) 1.54
K (t) 0.87
H (m) 1.68
1.81
1.80
0.91
2.80
1.85
1.92
0.98
0.99
3.95
18.40
2.90
9.0 8.5 8.0 7.5 7.0 6.5 6.0 5.5 5.0 4.5 4.0 3.5 3.0 2.5 2.0 1.5 1.0 0.5
f1 (ppm)

(+)-(1*S*,2*R*,5*S*)-**10**

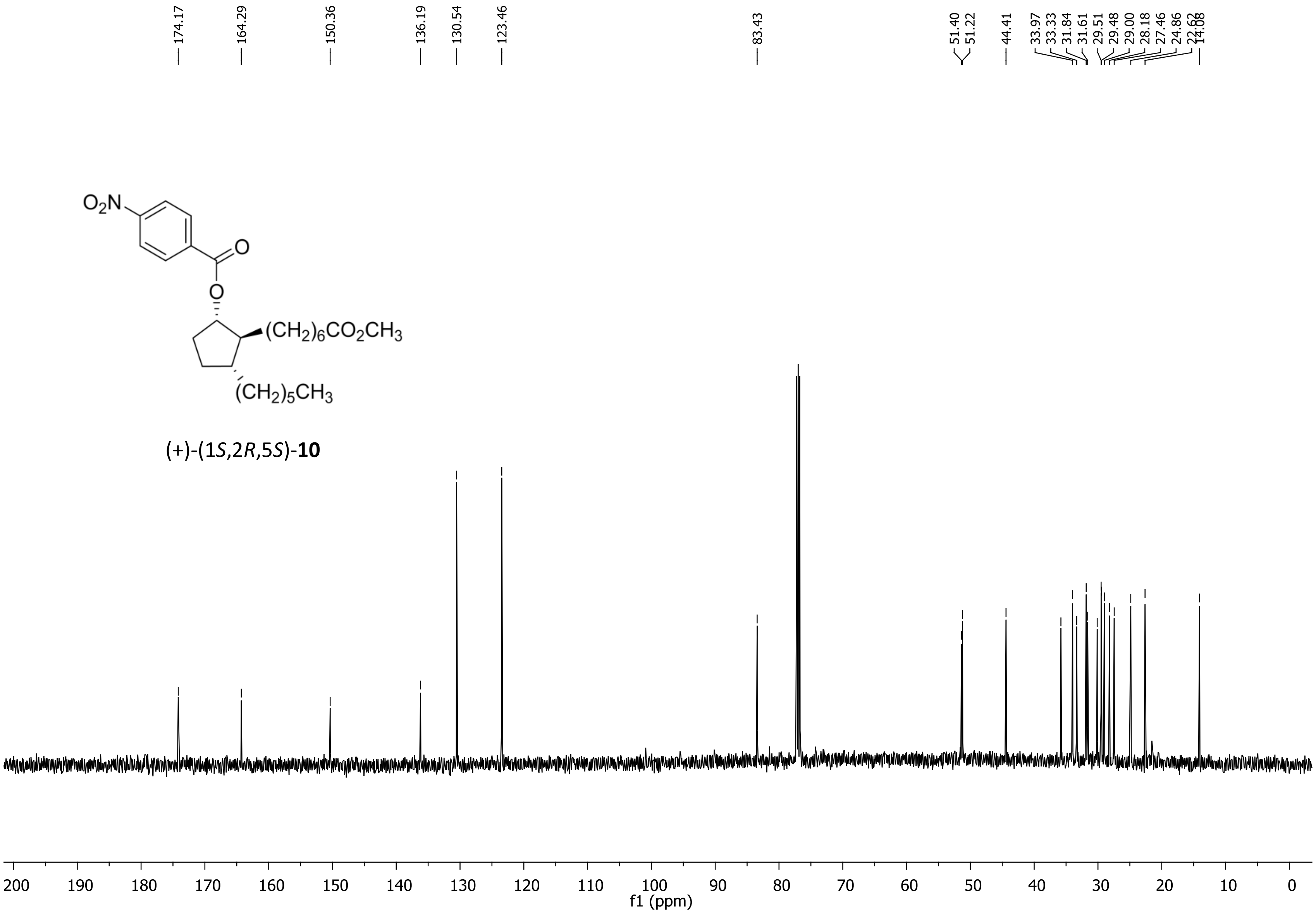
174.17
164.29
150.36
136.19
130.54
123.46
83.43
51.40
51.22
44.41
33.97
33.33
31.84
31.61
29.51
29.48
29.00
28.18
27.46
24.86
22.62
14.08
O2N
O
O
(CH2)6CO2CH3
(CH2)5CH3
200 190 180 170 160 150 140 130 120 110 100 90 80 70 60 50 40 30 20 10 0
f1 (ppm)

(+)-(1*S*,2*R*,5*S*)-**10**

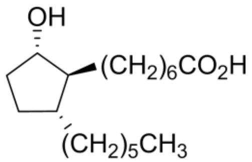

(-)-(1*S*,2*R*,5*S*)-**1b**

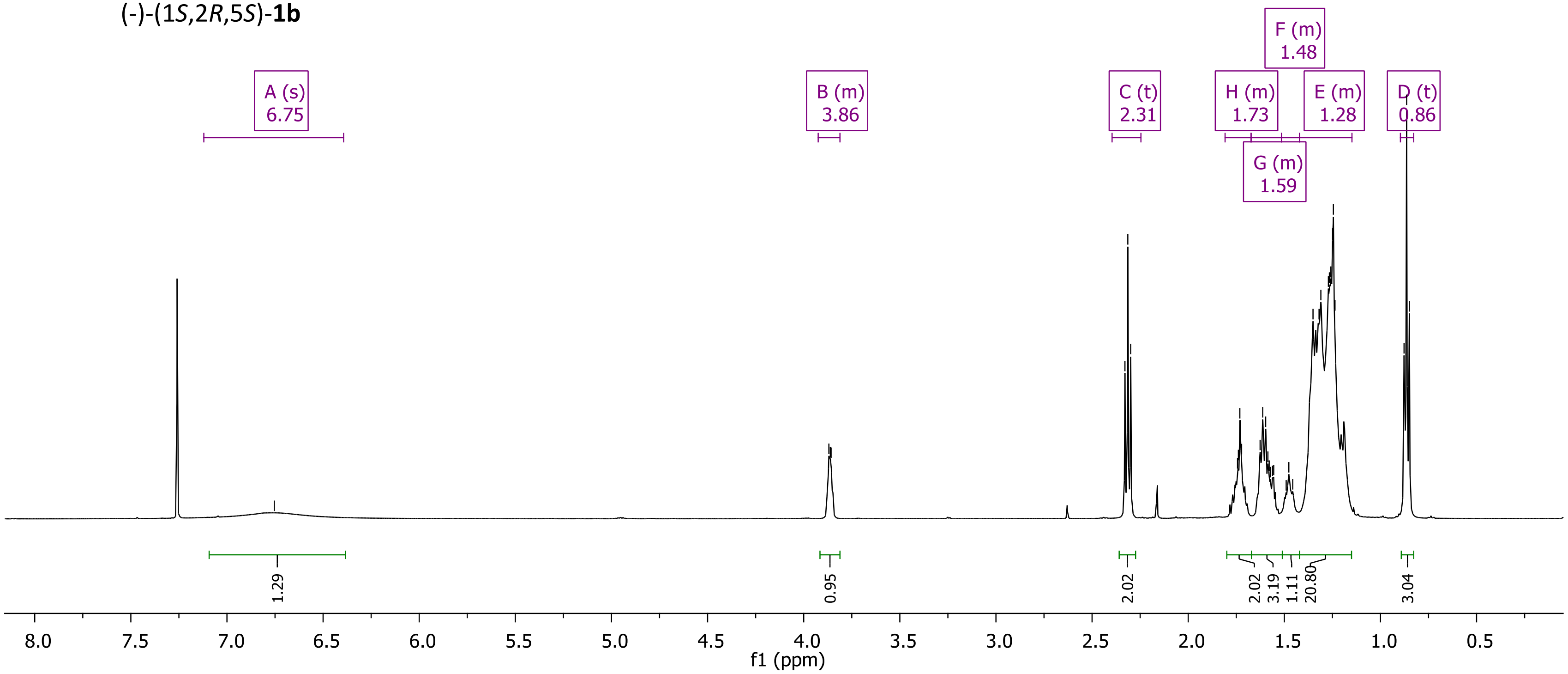

6.75
3.87
3.86
3.86
2.33
2.31
2.30
1.61
1.35
1.32
1.31
1.27
1.27
1.26
1.26
1.25
1.24
1.24
0.88
0.86
0.85
F (m)
1.48
A (s)
6.75
B (m)
3.86
C (t)
2.31
H (m)
1.73
E (m)
1.28
D (t)
0.86
G (m)
1.59
1.29
0.95
2.02
2.02
3.19
1.11
20.80
3.04
8.0
7.5
7.0
6.5
6.0
5.5
5.0
4.5
4.0
3.5
3.0
2.5
2.0
1.5
1.0
0.5
f1 (ppm)

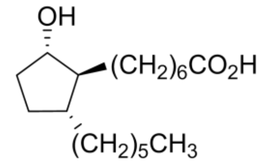
OH
(CH2)6CO2H
(CH2)5CH3

(-)-(1*S*,2*R*,5*S*)-**1b**

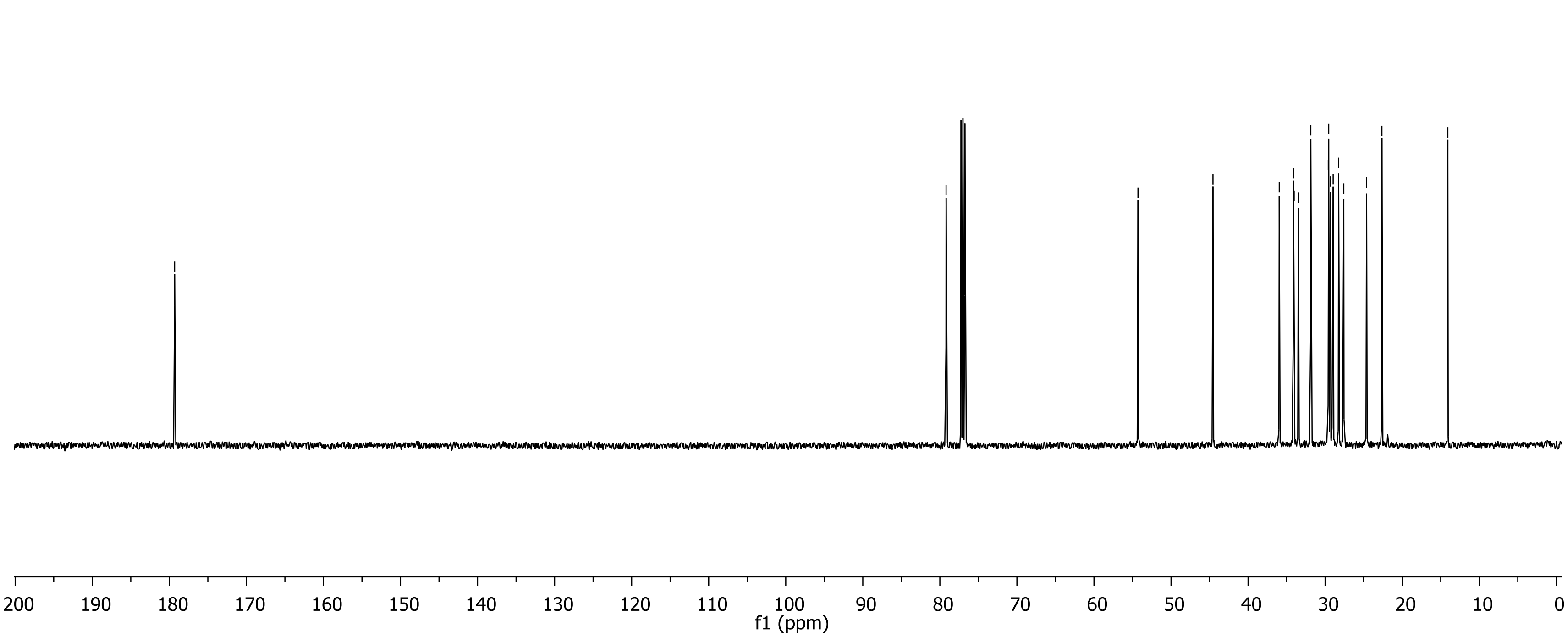
179.31
79.19
54.29
44.56
35.96
34.12
31.86
29.59
29.54
29.32
28.96
28.25
27.58
24.62
22.63
14.08
200
190
180
170
160
150
140
130
120
110
100
90
80
70
60
50
40
30
20
10
0
f1 (ppm)

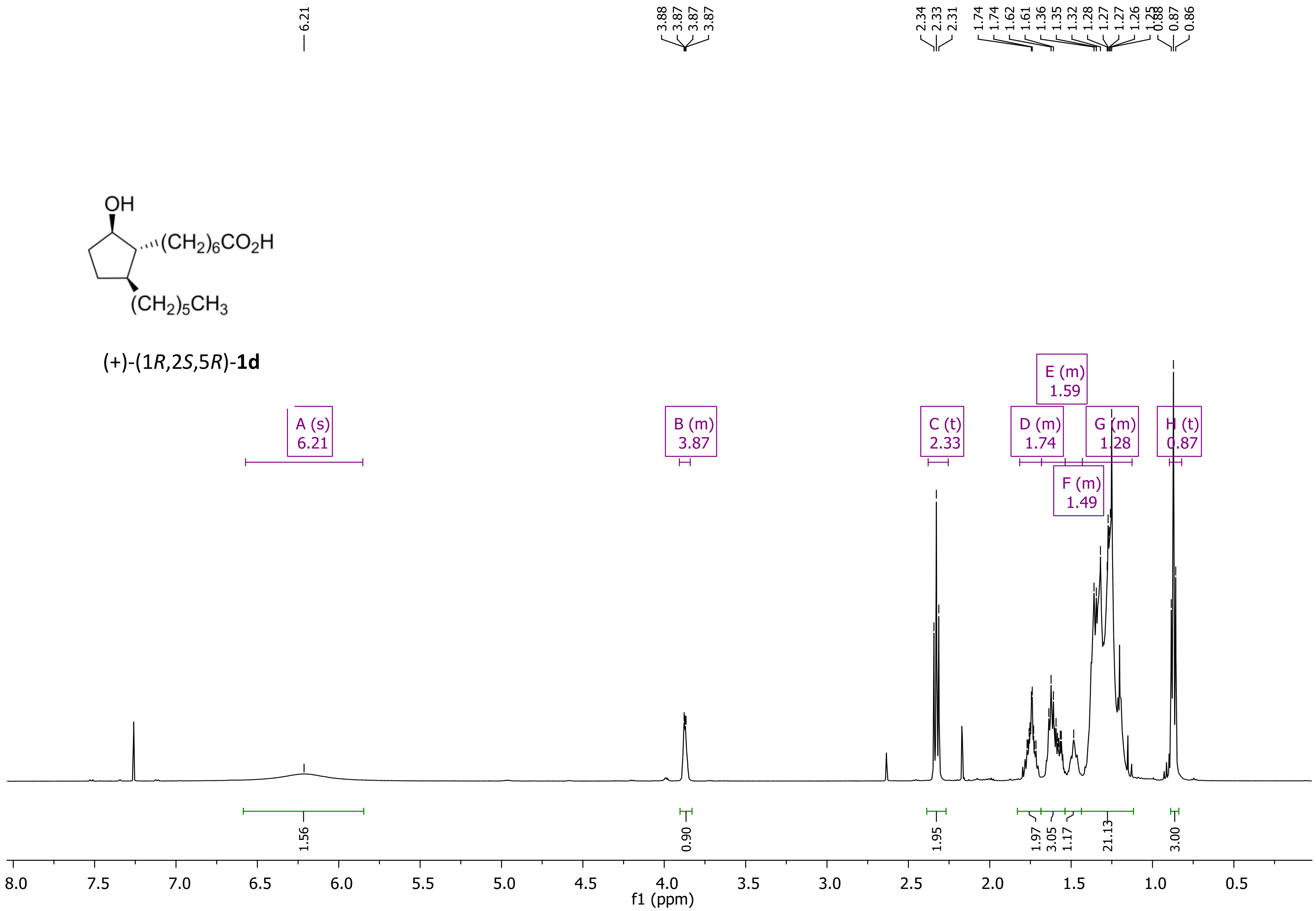
(+)-(1*R*,2*S*,5*R*)-**1d**
OH
(CH2)6CO2H
(CH2)5CH3
6.21
3.88
3.87
3.87
3.87
2.34
2.33
2.31
1.74
1.74
1.62
1.61
1.36
1.35
1.32
1.28
1.27
1.27
1.26
1.25
0.88
0.87
0.86
A (s) 6.21
B (m) 3.87
C (t) 2.33
D (m) 1.74
E (m) 1.59
F (m) 1.49
G (m) 1.28
H (t) 0.87
1.56
0.90
1.95
1.97
3.05
1.17
21.13
3.00
8.0
7.5
7.0
6.5
6.0
5.5
5.0
4.5
4.0
3.5
3.0
2.5
2.0
1.5
1.0
0.5
f1 (ppm)

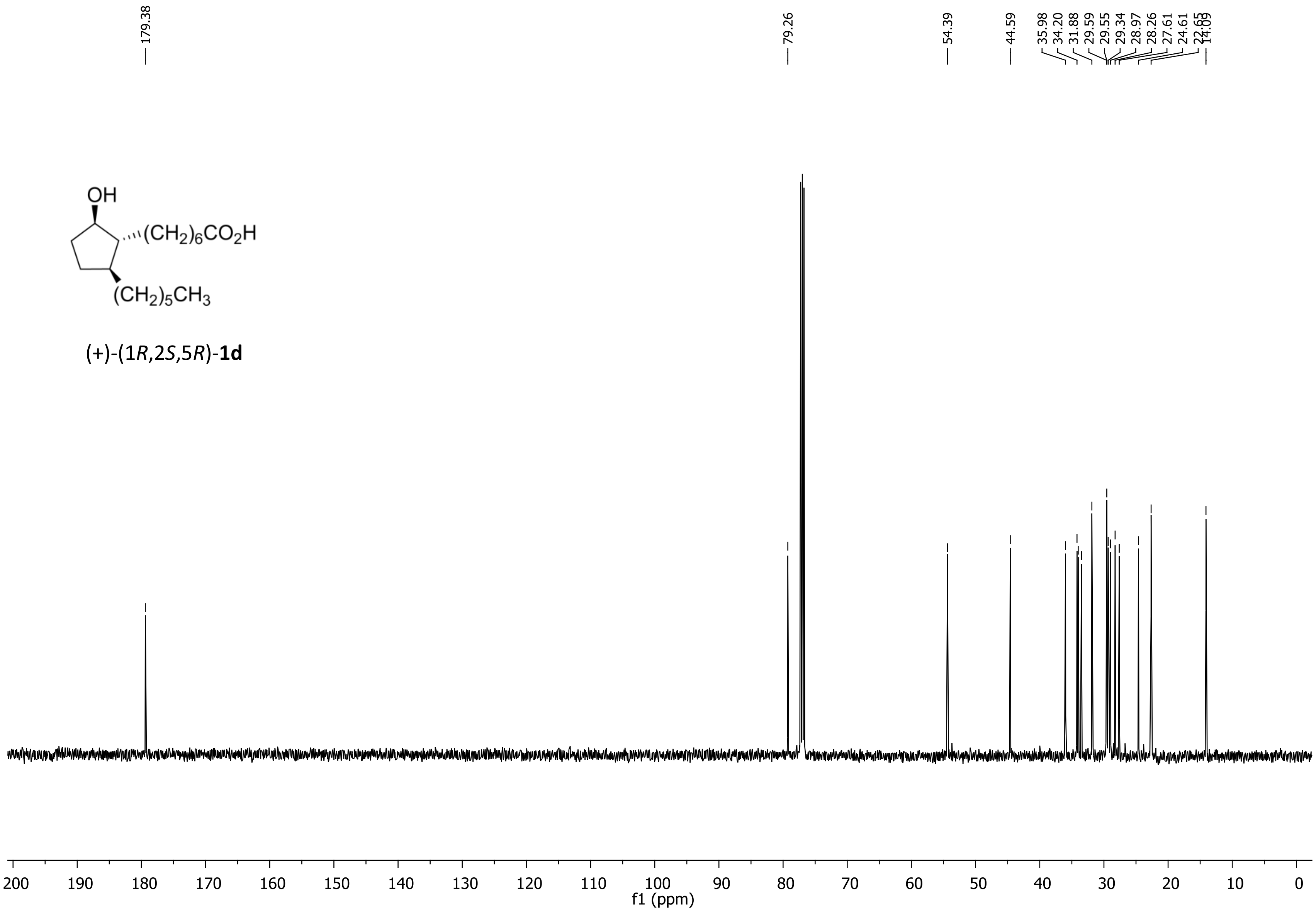
179.38
79.26
54.39
44.59
35.98
34.20
31.88
29.59
29.55
29.34
28.97
28.26
27.61
24.61
22.65
14.09
200 190 180 170 160 150 140 130 120 110 100 90 80 70 60 50 40 30 20 10 0
f1 (ppm)
OH
(CH2)6CO2H
(CH2)5CH3

(+)-(1*R*,2*S*,5*R*)-**1d**
